# Transcript profiling of genes expressed during fibre development in diploid cotton (*Gossypium arboreum* L.)

**DOI:** 10.1186/s12864-017-4066-y

**Published:** 2017-08-31

**Authors:** Atul S. Hande, Ishwarappa S. Katageri, Mangesh P. Jadhav, Sateesh Adiger, Savita Gamanagatti, Kethireddy Venkata Padmalatha, Gurusamy Dhandapani, Mogilicherla Kanakachari, Polumetla Ananda Kumar, Vanga Siva Reddy

**Affiliations:** 1 University of Agricultural Sciences, Dharwad, Karnataka India; 2 National Research Centre on Plant Biotechnology (NRCPB), IARI, New Delhi, India; 3 Plant Transformation Group, International Centre for Genetic Engineering & Biotechnology (ICGEB), New Delhi, India

**Keywords:** Diploid cotton, Microarray, qRT PCR, Transcription factors

## Abstract

**Background:**

Cotton fibre is a single cell and it is one of the best platforms for unraveling the genes express during various stages of fibre development. There are reports devoted to comparative transcriptome study on fiber cell initiation and elongation in tetraploid cultivated cotton. However, in the present investigation, comparative transcriptome study was made in diploid cultivated cotton using isogenic fuzzy-lintless (*Fl*) and normal fuzzy linted (*FL*) lines belong to *Gossypium arboreum,* diploid species at two stages, 0 and 10 dpa (days post anthesis), using Affymetrix cotton GeneChip genome array.

**Result:**

Scanning electron microscopy (SEM) analysis uncovered the occurrence of few fibre cell initials in the *Fl* line as compared to many in Normal *FL* at −2 and 0 dpa. However, at 10 dpa there were no fibre cells found elongated in *Fl* but many elongated cells were found in *FL* line. Up-regulation of transcription factors, AP2-EREBP, C2H2, C3H, HB and WRKY was observed at 0 dpa whereas in 10 dpa transcription factors, AP2-EREBP, AUX/IAA, bHLH, C2H2, C3H, HB, MYB, NAC, Orphans, PLATZ and WRKY were found down regulated in *Fl* line. These transcription factors were mainly involved in metabolic pathways such as phytohormone signaling, energy metabolism of cell, fatty acid metabolism, secondary metabolism and other signaling pathways and are related directly or indirectly in fiber development. Quantitative real-time PCR was performed to check fold up or down-regulation of these genes and transcription factors (TFs) down regulated in mutants as compared to normal at 0 and 10 dpa.

**Conclusion:**

This study elucidates that the up-regulation of transcription factors like AP2-EREBP, C2H2, C3H, HB, WRKY and phytohormone signaling genes at 0 dpa and their down-regulation at the 10 dpa might have constrain the fibre elongation in fuzzy-lintless line. Along with this the down-regulation of genes involved in synthesis of VLCFA chain, transcripts necessary for energy and cell wall metabolism, EXPANSINs, arabinogalactan proteins (AGPs), tubulin might also be the probable reason for reduced growth of fibres in the *Fl*. Plant receptor-like kinases (RLKs), Leucine Rich Repeats) LRR- family protein and signal transduction coding for mitogen-activated protein kinase (MAPK) cascade, have been engaged in coordination of cell elongation and SCW biosynthesis, down-regulation of these might loss the function leads to reduced fibre growth.

**Electronic supplementary material:**

The online version of this article (doi:10.1186/s12864-017-4066-y) contains supplementary material, which is available to authorized users.

## Background

Cotton fibre is one of the prime naturally available raw materials for textile industry. It serves as mainstays for global economy with more than 50% share of raw material. Among all the 50 species of *Gossypium* L. only four species are widely accepted as cultivated, two of these are diploid (2n = 26) and two are allotetraploid (2n = 52) [1–3]. The bulk of world’s cotton is supplied by modem cultivars (Upland cotton) of *Gossypium hirsutum*, represents 90% of the worlds’ cultivated cotton; *Gossypium barbadanse* represents 8% and diploid cotton from the old world i.e., *Gossypium arboreum* and *Gossypium herbaceum* represents remaining 2% [2, 3]. Cotton fibre is the longest single-celled outgrowths in higher plants from individual epidermal cells on the outer integument of developing cotton fruit ovules [3–5]. Cotton fibres of *Gossypium hirsutum* range between 30 and 40 mm in length and ~15 μm in thickness [4, 5]. Recent findings depicts that the cotton is considered as model plant in cell development study and has one of the best characterized single-celled genomics platforms to date [6, 7]. Fibre development in cotton proceeds through a number of distinctive but overlapping stages: fibre initiation, fibre elongation, secondary cell wall deposition (SCWD) [8] and maturation/dehydration [6, 9–11]. In fibre initiation, which occurs around the time of anthesis (from −3 to +1 dpa), although all the cells have potential to undergo morphogenesis and productively differentiate into mature fibres, only 30% becomes mature fibre [11, 12]. Fibre elongation (0 to ~25 dpa) greatly affected by creating and maintaining high turgor pressure within the cells with peak growth rate of 2 mm/day until the fibre gain its final length [13–15]. During the period of secondary cell wall (SCW) biogenesis (~21 to 45 dpa), 90% of cellulose microfibrils deposit on cell wall which gives enough strength and flexibility to the fibres [16]. The accumulation of minerals and simultaneous decrease in water potential associated with the final stage of fibre development, maturation/dehydration (45 to 50 dpa), resulting in a mature cotton fibre [13].

Cotton fiber development is regulated by a network of genes which are associated with the different metabolic pathways such as phytohormone signaling, energy metabolism of cell, fatty acid metabolism, secondary metabolism and other signaling pathways. However, the lack of enough information about the genes and regulatory components that regulate fiber development is one of the main constrain in understanding the genetics to improve cotton fiber quality. Phytohormones such as gibberlic acid [16], auxin [16, 17], ethylene [18] and transcription factors involved in SCW formation such as MYB-TFs [19] and lipid transfer proteins (LTPs) [20] of fatty acid metabolism and brassinosteroids (BR) [16, 21] participate in different stages of fibre development.

Mutants for particular traits are powerful resources for gene expression studies and mutant plants analysis has accelerated the finding and characterization of specific gene function. In the present study, comparative transcriptome analysis was performed in desi fuzzy-lintless (*Fl*) line and normal fuzzy linted (*FL*) line at 0 dpa and 10 dpa using Affymetrix cotton GeneChip genome array. Data from the present study indicates that transcription factors involved in SCW formation and the genes involved in phytohormone-mediated signaling pathways play a vital role in regulating fibre cell initiation and differentiation and the down-regulation of several genes involved in energy metabolism and fatty acid metabolism at 10 dpa may be the probable reason for the reduced growth of fiber in the *Fl*. Transcripts related to signaling i.e. Ca^2+^ and reactive oxygen species (ROS) as well as some heat shock proteins (HSPs) and spermine synthase (SPDS3) were down regulated mainly at 0 dpa lead to reduced fiber growth. This transcriptome analysis found specific genes which are playing role in the metabolic pathways of fibre development that might be useful for further comprehensive analysis of cotton fibre.

## Methods

### Plant materials for microarray and qRT-PCR experiments

The near isogenic *Fl* (Fuzzy-lintless) line was generated by crossing *FL* and *Fl* (recurrent parent) lines at Agriculture Research Station (Cotton), Dharwad Farm, UAS, Dharwad. Morphological trait parameters such as plant height, leaf shape, number of sympodia, number of fruiting bodies, flower colour, fibre length, etc. were observed in both fuzzy-linted (*FL*) and fuzzy-lintless (*Fl*) line. Flowers were tagged with label on the day of anthesis and considered as 0 dpa (days post anthesis). Samples were collected at 0 and 10 dpa in liquid nitrogen and stored at −70 °C until used for total RNA extraction. Samples were harvested and immediately frozen.

### Scanning electron microscopy

Ovule samples were kept in tissue fixative consisting of 3% (*v*/v) glutaraldehyde in 0.1 M sodium phosphate buffer and stored at 0-4 °C (2-4 h) and then washed with 0.1 M phosphate buffer pH = 7.2 (3 × 10 min). The samples were subjected for post-fixation in 1–2% osmium tetroxide* in 0.1 M phosphate buffer** pH = 7.2 (2–4 h) at room temperature and in a light tight container. Dehydration was carried out in a graded acetone series 30% (*v*/v), 50% (*v*/v), 70% (*v*/v), (stored tissue in 70% (*v*/v) acetone for overnight), 80% (*v*/v), 90% (*v*/v), 96% (*v*/v), 100% (*v*/v) for 5–15 min each and finally washed twice with 100% (*v*/v) ethanol (15–30 min each). Samples were mounted on stubs and allowed for metal coating with Gold- Palladium alloy. The SEM images were acquired by Zeiss EVO MA10 (Carl Zeiss, Oberkochen, Germany) at 15 kV EHT. Number of fibre initials was quantified in 100 μm^2^ area using ImageJ software.

### Composition

* **1–2% osmium tetroxide solution:** 0.25 g OsO_4_ (1%) + 25 ml 0.1 M phosphate buffer and washed with 0.1 M phosphate buffer pH = 7.2 (3 × 10 min).

** **0.1 M phosphate buffer:** 15 ml of sodium phosphate monobasic stock (0.5 M) + 60 ml distilled water.

### Total RNA isolation

Spectrum™ Plant Total RNA Kit (Sigma-Aldrich St. Louis, MO) was used to isolate total RNA from samples and column digestion was performed to overcome the problems of genomic DNA contamination. The concentration and quality of total RNA was checked by NanoDrop spectrophotometer (Thermo Scientific, USA) and agarose gel electrophoresis using 1.2% of agarose gel prepared in MOPS buffer. Samples were loaded and the gel electrophoresis was carried out at 70v for 20 min and checked under UV transilluminator by absorbance spectra at 260 and 280 nm.

### Microarray hybridizations and data analysis

The microarray utilized was the commercially available Affymetrix GeneChip® Genome array (Affymetrix Inc., Santa Clara, CA) having 23,977 probe sets representing 21,854 cotton transcripts from a variety of EST databases was used for transcriptome analysis. Three biological replicates were maintained to test the reproducibility and quality of the chip hybridization. Total RNA was isolated at 0 dpa and 10 dpa stage from *FL* and *Fl* lines and used for preparation of cDNA GeneChip® 3′ IVT Express Kit. Array hybridization, staining and washing procedures were carried out according to the manufacturer’s instructions.

Differentially expressed transcripts were analyzed with GeneSpring GX-11.5 software (Agilent Technologies, CA, USA). The Robust Multiarray Average (RMA) algorithm was used for normalization of the data in order to generate single expression value for each probe set. Normalized expression values were log_2_-transformed and unpaired t-test was performed for differential expression analysis. Differentially expressed transcripts (DETs) with false discovery rate (FDR) corrected *p* value ≤0.01 and fold change ≥3 were included for further data analysis. DETs were classified using Cluster 3.0 hierarchical clustering software to profile the gene expression patterns during fibre development. To investigate the functional annotation of transcripts, the consensus sequences of probe sets present in the cotton GeneChip were mapped to the *Arabidopsis* TAIR protein database version 10 (http://www.arabidopsis.org/) by BLASTX with E value cutoff ≤ e^−10^. The DETs were also annotated based on NetAffx annotation data for cotton GeneChip (www.affymetrix.com/). Conserved sequences of all probe sets presented in cotton GeneChip were searched for putative transcription factors related to phytohormone biosynthesis and signal transduction pathways, against the *Arabidopsis* transcription factor database (http://plntfdb.bio.uni-potsdam.de, version 3.0) and *Arabidopsis* hormone database (http://ahd.cbi.pku.edu.cn, version 2.0) respectively, by BLASTX with E value cutoff ≤ e^−10^. Based on MIPS functional catalogue (https://www.helmholtz-muenchen.de/ibis/resourcesservices/genomics/funcat-the-functional-catalogue/index.html) DETs were categorized into functional groups. Further, expressions of differentially regulated cotton transcripts were visualized onto metabolic pathways using MapMan software version 3.5.0 (http://mapman.gabipd.org/ja/mapman-version-3.5.0/) [22]. The data generated in microarray analysis is deposited in the Gene Expression Omnibus database (GEO, http://www.ncbi.nlm.nih.gov/geo) at the NCBI under the Series Accession number GSE52432.

### The quantitative real-time PCR (qRT-PCR) analysis

On the basis of enrichment analysis of biological processes and expression profiles of genes differentially expressed during the initiation and elongation, 16 genes were selected for verification of the microarray data. cDNA was prepared with AffinityScript QPCR cDNA Synthesis Kit (Stratagene, Agilent Technologies, USA), following manufacturer protocol to make up a total reaction volume of 20 μL using 1 μg of total RNA. PrimerQuest software (http://eu.idtdna.com) was used for designing Gene specific qRT-PCR primer (Table 1). The qRT-PCR analysis was then carried out in triplates using a MX 3005P Real-Time PCR system (Stratagene, USA) equipped with a 96 well plates system with the SYBR green PCR Master mix reagent (Stratagene). The *GhPP2A1* gene (Accession No: DT545658) from *Gossypium hirsutum* was used as reference gene to normalize the expression values [23].

**Table 1:** List of genes and TFs validated through qRT-PCR

S. no.	Gene_ID	Fold change	Regulation	Stage	GenBank ID	Closest Arabidopsis homolog	Description	Forward Primer	Reverse Primer
Gene 1	GhiAffx.7054.1.S1_at	16.50139	Up	0 dpa	DW509967.1	AT5G59550.1	zinc finger (C3HC4-type RING finger) family protein	ACTTCCACCATGAGACCTCAAGCA	ATTCGGGTCATGGAGATGTGACGA
Gene 2	GhiAffx.2527.1.S1_s_at	151.18683	Down	0 dpa	DW497370.1	AT5G53120.6	spermidine synthase 3	CAACATGCAGGTGAAATCGGAACC	CAAGCAAACACATTGCTGCGTCAC
Gene 3	Ghi.8931.1.S1_a_at	87.72372	Down	0 dpa	DT457712	AT4G27520.1	unknown	TTCAGCTAAGCCCGTTTACACCCT	TGAAACAACGGGAGTTGAAGAGGC
Gene 4	Ghi.7853.1.S1_at	5.8283057	Down	0 dpa	AF443118.1	AT1G01630.1	Sec14p-like phosphatidylinositol transfer family protein	TCCGCTAAAGCGAGGGAAGAAGAA	GGAACATGCTCGATGCTTTCTCCA
Gene 5	Ghi.7950.1.S1_at	81.1664	Down	10 dpa	AY366083.1	AT5G06720.1	peroxidase 2	CGCTTTGTCCGGTGTTCACACATT	TGTTTGCATATGTCGCGTTGAGGG
Gene 6	GhiAffx.46297.1.S1_s_at	58.77188	Down	10 dpa	AI054544	AT4G17030.1	expansin-like B1	ATCCGTCGATATTTGGCAGGAGGA	TCAACTGATGTTCTCCCGATGGCA
Gene 7	Ghi.3370.1.A1_at	45.29446	Down	10 dpa	DT463939	AT1G33590.1	Leucine-rich repeat (LRR) family protein	AGGCGATAATGGAGGTCCACACAA	CCTGCGGCGATTAATGGGTTCAAT
Gene 8	Ghi.8023.1.S1_at	39.720764	Down	10 dpa	DQ116443.1	AT1G12010.1	2-oxoglutarate (2OG) and Fe(II)-dependent oxygenase superfamily protein	ACCAAAGTCAGCAACTACCCTCCA	TTGAGGAGTTGAAGCCCACTGACT
Gene 10	GhiAffx.19125.1.A1_at	19.172623	Down	10 dpa	DW487672.1	AT1G12780.1	UDP-D-glucose/UDP-D-galactose 4-epimerase 1	AGGTTGGCCATAGACTGTTGCAGA	AAAGTGTCGGTAACCCTCGTCGTT
Gene 11	Ghi.6496.1.S1_a_at	18.636307	Down	10 dpa	CD486227	AT4G02380.1	senescence-associated gene 21	AGAGAAGGTTTCATGGGTTCCCGA	ACTTCTTCAAGAGGGTGGCTCTGA
Gene 12	Ghi.1552.1.S1_s_at	18.200262	Down	10 dpa	DN779868	AT1G47960.1	cell wall/vacuolar inhibitor of fructosidase 1	GCAGATCACAGCGCTGCTTAAAGT	TCGCCTTTCGTGACAGCTTCTACA
Gene 13	Ghi.8448.1.S1_x_at	11.607274	Down	10 dpa	AF521240.1	AT5G12250.1	beta-6 tubulin		
Gene 14	Ghi.6088.2.A1_s_at	10.754808	Down	10 dpa	DV849489	AT3G45640.1	mitogen-activated protein kinase 3	TGAGCAGCAACCATTGGGAGAAGA	ACTTGGACTGGCGCACTTAGAAGA
Gene 15	Ghi.5521.1.A1_s_at	8.330377	Down	10 dpa	DT047436	AT5G01210.1	HXXXD-type acyl-transferase family protein	TCAATAGCTGCCATAGTTTCCGGC	TTGAAGTTGGCAGTTAGGAGTGGC
Gene 16	Gra.2314.1.S1_at	6.476485	Down	10 dpa	CO126415	AT3G13750.1	beta galactosidase 1	CGAGAGGCAATGAACCGTTAGCTT	GGCATAATCACAAGCACCGCAACT

## Results and discussion

### Morphology of fuzzy-lintless (*Fl*) and fuzzy-linted (*FL*) line

Morphological traits of *Fl* line were similar to that of Fuzzy-linted line (*FL*) except for their fuzz and lint which represents less fuzz and normal lint (fibre length-28–30 mm) whereas, more fuzz and no any elongating fibers in fuzzy-lintless (*Fl*) line. There was no difference with respect to leaf colour, shape and hairiness. In both lines leaves were green, okra in shape and medium in hairiness. Stem was pigmented with medium hairiness. Both the genotypes possess medium round bolls and were pale green in colour with pointed nature. With respect to flower also, petal colour, anther colour and pollen colour was yellow, similarly anther filament colour was light yellow and normal sepal shape. The sympodial branching was alternative in both lines. Although they were not perfect isogenic lines, derived from BC_1_F_1_.

**Fig. 1:**
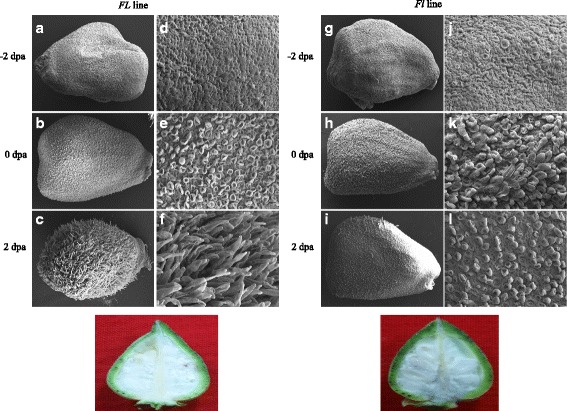
Images of developing fibre initials under Scanning Electron Microscopy (SEM). Images of SEM pictures of complete ovules (**a**, **b**, **c**, **g**, **h**, **i**) and epidermal layer of ovules showing development of fibre initials (**d**, **e**, **f**, **j**, **k**, **l**) in *Gossypium arboreum* (*FL* line) and its near isogenic *FL* line and mature seeds with elongated fibres in *FL* line (M) and in *Fl* line (N) are presented

Ovules of *Fl* line were compared with *FL* line through SEM analysis to investigate the differences in early stages of fibre development and it revealed that the presence of very few fibre cell initials in the *Fl* line as compared to *FL* line (Fig. 1). Fibre initials could be seen two days before anthesis (−2 dpa) as well as on the day of anthesis (0 dpa) in the *FL* line. But, the elongating fibre cells were distinctly observed only in fuzzy-linted (*FL*) line represented in Fig. 1. This implicates the mutant gene functions in an early stage of fibre cell differentiation.

### Transcriptome and cluster analysis of differentially expressed transcripts

**Fig. 2:**
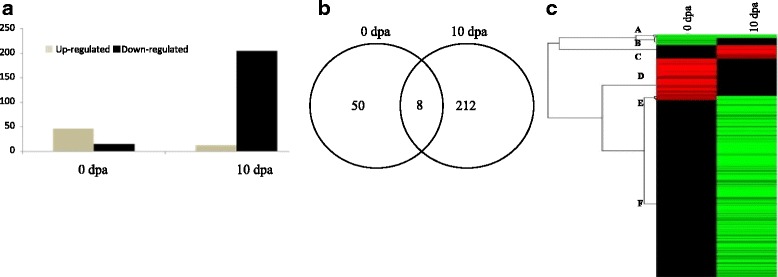
Transcriptome analysis during fibre development stages normal fuzzy linted (*FL*) lines and fuzzy-lintless (*Fl*) line. **a** Number of differentially expressed transcripts (DETs) in *Fl* line as compared to their respective stages in *FL* line at fibre initiation (0 dpa) and elongation (10 dpa) stages. **b** Venn diagram showing the commonly up- and down regulated transcripts between 0 and 10 dpa stages in *Fl* line. **c** Hierarchical cluster analysis of differentially expressed transcripts (fold change ≥3) in *Fl* line as compared to their respective stages in *FL* line at 0 dpa 10 dpa. A to E indicates the five major clusters. Cluster A: Commonly down regulated transcripts at 0 and 10 dpa in *Fl* line Cluster B: Only down regulated transcripts at 0 dpa in *Fl* line (8 DETs) Cluster C: Only up-regulated transcripts at 10 dpa in *Fl* line (15 DETs) Cluster D: Only up-regulated transcripts at 0 dpa in *Fl* line (42 DETs) Cluster E: Up-regulated at 0 dpa and down regulated at 10 dpa in *Fl* line (4 DETs) Cluster F: Only down regulated transcripts at 10 dpa in *Fl* line (197 DETs)

To study the fibre development, a genome wide expression analysis was carried out at 0 and 10 dpa using ovules of fuzzy-linted line (*FL*) and fuzzy-lintless (*Fl*) line. Labeled RNA was hybridized to Affymetrix cotton GeneChip Genome array. After statistical analysis, transcripts with *p* value ≤0.01 and fold change ≥3.0 were considered as differentially expressed in *Fl* line and the number of DETs identified at 0 and 10 dpa in *Fl* line are compared with their respective stages in *FL* line (Fig. 2a). Maximum number of transcripts (220, 79.14% of total DETs) showed differential expression at 10 dpa whereas, only few transcripts (58, 20.86% of total DETs) showed differential expression at 0 dpa. 8 DETs were found common in both 0 and 10 dpa (Fig. 2b).

Further to profile the gene expression patterns during fibre development, 278 DETs were classified using hierarchical clustering software Cluster 3.0. The expression patterns were separated into six major clusters (A–F) based on tree branching (Fig. 2c). Transcripts expressed in *Fl* line as compared to normal line (*FL*) during each stage within each cluster are presented as Cluster A representing 4 DETs commonly down regulated at 0 dpa and 10 dpa; Cluster B represents 8 DETs down regulated at 0 dpa; Cluster C represents 15 DETs up-regulated only at 10 dpa; Cluster D represents a maximum of 42 DETs up-regulated at 0 dpa; Cluster E represents 4 DETs up-regulated at 0 dpa but found down regulated at 10 dpa whereas, Cluster F depicts a maximum of 197 DETs down regulated at 10 dpa. These results indicate that differential expression of some genes between *Fl* and *FL* may control fibre cell initiation and elongation. In the following studies, the key genes responsible for such difference between these lines are mentioned clearly.

### Annotation and functional classification of DETs

**Fig. 3:**
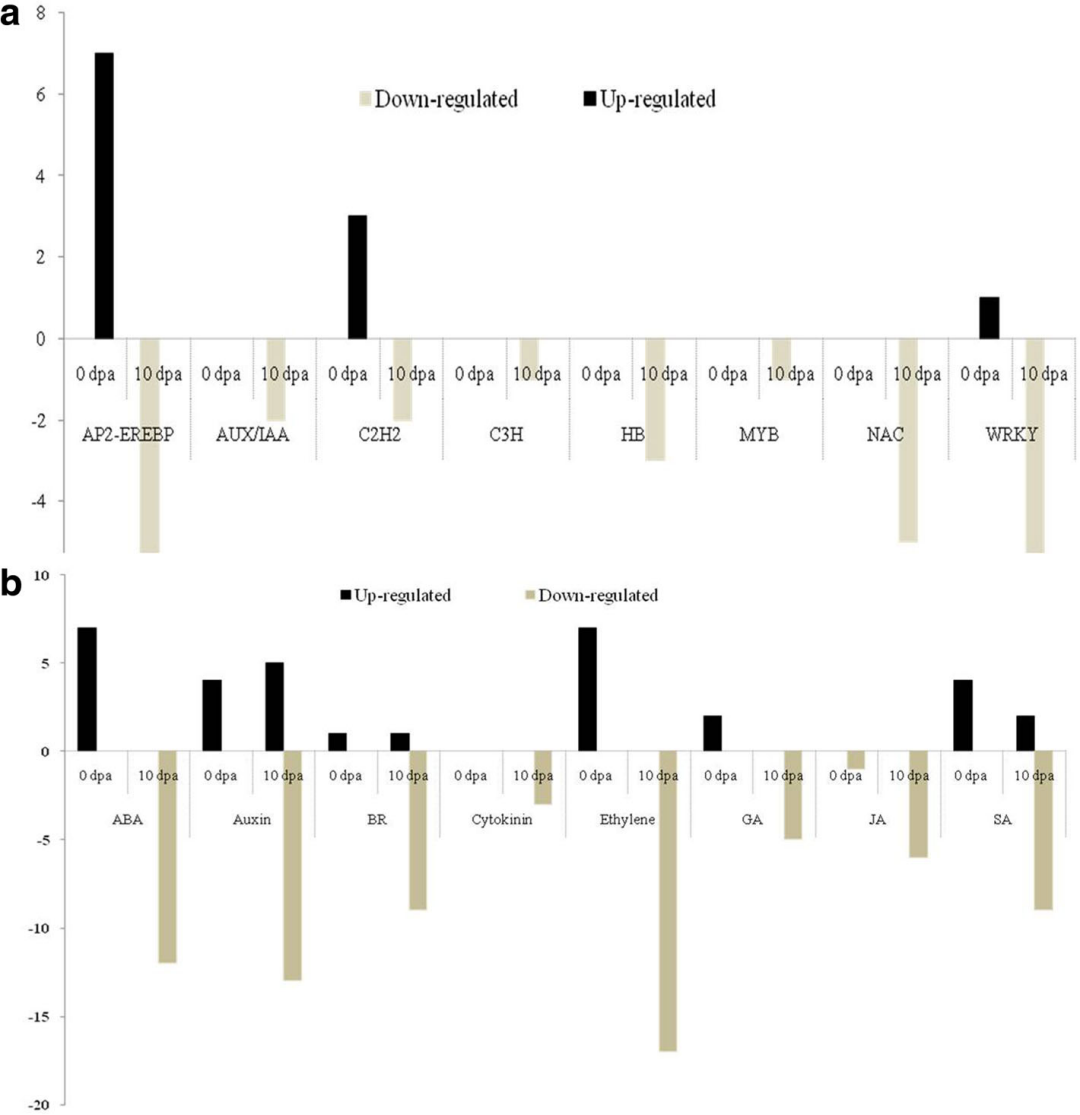
Analysis of differently expressed transcripts (DETs) related to transcription factors (TFs) and phytohormone signaling (PHs) in *Fl* line compared to their respective stages in the *FL* line at fibre initation (0 dpa) and elongation (10 dpa) stages. **a** Number of DETs encoding TFs of various families in *FL* line at 0 and 10 dpa. **b** Number of DETs related to the phytohormone signaling in *Fl* line at 0 and 10 dpa

DETs were annotated based on TAIR database (http://www.arabidopsis.org). It was observed that out of 278 DETs, 239 (85.97%) were matched with *Arabidopsis* gene models with *E* value ≤ e^−10^ (Additional files 1 and 2). Further, classification the DETs based on MIPS functional catalogue (https://www.helmholtz-muenchen.de/ibis/resourcesservices/genomics/funcat-thefunctional-catalogue/index.html) and DETs related to various transcription factor (TF) families were made into the different functional categories with respect to their putative functions. Phytohormone biosynthesis and signal transduction pathways were identified using *Arabidopsis* TF (http://plntfdb.bio.uni-potsdam.de/v3.0) and hormone (http://ahd.cbi.pku.edu.cn) databases, respectively (Fig. 3a; Additional files 3 and 4).

### Differentially expressed transcription factors

In the present study, DETs encoding TF belonging to various families were identified in the *Fl* line in fibreinitiation and elongation stages as compared to *FL* line. TFs belonging to AP2-EREBP, C2H2 and WRKY were highly up-regulated at fibre initiation (0 dpa). Similarly, transcripts encoding AP2-EREBP family TFs such as ethylene responsive element binding factors (ERFs), Integrase-type DNA-binding super family protein were highly down regulated at fibre elongation (10 dpa) stage in the *Fl* line. In addition, transcripts encoding salt tolerant zinc finger (STZ) TFs belonging to C2H2-type zinc finger family were down regulated at fibre initiation and elongation (Fig. 3a and Additional file 3). Further, transcripts (NAC047, NAC74, and NAC83) encoding NAC family were down regulated at 10 dpa and WRKY family belonging to DNA binding protein and protein kinase family protein were also down regulated at elongation stage.

Other transcripts encoding AUX/IAA (indole-3-acetic acid), MYB domain, bHLH (basic helix-loop-helix), PLATZ (PLATZ transcription factor family protein), Orphans (Signal transduction histidine kinase, hybrid-type, ethylene sensor), were down regulated at 10 dpa (Fig. 5 and Additional file 3). Controlling the expression level of the gene by transcription factors is complex phenomenon which includes its binding to specific genomic sequences and promotes, enhance, or block transcription. Among the several identified TFs controlling fibre initiation, many families of plant TFs are involved in the stress induced signaling cascade. The TFs mainly including AP2-EREBP, WRKY, NAC, MYB and bZIP have been proved to play vital roles in the regulation [17, 24, 25]. In present study, TFs belonging to the AP2-EREBP, C2H2, and WRKY were highly up-regulated at 0 dpa. Similarly, transcripts encoding AP2-EREBP family TFs such as ethylene responsive ERFs, Integrase-type DNA-binding super family protein were highly down regulated at 10 dpa in the *Fl* line. The AP2-EREBP transcription factor takes part in hormone signaling [26], spikelet meristem determinacy [27], leaf epidermal cell identity [28], embryo development [29], and regulation of flower development positively [26, 30]. Kurek et al. [31] reported that Zinc finger (C3HC4-type) and NAC family transcription factors influences the SCW synthesis in *Gossypium hirsutum* fibre. Zinc finger (C3HC4-type) family TFs also regulates cellulose synthesis via oxidation of zinc-binding domains [31]. In present study, up-regulation of Zinc finger (C3HC4-type) at initiation stage reveals the probable role in preventing the fibre initiation in microarray analysis by 16.50139 folds and same is validated by qRT-PCR which was showing up-regulation by 3.33 fold change (Table 2 and Fig. 5 Gene 1).

**Fig. 4:**
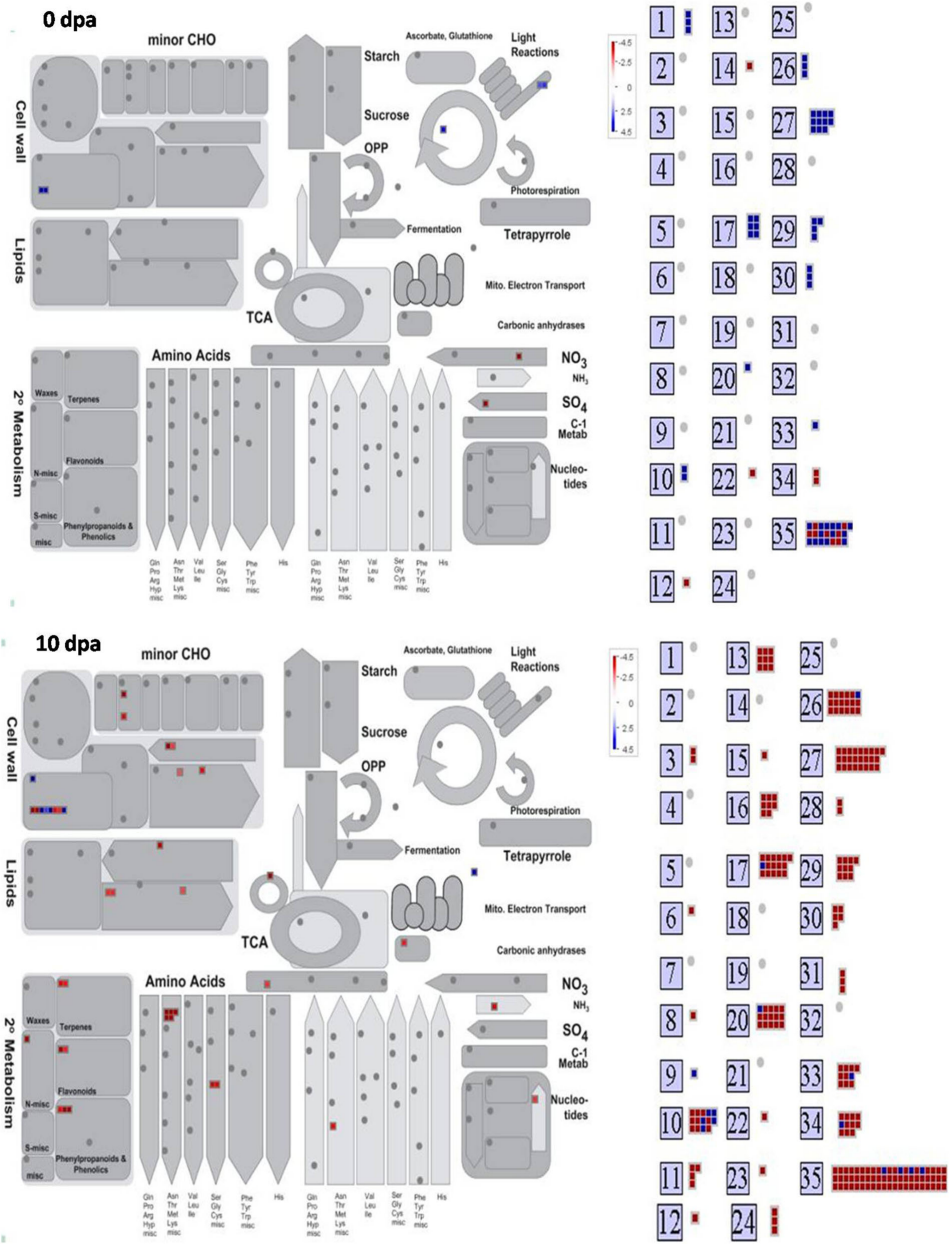
Overview of differentially expressed transcripts present in various metabolic processes based on MapMan (version 3.5) visualization software in *FL* line at 0 dpa and 10 dpa 1: PS (Photo System), 2: Major CHO metabolism, 3: Minor CHO metabolism, 4: Glycolysis, 5: Fermentation, 6: Gluconeogensis/glycoxylate cycle, 7: OPP (O-phenylphenol), 8: TCA (tricarboxylic acid)/Org. transformation, 9: Mitochondrial electron transport/ATP synthesis, 10: Cell wall, 11: Lipid metabolism, 12: N-metabolism, 13: Amino acid metabolism, 14: S-assimilation, 15: Amino acid metabolism, 16: S-assimilation, 17: Hormone metabolism, 18: Co-factor and vitamine metabolism, 19: Tetrapyrrole synthesis, 20: Stress, 21: Redox, 22: Polyamine metabolism, 23: Nucletotide metabolism, 24: Biodegradation of Xenobiotics, 25: C1-metabolism, 26: Misc27: RNA, 28: DNA, 29: Protein, 29: Protein, 30: Signalling, 31: Cell, 32: Micro RNA, Natural antisense etc., 33: Development, 34: Transport, 35: Not assigned

**Table 2:** Validation of DETs from microarray studies in *Gossypium arboreum* fuzzy-lintless (*Fl*) and fuzzy linted line (*FL*) through qRT-PCR

Sl. no.	Gene_ID	Stage	GenBank ID	Closest *Arabidopsis* homolog	Description	Fold change	Regulation	Log Fold Change to Cal. (ΔRn)	
								0dpa	10dpa
Gene 1	GhiAffx.7054.1.S1_at	0 dpa	DW509967.1	AT5G59550.1	zinc finger (C3HC4-type RING finger) family protein	16.50139	up	3.33	−1.67
Gene 2	GhiAffx.2527.1.S1_s_at	0 dpa	DW497370.1	AT5G53120.6	spermidine synthase 3	151.1868	down	−2.67	−2.41
Gene 3	Ghi.8931.1.S1_a_at	0 dpa	DT457712		Unknown	87.72372	down	−9.95	−16.8
Gene 4	Ghi.7853.1.S1_at	0 dpa	AF443118.1	AT1G01630.1	Sec14p-like phosphatidylinositol transfer family protein	5.828306	down	−8.39	−2.61
Gene 5	Ghi.7950.1.S1_at	10 dpa	AY366083.1	AT5G06720.1	peroxidase 2	81.1664	down	0.992	−18.8
Gene 6	GhiAffx.46297.1.S1_s_at	10 dpa	AI054544	AT4G17030.1	expansin-like B1	58.77188	down	−0.399	−8.24
Gene 7	Ghi.3370.1.A1_at	10 dpa	DT463939	AT1G33590.1	Leucine-rich repeat (LRR) family protein	45.29446	down	−1.52	−6.53
Gene 8	Ghi.8023.1.S1_at	10 dpa	DQ116443.1	AT1G12010.1	2-oxoglutarate (2OG) and Fe(II)-dependent oxygenase superfamily protein	39.72076	down	0.562	−6.11
Gene 10	GhiAffx.19125.1.A1_at	10 dpa	DW487672.1	AT1G12780.1	UDP-D-glucose/UDP-D-galactose 4-epimerase 1	19.17262	down	0.292	−3.89
Gene 11	Ghi.6496.1.S1_a_at	10 dpa	CD486227	AT4G02380.1	senescence-associated gene 21	18.63631	down	−2.12	−4.02
Gene 12	Ghi.1552.1.S1_s_at	10 dpa	DN779868	AT1G47960.1	cell wall/vacuolar inhibitor of fructosidase 1	18.20026	down	0.149	−4.94
Gene 13	Ghi.8448.1.S1_x_at	10 dpa	AF521240.1	AT5G12250.1	beta-6 tubulin	11.60727	down	1.58	−4.23
Gene 14	Ghi.6088.2.A1_s_at	10 dpa	DV849489	AT3G45640.1	mitogen-activated protein kinase 3	10.75481	down	−1.38	−4.58
Gene 15	Ghi.5521.1.A1_s_at	10 dpa	DT047436	AT5G01210.1	HXXXD-type acyl-transferase family protein	8.330377	down	−0.832	−2.08
Gene 16	Gra.2314.1.S1_at	10 dpa	CO126415	AT3G13750.1	beta galactosidase 1	6.476485	down	1.3	−2.58

### Phytohormones signaling

Phytohormones are one of the important factors play critical role through intracellular signaling events leading to well-characterised changes in gene expression for regulating various plant growth and developmental processes**.** In the present investigation, DETs involved in phytohormone biosynthesis and signal transduction pathways were identified at different stages in fuzzy-lintless *Fl* line as compared to their respective stages in fuzzy-linted line (*FL*). The genes involved in phytohormone signal transduction pathways and the biosynthesis of auxin, BR, ethylene, gibberellic acid (GA) and salicylic acid (SA) were found to be up-regulated at fibre initiation in the *Fl* line. But a single transcript encoding the jasmonic acid (JA) coding allene oxide synthase was found to be down regulated (Fig. 3b and Additional file 4). During the fibre elongation stages, transcripts which were found to be up-regulated at initiation were down regulated. Previous investigations reported that ethylene [18], auxin [16, 17], BR [16, 21], SA [16], GA [16], having additive effect on early fibre development and elongation.

High level of *iaaM* transcript was recorded in ovules of the transgenic lines transformed with epidermis cell specific promoter *FBP7* relative to wild type on the day of flowering (0 dpa) [16]. More number of fibre initials and elongating fibres at 0 to 3 dpa and significantly higher level of bioactive Gibberellic Acid (GA) in 0 dpa ovules and 10 dpa fibres due to constitutive over-expression of *GhGA20ox1* in cotton [32]. In the present investigation number of genes involved in phytohormone biosynthesis and signal transduction pathways were unraveled, changes in their expression level at different stages of the fiber development supports their role in fiber initiation and elongation. Sun et al. [33] collected the cotton ovules, on the day of anthesis and treated with brassinolide (BL) and brassinazole2001 (Brz) inhibitor. Fibre elongation was suppressed in the ovules treated with Brz while it was enormously increased in case of BL treatment, shows positive correlation between BR-regulated gene expression and fiber elongation [33].

### Different pathways expressed during the developmental stages

Mapman software version 3.5.0 (http://gabi.rzpd.de/projects/MapMan/) visualization shows overview of different metabolic pathways of the DETs involved in the *Fl* line at 0 dpa and 10 dpa (Additional files 5 and 6), respectively. Small blue and red colour squares indicate up- and down regulated transcripts, respectively (Fig. 4). This overview provides insight into specific functions, e.g. cell wall structure, metabolism, cell fate, signaling pathways etc. Some of the DETs are short listed (Additional files 7 and 8) which are present in various metabolic pathways in *Gossypium arboreum* fuzzy-lintless line (*Fl*) at 0 and 10 dpa.

### Energy and cell wall metabolism

During the initiation stage, there was no any expression/up-regulation of transcript encoding the carbohydrate (CHO) metabolism. But at the elongation stage, certain DETs encoding the minor CHO metabolism were down regulated. Transcripts encoding the enzymes involved in CHO metabolism and cell wall metabolism included Haloacid dehalogenase-like hydrolase (HAD) superfamily protein and also the enzymes involved in trehalose biosynthesis such as trehalose-phosphate phosphatase 9 (TPP) were down regulated at fibre elongation stage (Additional file 6). Recently, similar results have been found in the fuzzless-lintless mutant of *Gossypium hirsutum* L. cv.MCU5. Trehalose 6- phosphate synthase (TPS) were highly down regulated at fibre elongation stage indicating the role of these genes in fibre development and stress adaptation [8].

**Fig. 5:**
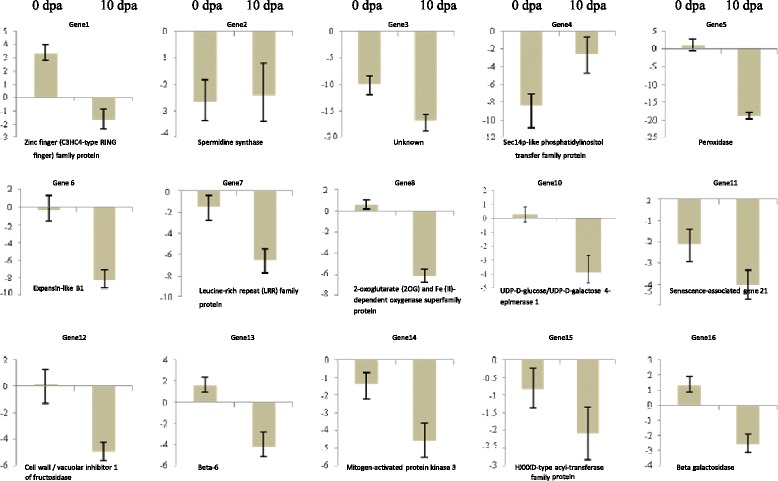
Validation of expression of genes obtained from microarray analysis in *Fl* line as compared to *FL* line at 0 dpa and 10 dpa

In fibre cells, cell wall biosynthesis is a major synthetic activity. Several transcripts take part in primary and secondary cell wall biosynthesis were differentially expressed at different stages of fibre development in fuzzy-lintless (*Fl*) line as compared to their respective stages in fuzzy-linted line (*FL*) (Fig. 4, Additional files 5 and 6). A number of genes involved in primary cell wall biosynthesis and elongation such as those coding for xyloglucanases, peptidoglycan-binding, expansins and glucose/galactose epimerase were down regulated at 10 dpa. UDP-D glucose (UDP-Glc) acts as a prime metabolite in carbohydrate metabolism, and also the precursor for the synthesis of cell wall polysaccharides such as pectin, hemicelluloses, and cellulose. In the present study, transcripts encoding the enzymes involved in synthesis of cell wall precursors, such as UDP-D-Glc/UDP-D-galactose 4-epimerase 1 were down regulated during fibre elongation stage in the *Fl* line confirmed by microarray analysis (19.17262 folds) and was also validated by qRT-PCR (3.89 folds) (Table 2 and Fig. 5, Gene 10). Some DETs coding for enzymes like UDP glucosyl and glucoronyl transferases family involving UDP-glucosyl transferase 74B1 (UGT74B1) and UDP-glucosyl transferase 74C1 (UGT74C1) and galactosidases (GAL) like β-galactosidase 1 and β-galactosidase 2 were highly down regulated at 10 dpa in the *Fl* line which is one of the convincing reasons for proving their role in fibre development.

In the present study at 0 dpa, transcripts encoding for cell wall modification like xyloglucan endotransglucosylase/hydrolase family protein (XTHs) and EXPANSINS were up-regulated. Transcripts encoding endo-xyloglucan hydrolase/XTH7 and endo-xyloglucan hydrolase/XTH32 involved in fibre elongation were highly up-regulated at 0 and 10 dpa that is the possible reason in the fuzz development. Fasciclin-like domain of AGPs, are important for cell-to-cell communication during cotton fibre elongation and secondary cell wall development [34] were also highly up-regulated. Further, transcripts encoding actin binding proteins such as profilin 5 (PRF5), actins (ACTs) which was actin depolymerizing factor 5, and β -tubulins coding for beta-6 tubulin were down regulated at 10 dpa involved in cell wall elongation and loosening, structural reinforcement and cytoskeleton dynamics indicating their role in fibre development. Pectin modifying enzymes such as pectin methylesterase inhibitor family protein coding for cell wall/vacuolar inhibitor of fructosidase 1 was down regulated during the elongation stage. Pectins are major components of the primary cell wall forms 25% of the cell wall constitution in cotton fibre. Thus, pectin modifying enzymes play a major role in the fibre cell wall development.

### Fatty acid (FA) metabolism

Fatty acid metabolism is responsible for the biosynthesis of many cellular lipids especially membrane components, hence rapid fatty acid synthesis in an elongating cotton fibre is expected. In the present study, no transcript encoding the enzymes involved in the biosynthesis of fatty acids was found at 0 dpa but seven acyl-activating enzymes involved in the FA synthesis and elongation were found to be down regulated in *Fl* line. The transcripts encoding alpha/beta-hydrolases protein which are essential for the lipid metabolism and lipid degradation were also found down regulated during the elongation phase. In addition, the factor acyl-CoA oxidase 4 essential for lipid metabolism and degradation involved in the beta-oxidation of the FA was also down regulated at 10 dpa (Additional file 6). Fatty acids (phosphoinositol and sphingolipids, and VLCFA-very-long-chain fatty acids) are the signaling molecules play crucial role in the fibre elongation. It was found that VLCFA accumulated more in elongating fibres as compared to ovules and the cotton genes involved in VLCFA biosynthesis (ketoacyl-coa synthase - KCS12, KCS6, KCS13, and KCS2) were up-regulated [35]. In the present study, there were hardly any transcript found encoding the enzymes involved in the biosynthesis of fatty acids at 0 dpa but transcripts involved in the FA synthesis at 10 dpa were found to be down regulated. The transcripts encoding alpha/beta-hydrolases protein which are essential for the lipid metabolism and lipid degradation were also found down regulated during the elongation phase. In addition, the factor acyl-CoA (ACO) oxidase 4 essential for lipid metabolism and degradation involved in the beta-oxidation of FA was down regulated at 10 dpa. Saturated VLCFAs may promote cotton fibre and *Arabidopsis* cell elongation by activating ethylene biosynthesis gene ACOs [35], pointing out an important link between the VLCFA biosynthesis and cotton fibre elongation. Studies in both *Arabidopsis* and cotton indicated that VLCFAs are involved in regulation of fibre elongation through their interplay with auxin and ethylene with subsequent stimulation of fibre cell elongation [35, 36].

LTPs are involved in transport of lipids from endoplasmic reticulum (ER) to plasma membrane (PM), where it acts as immediate acceptors of cuticular lipids from the plant ABCG transporters. Of course, this activation model would require either the shuttling of LTPs from the surface of the PM to cell wall [20, 37]. In the present study, bifunctional inhibitor/lipid-transfer protein also known seed storage 2S albumin super family protein and Sec14p-like phosphatidylinositol transfer family protein (PITP) were down regulated at 0 dpa in microarray by 5.828306 folds and also validated using qPCR showing its down-regulation by 8.39 folds which suggest the formation of fuzz in the *Fl* line (Table 2 and Fig. 5, Gene 4). Transcripts encoding the cell wall/vacuolar inhibitor of fructosidase-1 (C/VIF, cell wall/vacuolar inhibitor of fructosidase) having role in the carbohydrate metabolism and sugar signaling and related to pectin metabolism was found to be down regulated at 10 dpa (Additional file 6).

### Secondary metabolism

Secondary metabolism and modification of genes are amongst the most statistically significant differentially expressed categories during fibre elongation. In present study, not a single transcript was preferentially expressed at fibre initiation stage but during elongation stage, numbers of genes involved in secondary metabolism were down regulated. This included genes for many of enzymes of the isoprenoids, carotenoids and terpenoids coding beta ring hydroxylase 2 and terpene synthase 21 respectively. Gibberellin 3-oxidase 1 involved in synthesis of flavonols was also down regulated. Enzymes responsible for the synthesis of phenyl propanoid and lignin includes nicotinamidase 1, HXXXD-type acyl-transferase family protein, caffeoyl-CoA 3-O-methyltransferase were also down regulated. To date, only three genes of BAHD family of HXXXD-type acyltransferases which are mainly involved in alkyl hydroxyl cinnamate ester synthesis have been identified [38, 39]. Formation of feruloyloxy aliphatics (i.e. ferulate linked to the ω -terminus of ω-hydroxy fatty acids) in suberin and cutin polymers is the primary function of these genes. In *Arabidopsis thaliana*, alkyl ferulates comprise only a small proportion of the alkyl hydroxyl cinnamate esters extracted from roots [40]. Limited efforts on physiological parameters of roots that might be attributed to alkyl hydroxycinnamates and the general lack of information on mature *Arabidopsis* roots (transcriptome, stress experiments, etc.) provoked the researchers to induce the production of root alkyl hydroxycinnamate, alkyl coumarates and caffeates, that forms the components of *Arabidopsis thaliana* root waxes present primarily in taproots. Incorporation of ferulate into aliphatic suberin in *Arabidopsis* mediated by Aliphatic suberin feruloyl transferase (*At5g41040*) is a *HXXXD-type acyltransferase* (BAHD family). However, alkyl hydroxycinnamate ester root wax composition does not affect the aliphatic suberin feruloyl transferase mutants [41]*.* Here transcript coding HXXXD-type acyl-transferase was down regulated in microarray by 8.330377 and its validation suggesting down-regulation by 2.08 folds in *Fl* line which may lead to the formation of the secondary metabolites and reduced growth of the elongating fibres (Additional files 5 and 6; Table 2 and Fig. 5, Gene 15). Same kind of results has been reported by Tan et al., [42] that naringenin (NAR) a substrate of flavanone 3-hydroxylase (F3H) gene and silencing the F3H gene could significantly retard fiber development [42]. F3H mediated metabolism was evident from the results of Negative association of NAR with fiber development, thus indicating importance of flavonoid metabolism as a novel pathway with the potential for cotton fiber improvement.

### Transcripts related to signaling

Transcript coding signaling molecules such as calcium (Ca^2+^) and ROS specifically superoxide (O_2_^−^) and hydrogen peroxide (H_2_O_2_) have been studied recently for their role in cotton fibre development. In the present study, calcium signaling molecules associated with transcripts encoding Calcium-binding EF-hand family protein and ATPase E1-E2 type family protein/haloacid dehalogenase-like hydrolase family protein were up-regulated during initiation stage in *Fl* line as compared to *FL* line (Additional files 5 and 7). Transcripts involved in ROS including peroxidase superfamily Peroxidase 2 protein were down regulated at the elongation stage in this line (Additional files 6 and 8). These results showed the correlation with the previous studies indicates that ROS induced by exogenous H_2_O_2_ and Ca^2+^ starvation promotes early fiber elongation. Fiber cells show increased ROS concentrations compared with the wild-type due to *GhCaM7* overexpression, while *GhCaM7* RNAi fiber cells have reduced concentrations. Furthermore, H_2_O_2_ enhances Ca^2+^ influx into the fiber and intern-regulates the expression of *GhCaM7*. Increase in the cellular concentration of reactive oxygen species (ROS), subsequently converted to hydrogen peroxide (H_2_O_2_). Biotic and/or abiotic stress causes an oxidative burst creating disturbance in the cellular redox balance. This redox modulation could potentially alter protein conformation, affecting protein activity, and therefore initiating subsequent cellular responses is highly toxic to cells. In addition to being a toxicant, it has been considered as a signaling molecule and a regulator of the expression of some genes viz.*,* genes encoding antioxidants, cell rescue/defense proteins, and signaling proteins such as kinase, phosphatase, and transcription factors [43].

In cotton, ROS was detected through fluorescence of the ROS indicator 2′, 7′-dichlorodihydrofluoroscein diacetate (2, 7-DCH2FDA) in fiber initials at 0 dpa [44]. When bolls of *Gossypium hirsutum* fiber initiation mutants, naked seed (N1) and fuzzless Xinxianxiaoji (XinFLM), were treated with H_2_O_2_, fiber initials were expressed in both mutants by 0 dpa [45] indicating the role for ROS in fiber initiation. Similarly, research has begun to describe how Ca^2+^ participates in cotton fiber initiation and elongation [46] and it was observed that Ca^2+^ accumulation was correlated with fiber initiation and ER development in 0 dpa ovules compared to −1 dpa ovules, inline to this Microarray analysis showed that genes encoding components (calmodulin binding protein) of Ca^2+^ signaling were up-regulated at 1 dpa [25].

In plants, protein kinases and phosphatases play a key role in biotic and abiotic stress responses with concern in a wide range of developmental processes. In the present study several classes of protein kinases were found to be differentially expressed at MAPKs namely MPK3 and MPK9 were down regulated during 10 dpa. Similarly, receptor kinases like LLR transmembrane protein kinase and polygalacturonase inhibiting protein 1 belonging to LLR family were also shown down-regulation (Additional files 6 and 8).

### Other metabolisms

In the present study, down-regulation of certain transcripts like HSP20-like chaperones superfamily protein codes for HSPs, LRR family protein and senescence related genes/proteins coding for senescence-associated gene 21 which have been in association to the stresses. Transcript associated with the abiotic stress coding for Chaperone DnaJ-domain superfamily protein was found to be up-regulated at fibre initiation stage and the same was showing down-regulation at elongation stage. While studying the *Fl* line, spermidine protein encoding spermidine synthase was showing up-regulation at 0 dpa and down-regulation at 10 dpa (Additional files 5 and 6). All these factors support to abiotic stress resistance in plants. Therefore, their down-regulation arrested fibre elongation (as fibre elongation itself is a stressful process).

### Quantitative reverse transcription PCR

A total of 16 genes were selected for verification of the microarray data based on enrichment analysis of biological processes and expression profiles of genes differentially expressed during the initiation and elongation. (Table 2 and Fig. 5).

Several key gene function categories were selected which included cell wall biosynthesis and elongation factors including EXPANSIN-like B1, UDP-D-glucose/UDP-D-galactose 4-epimerase 1, tubulins, β-galactosidases, transcription factor like Zinc finger (C3HC4-type RING finger) family protein, MYB domains, LTPs like PITP, KCS genes involved in cellular communication and signal transduction encoding MAPK, MPKs.

For validation the cytoskeleton related genes having role in fibre elongation and onset of secondary cell wall deposition like actin depolymerizing factor (ADFs) were also selected. Genes like senescence-associated gene/SAG21 which responds to dehydration and signal transduction related genes like calcineurin b-like protein 01 (CBL1) involving in the calcium signaling. Some genes involved in carbohydrate metabolism, stress responses and sugar signaling like cell wall/vacuolar inhibitor of fructosidase 1 (AtC/VIF, cell wall/vacuolar inhibitor of fructosidase) and LRR proteins and Peroxidase 2 involving in ROS scavenging were also validated using qRT-PCR. The fold change regulation and the results are represented in the Table 2 and Fig. 5.

## Conclusion

Phenotypic characterization of fuzzy-lintless (*Fl*) line provides the information in elucidating mechanism regulating cotton fiber development and morphological difference between the lines. SEM analysis showed no difference in the fibre initials except for the fuzz development in fuzzy-lintless (*Fl*) line which is due to the down-regulation of some genes necessary for fibre development. Up-regulation of the transcription factors like AP2-EREBP, C2H2, C3H, HB, WRKY and phytohormones (auxin, ethylene, gibberlic acid and BR) biosynthesis at 0 dpa and their down-regulation at the 10 dpa might loss the co-ordination in the development process and ceased the fibre growth in fuzzy-lintless line. Likewise, genes involved in synthesis of VLCFA chain, singaling molecules in lipid metabolism (PtdIns) get down-regulated. Down-regulation of the transcripts necessary for the energy and cell wall metabolism such as TPS, endo-xyloglucan hydrolase family proteins, UDP-glucose 4-epimerase (UGE), EXPANSINs, AGPs and tubulin may be the probable reason for the undergrowth of the fibre in the *Fl* line. Transcript related to signaling i.e. Ca^2+^ and ROS as well as some HSPs and SPDS3 was down regulated mainly at 0 dpa which may lead to reduced fiber growth. Some miscellaneous factors like cellular and signal transduction coding for MAPK cascade, plant RLKs and LRR family proteins were down regulated at 10 dpa. Down-regulation of such of these factors known for cellular communication during fibre development resulted into fibrelessness in the mutant *Fl* line. This report also supports the previous findings of role of phytohormones in fibre development and LTPs in the transport of lipid molecule from plasma membrane to the cell wall.

## Additional files

Additional file 1: Table S1. Differentially expressed transcripts in *Gossypium arboreum* fuzzy-lintless line (*Fl*) as compared to fuzzy-linted line (*FL*) at fibre initiation stage (0 dpa). (DOC 93 kb)

Additional file 2: Table S2. Differentially expressed transcripts in *Gossypium arboreum* fuzzy-lintless line (*Fl*) as compared to fuzzy-linted line (*FL*) at fibre initiation stage (10 dpa). (DOC 284 kb)

Additional file 3: Table S3. Differentially expressed transcripts encoding transcription factors (TFs) in *Gossypium arboreum* fuzzy-lintless line (*Fl*) as compared to fuzzy-linted (*FL*) at fibre itiation stage (0 dpa) and fibre elongation stage (10 dpa). (DOC 75 kb)

Additional file 4: Table S4. Differentially expressed transcripts related to phytohormone signalling in *Gossypium arboreum* fuzzy-lintless line (*Fl*) as compared to fuzzy-linted (*FL*) at fibre at 0 dpa and 10 dpa. (DOC 156 kb)

Additional file 5: Table S5. Overview of differentially expressed transcripts present in various metabolic processes based on MapMan (version 3.5) visualization software *Gossypium arboreum* fuzzy-lintless line (*Fl*) at 0 dpa (DOC 103 kb)

Additional file 6: Table S6. Overview of differentially expressed transcripts present in various metabolic processes based on MapMan (version 3.5) visualization software in *Gossypium arboreum* fuzzy-lintless line (*Fl*) at 10 dpa. (DOC 313 kb)

Additional file 7: Table S7. Short listed view of differentially expressed transcripts present in various metabolic processes based on MapMan (version 3.5) visualization software in *Gossypium arboreum* fuzzy-lintless line (*Fl*) at 0 dpa. (DOC 37 kb)

Additional file 8: Table S8. Short listed view of differentially expressed transcripts present in various metabolic processes based on MapMan (version 3.5) visualization software in *Gossypium arboreum* fuzzy-lintless line (*Fl*) at 10 dpa. (DOC 93 kb)

## Abbreviations

2, 7-DCH2FDA: 2′, 7′-dichlorodihydrofluoroscein diacetate
ACO: Acyl-CoA
ACTs: Actins
ADFs: Actin depolymerizing factor
AGPs: Arabinogalactan proteins
BL: Brassinolide
BR: Brassinosteroids
CBL1: Calcineurin b-like protein 01
DETs: Differentially expressed transcripts
DPA: Days post anthesis
ER: Endoplasmic reticulum
ERFs: Element binding factors
F3H: Flavanone 3-hydroxylase
FDR: False discovery rate
GA: Gibberellic acid
GAL: Galactosidases
H_2_O_2_: Hydrogen peroxide
HAD: Haloacid dehalogenase-like hydrolase
HSPs: Heat shock proteins
JA: Jasmonic acid
LTPs: Lipid transfer proteins
NAR: Naringenin
PITP: Phosphatidylinositol transfer family protein
PM: Plasma membrane
RLKs: Plant receptor-like kinases
RMA: Robust Multiarray Average
ROS: Reactive oxygen species
SA: Salicylic acid
SCW: Secondary cell wall
SCWD: Secondary cell wall deposition
SEM: Scanning electron microscopy
SPDS3: Spermine synthase
STZ: Salt tolerant zinc finger
TF: Transcription factor
TPP: Trehalose-phosphate phosphatase 9
TPS: Trehalose 6- phosphate synthase
UDP-Glc: UDP-D glucose
UGT74B1: UDP-glucosyl transferase 74B1
UGT74C1: UDP-glucosyl transferase 74C1
VLCFA: Very-long-chain fatty acids
XTHs: Xyloglucan endotransglucosylase/hydrolase family protein
