# Supplementary material for: Transcript profiling of genes expressed during fibre development in diploid cotton (Gossypium arboreum L.)
Source: BMC Genomics. 2017 Aug 31;18:675. doi: 10.1186/s12864-017-4066-y (PMC5580217; doi:10.1186/s12864-017-4066-y)
Supplement: Supplementary file 1 — Differentially expressed transcripts in Gossypium arboreum fuzzy-lintless line (Fl) as compared to fuzzy-linted line (FL) at fibre initiation stage (0 dpa). (DOC 93 kb) [file 12864_2017_4066_MOESM1_ESM.doc]

**Table S1: Differentially expressed transcripts in *Gossypium arboreum* fuzzy-lintless line (*Fl*) as compared to fuzzy-linted line (*FL*) at fibre initiation stage (0 dpa).**

| **Sr. No** | **Gene_ID** | **Fold change** | **Regulation** | **UniGene ID** | **Accession No.** | **Closest Arabidopsis homolog** | **Description(TAIR database)** | ***E* value** |
| --- | --- | --- | --- | --- | --- | --- | --- | --- |
| 1 | GhiAffx.7054.1.S1_at | 16.50139 | Up | Ghi.9926 | DW509967.1 | AT5G59550.1 | zinc finger (C3HC4-type RING finger) family protein | 2.00E-41 |
| 2 | Ghi.10443.1.S1_at | 15.732822 | Up | Ghi.10443 | DT049130 | AT1G19210.1 | Integrase-type DNA-binding superfamily protein | 2.00E-39 |
| 3 | Ghi.1092.1.A1_x_at | 15.018939 | Up | Ghi.18632 | DT462950 | AT4G29780.1 | unknown protein | 2.00E-43 |
| 4 | Ghi.4920.1.A1_at | 14.513754 | Up | Ghi.4920 | DN758194 |  |  |  |
| 5 | Ghi.1092.3.S1_s_at | 12.998623 | Up | Ghi.18610 | DT467839 | AT4G29780.1 | unknown protein | 1.00E-103 |
| 6 | GhiAffx.11775.1.S1_at | 10.959008 | Up | Ghi.16170 | DW224036.1 | AT5G17350.1 | unknown protein | 4.00E-28 |
| 7 | GhiAffx.7649.1.S1_s_at | 10.767832 | Up | Ghi.18614 | DW478800.1 | AT5G12010.1 | unknown protein | 9.00E-72 |
| 8 | Ghi.3673.1.S1_at | 10.481772 | Up | Ghi.3673 | DT462887 | AT1G19210.1 | Integrase-type DNA-binding superfamily protein | 4.00E-44 |
| 9 | GhiAffx.52646.1.S1_at | 8.782509 | Up | Ghi.11801 | DW500254.1 | AT4G13395.1 | ROTUNDIFOLIA like 12 | 0.00000002 |
| 10 | Ghi.3284.1.S1_s_at | 7.9350553 | Up | Ghi.3284 | DT466688 | AT5G12010.1 | unknown protein | 3.00E-136 |
| 11 | GhiAffx.19697.1.A1_s_at | 7.9164195 | Up | Ghi.10366 | DW227913.1 | AT3G11760.1 | unknown protein | 4.00E-48 |
| 12 | Ghi.106.1.S1_s_at | 7.706848 | Up | Ghi.106 | DR462832 |  |  |  |
| 13 | Ghi.9175.1.S1_at | 7.629576 | Up | Ghi.9175 | DR462212 | AT5G51190.1 | Integrase-type DNA-binding superfamily protein | 2.00E-33 |
| 14 | Ghi.3763.1.A1_s_at | 7.1966577 | Up | Ghi.3763 | DT461952 | AT3G63380.1 | ATPase E1-E2 type family protein / haloacid dehalogenase-like hydrolase family protein | 2.00E-25 |
| 15 | Ghi.4983.1.A1_at | 6.4690266 | Up | Ghi.4983 | DV849718 | AT5G04340.1 | zinc finger of Arabidopsis thaliana 6 | 3.00E-32 |
| 16 | GhiAffx.24550.1.S1_at | 5.7237525 | Up | Ghi.16133 | DN818231 | AT4G27280.1 | Calcium-binding EF-hand family protein | 9.00E-34 |
| 17 | Ghi.664.1.S1_at | 5.093521 | Up | Ghi.664 | CA992783 | AT3G61460.1 | brassinosteroid-responsive RING-H2 | 1.00E-54 |
| 18 | Ghi.8610.1.S1_s_at | 5.069452 | Up | Ghi.8610 | CA992956 | AT5G22250.1 | Polynucleotidyl transferase, ribonuclease H-like superfamily protein | 1.00E-104 |
| 19 | Ghi.9192.1.S1_s_at | 4.986691 | Up | Ghi.9192 | DT468825 | AT1G80840.1 | WRKY DNA-binding protein 40 | 5.00E-50 |

| 20 | GhiAffx.63620.1.S1_at | 4.9628625 | Up | Ghi.15977 | DW505500.1 | AT5G55850.3 | RPM1-interacting protein 4 (RIN4) family protein | 7E-13 |
| --- | --- | --- | --- | --- | --- | --- | --- | --- |
| 21 | Ghi.10366.2.S1_at | 4.9414372 | Up | Ghi.10366 | AW561926 | AT2G25460.1 | CONTAINS InterPro DOMAIN/s: C2 calcium-dependent membrane targeting (InterPro:IPR000008) | 6.00E-50 |
| 22 | Ghi.8749.1.S1_at | 4.591602 | Up | Ghi.8749 | DT463517 | AT5G47230.1 | ethylene responsive element binding factor 5 | 2.00E-45 |
| 23 | Ghi.10493.1.S1_s_at | 4.426241 | up | Ghi.17551 | DT466412 | AT5G57560.1 | Xyloglucan endotransglucosylase/hydrolase family protein | 2.00E-62 |
| 24 | GhiAffx.59715.1.S1_at | 4.221705 | up | Ghi.16596 | DW505344.1 | AT1G19210.1 | Integrase-type DNA-binding superfamily protein | 4.00E-30 |
| 25 | GhiAffx.21219.1.A1_s_at | 4.078241 | up | Ghi.9948 | DW236303.1 | AT1G69530.2 | expansin A1 | 7.00E-44 |
| 26 | Ghi.1085.2.S1_at | 3.879253 | up | Ghi.4 | CD485897 | AT3G26770.1 | NAD(P)-binding Rossmann-fold superfamily protein | 5.00E-43 |
| 27 | Ghi.5451.1.S1_at | 3.8172166 | up | Ghi.16253 | DQ122174.1 | AT4G11280.1 | 1-aminocyclopropane-1-carboxylic acid (acc) synthase 6 | 2.00E-176 |
| 28 | GhiAffx.5925.1.S1_at | 3.7370589 | up | Ghi.12812 | DW502097.1 | AT2G45760.1 | BON association protein 2 | 2.00E-26 |
| 29 | GhiAffx.3411.1.A1_at | 3.6744685 | up | Ghi.11868 | DW497356.1 | AT2G28710.1 | C2H2-type zinc finger family protein | 1.00E-26 |
| 30 | GbaAffx.201.1.S1_s_at | 3.6479692 | up |  | AY560553.1 |  |  |  |
| 31 | Ghi.2608.2.A1_at | 3.6166418 | up | Ghi.2608 | DT463212 | AT3G55840.1 | Hs1pro-1 protein | 3.00E-28 |
| 32 | Ghi.1513.1.S1_x_at | 3.6074338 | up | Ghi.1513 | CA994331 | AT5G38430.1 | Ribulose bisphosphate carboxylase (small chain) family protein | 2.00E-77 |
| 33 | Ghi.9149.4.A1_s_at | 3.5930614 | up | Ghi.17231 | CA993608 | AT1G32920.1 | unknown protein | 0.000004 |
| 34 | Ghi.1016.4.S1_s_at | 3.5463805 | up | Ghi.10821 | DT468576 | AT3G09270.1 | glutathione S-transferase TAU 8 | 2.00E-28 |
| 35 | Ghi.8126.1.S1_x_at | 3.5254004 | up | Ghi.6690 | AY779339.1 | AT3G15210.1 | ethylene responsive element binding factor 4 | 2.00E-16 |
| 36 | GhiAffx.5954.1.S1_s_at | 3.5167634 | up | Ghi.9346 | DW225147.1 | AT3G16720.1 | TOXICOS EN LEVADURA 2 | 8.00E-47 |
| 37 | Ghi.6748.1.A1_at | 3.456143 | up | Ghi.6748 | CA993446 | AT3G57450.1 | unknown protein | 6.00E-17 |
| 38 | GhiAffx.1119.1.S1_at | 3.3052182 | up | Ghi.13018 | DW242880.1 |  |  |  |
| 39 | GhiAffx.1119.2.S1_at | 3.2936802 | up | Ghi.15519 | DW225844.1 | ATCG00720.1 | photosynthetic electron transfer B | 2.00E-116 |

| 40 | Ghi.10778.2.S1_at | 3.2575119 | up | Ghi.10778 | CA993737 | AT2G17880.1 | Chaperone DnaJ-domain superfamily protein | 9.00E-27 |
| --- | --- | --- | --- | --- | --- | --- | --- | --- |
| 41 | GhiAffx.61657.1.S1_at | 3.1391716 | up | Ghi.12559 | DW510592.1 | AT5G17220.1 | glutathione S-transferase phi 12 | 2.00E-66 |
| 42 | Ghi.9880.2.A1_x_at | 3.1105497 | up | Ghi.9880 | DT047349 | AT5G47230.1 | ethylene responsive element binding factor 5 | 4.00E-16 |
| 43 | Ghi.760.1.A1_x_at | 3.0644503 | up | Ghi.760 | DN827346 | AT1G52820.1 | 2-oxoglutarate (2OG) and Fe(II)-dependent oxygenase superfamily protein | 6.00E-43 |
| 44 | Ghi.807.1.S1_s_at | 3.046491 | up | Ghi.17797 | DT465871 | AT1G27730.1 | salt tolerance zinc finger | 5.00E-30 |
| 45 | Ghi.10635.1.S1_x_at | 3.0207536 | up | Ghi.6780 | DN760160 | AT2G34430.1 | light-harvesting chlorophyll-protein complex II subunit B1 | 3.00E-127 |
| 46 | Ghi.3784.2.S1_s_at | 3.000441 | up | Ghi.3784 | DT457597 | AT5G19120.1 | Eukaryotic aspartyl protease family protein | 1.00E-17 |
| 47 | GhiAffx.2527.1.S1_s_at | 151.18683 | down | Ghi.13939 | DW497370.1 | AT5G53120.6 | spermidine synthase 3 | 3.00E-60 |
| 48 | Ghi.8931.1.S1_a_at | 87.72372 | down | Ghi.8931 | DT457712 |  |  |  |
| 49 | GhiAffx.22562.1.A1_at | 28.217451 | down | Ghi.13380 | DW238476.1 | AT2G04420.1 | Polynucleotidyl transferase, ribonuclease H-like superfamily protein | 0.000000002 |
| 50 | Ghi.7853.1.S1_at | 5.8283057 | down | Ghi.16284 | AF443118.1 | AT1G01630.1 | Sec14p-like phosphatidylinositol transfer family protein | 3.00E-74 |
| 51 | Ghi.5852.3.S1_s_at | 5.147951 | down | Ghi.5852 | CO498687 | AT3G01910.3 | sulfite oxidase | 1.00E-27 |
| 52 | GhiAffx.10850.1.A1_s_at | 4.545873 | down | Ghi.15954 | DN799961 | AT5G62680.1 | Major facilitator superfamily protein | 8.00E-17 |
| 53 | GhiAffx.25239.1.S1_at | 3.7861333 | down | Ghi.15924 | DW515297.1 |  |  |  |
| 54 | Ghi.5304.2.A1_x_at | 3.652858 | down | Ghi.5304 | DT048257 | AT5G42650.1 | allene oxide synthase | 0.000000001 |
| 55 | Ghi.5587.1.S1_at | 3.6226969 | down | Ghi.5587 | CA993040 |  |  |  |
| 56 | Ghi.1319.1.S1_s_at | 3.0949988 | down | Ghi.16399 | AF118230.1 | AT3G15353.1 | metallothionein 3 | 0.0000003 |
| 57 | GhiAffx.4679.1.A1_a_at | 3.0866187 | down | Ghi.12168 | DW510621.1 | AT4G01935.1 | unknown protein | 6.00E-73 |
| 58 | Ghi.884.1.S1_at | 3.0236187 | down | Ghi.884 | DR453639 | AT5G66985.1 | unknown protein | 0.000003 |
