# Supplementary material for: Transcript profiling of genes expressed during fibre development in diploid cotton (Gossypium arboreum L.)
Source: BMC Genomics. 2017 Aug 31;18:675. doi: 10.1186/s12864-017-4066-y (PMC5580217; doi:10.1186/s12864-017-4066-y)
Supplement: Supplementary file 2 — Differentially expressed transcripts in Gossypium arboreum fuzzy-lintless line (Fl) as compared to fuzzy-linted line (FL) at fibre initiation stage (10 dpa). (DOC 284 kb) [file 12864_2017_4066_MOESM2_ESM.doc]

**Table S2: Differentially expressed transcripts in *Gossypium arboreum* fuzzy-lintless line (*Fl*) as compared to fuzzy-linted line (*FL*) at fibre initiation stage (10 dpa).**

| **Sr. No** | **Gene_ID** | **Fold change** | **Regulation** | **UniGene ID** | **Accession No.** | **Closest Arabidopsis homolog** | **Description(TAIR database)** | ***E* value** |
| --- | --- | --- | --- | --- | --- | --- | --- | --- |
| 1 | Ghi.5860.1.S1_s_at | 46.18646 | up | Ghi.16418 | CO494172 | AT2G04780.2 | FASCICLIN-like arabinoogalactan 7 | 3.00E-41 |
| 2 | GhiAffx.32614.1.A1_x_at | 20.26679 | up |  | CO498615 |  |  |  |
| 3 | GhiAffx.49772.1.A1_at | 9.510919 | up |  | DT464025 | AT5G01890.1 | Leucine-rich receptor-like protein kinase family protein | 2E-15 |
| 4 | Ghi.1812.1.S1_at | 8.004698 | up | Ghi.1812 | DV850410 |  |  |  |
| 5 | Ghi.6236.2.S1_s_at | 7.36092 | up | Ghi.6236 | D88413.1 | AT4G37800.1 | xyloglucan endotransglucosylase/hydrolase 7 | 6.00E-134 |
| 6 | GraAffx.8958.1.S1_s_at | 6.785116 | up |  | CO087973 | AT4G37800.1 | xyloglucan endotransglucosylase/hydrolase 7 | 4.00E-111 |
| 7 | Ghi.6236.1.S1_s_at | 5.846538 | up | Ghi.6236 | AY189971.1 | AT4G37800.1 | xyloglucan endotransglucosylase/hydrolase 7 | 3.00E-124 |
| 8 | GhiAffx.52777.1.S1_at | 4.350262 | up | Ghi.15921 | DW515277.1 | ATCG00480.1 | ATP synthase subunit beta | 1.00E-138 |
| 9 | GhiAffx.42790.1.S1_at | 3.87759 | up |  | DW509815.1 | AT3G01550.1 | phosphoenolpyruvate (pep)/phosphate translocator 2 | 1.00E-64 |
| 10 | Ghi.272.1.S1_s_at | 3.845166 | up | Ghi.272 | DT561048 | AT1G64080.1 | unknown protein | 7.00E-24 |
| 11 | Ghi.10795.1.S1_s_at | 3.265911 | up | Ghi.6472 | CD486563 | AT2G45180.1 | Bifunctional inhibitor/lipid-transfer protein/seed storage 2S albumin superfamily protein | 1.00E-18 |
| 12 | GhiAffx.6062.1.S1_at | 3.16694 | up | Ghi.15960 | DW495992.1 | AT5G20820.1 | SAUR-like auxin-responsive protein family | 2.00E-21 |
| 13 | GraAffx.27319.1.S1_s_at | 3.155124 | up |  | CO089724 | AT2G36870.1 | xyloglucan endotransglucosylase/hydrolase 32 | 7.00E-143 |
| 14 | Gra.2669.1.A1_at | 3.080133 | up | Gra.2669 | CO085938 | AT1G70260.1 | nodulin MtN21 /EamA-like transporter family protein | 6.00E-26 |
| 15 | Ghi.6472.1.A1_s_at | 3.066469 | up | Ghi.6472 | CD485893 | AT2G45180.1 | Bifunctional inhibitor/lipid-transfer protein/seed storage 2S albumin superfamily protein | 5E-14 |
| 16 | GhiAffx.2527.1.S1_s_at | 168.9751 | down | Ghi.13939 | DW497370.1 | AT5G53120.6 | spermidine synthase 3 | 3.00E-60 |
| 17 | Ghi.7950.1.S1_at | 81.1664 | down | Ghi.16267 | AY366083.1 | AT5G06720.1 | peroxidase 2 | 5.00E-94 |

| 18 | GhiAffx.3185.1.S1_at | 76.70687 | down | Ghi.17263 | DW514553.1 | AT1G78860.1 | D-mannose binding lectin protein with Apple-like carbohydrate-binding domain | 2.00E-112 |
| --- | --- | --- | --- | --- | --- | --- | --- | --- |
| 19 | GhiAffx.46297.1.S1_s_at | 58.77188 | down | Ghi.6465 | AI054544 | AT4G17030.1 | expansin-like B1 | 5E-14 |
| 20 | Ghi.8931.1.S1_a_at | 57.431 | down | Ghi.8931 | DT457712 |  |  |  |
| 21 | Ghi.6465.2.S1_at | 55.04907 | down | Ghi.6465 | CD485906 | AT4G17030.1 | expansin-like B1 | 2.00E-30 |
| 22 | Ghi.3370.1.A1_at | 45.29446 | down | Ghi.3370 | DT463939 | AT1G33590.1 | Leucine-rich repeat (LRR) family protein | 2.00E-78 |
| 23 | Gra.2141.1.S1_s_at | 39.88526 | down |  | CO123471 | AT1G05010.1 | ethylene-forming enzyme | 3.00E-118 |
| 24 | Ghi.8023.1.S1_at | 39.72076 | down | Ghi.16693 | DQ116443.1 | AT1G12010.1 | 2-oxoglutarate (2OG) and Fe(II)-dependent oxygenase superfamily protein | 4.00E-126 |
| 25 | Gra.1559.1.A1_at | 22.42489 | down | Gra.2231 | CO123242 | AT3G47340.1 | glutamine-dependent asparagine synthase 1 | 8.00E-85 |
| 26 | Gra.2231.1.S1_s_at | 21.86533 | down | Gra.2231 | CO089843 | AT3G47340.1 | glutamine-dependent asparagine synthase 1 | 0 |
| 27 | GhiAffx.8053.1.A1_at | 21.1322 | down |  | DW518810.1 | AT1G64160.1 | Disease resistance-responsive (dirigent-like protein) family protein | 4.00E-49 |
| 28 | Ghi.6067.1.A1_at | 20.80368 | down | Ghi.6067 | DT048703 | AT3G22600.1 | Bifunctional inhibitor/lipid-transfer protein/seed storage 2S albumin superfamily protein | 0.00000003 |
| 29 | Ghi.1660.1.S1_s_at | 19.49105 | down | Ghi.1660 | DN760124 |  |  |  |
| 30 | GhiAffx.19125.1.A1_at | 19.17262 | down |  | DW487672.1 | AT1G12780.1 | UDP-D-glucose/UDP-D-galactose 4-epimerase 1 | 7.00E-139 |
| 31 | Ghi.6496.1.S1_a_at | 18.63631 | down | Ghi.8364 | CD486227 | AT4G02380.1 | senescence-associated gene 21 | 1.00E-16 |
| 32 | Ghi.1552.1.S1_s_at | 18.20026 | down | Ghi.1552 | DN779868 | AT1G47960.1 | cell wall / vacuolar inhibitor of fructosidase 1 | 2.00E-25 |
| 33 | GhiAffx.7806.1.S1_at | 17.08208 | down | Ghi.1230 | DW515985.1 | AT1G80130.1 | Tetratricopeptide repeat (TPR)-like superfamily protein | 3.00E-32 |
| 34 | Ghi.9146.1.S1_s_at | 15.87656 | down | Ghi.9146 | DT463593 | AT5G13740.1 | zinc induced facilitator 1 | 1.00E-52 |
| 35 | Ghi.6780.1.S1_s_at | 15.1656 | down | Ghi.6780 | CA993199 | AT2G17880.1 | Chaperone DnaJ-domain superfamily protein | 4.00E-24 |
| 36 | Ghi.8364.1.A1_at | 15.02456 | down | Ghi.8364 | CA993541 | AT4G02380.1 | senescence-associated gene 21 | 2.00E-16 |
| 37 | Ghi.6953.1.S1_s_at | 15.0156 | down | Ghi.16374 | DQ116442.1 | AT1G05010.1 | ethylene-forming enzyme | 7.00E-121 |
| 38 | Ghi.490.1.S1_s_at | 14.92188 | down | Ghi.490 | DT465033 | AT1G33590.1 | Leucine-rich repeat (LRR) family protein | 2.00E-25 |

| 39 | Ghi.6538.1.S1_at | 14.71982 | down | Ghi.6538 | CD485949 | AT3G04070.1 | NAC domain containing protein 47 | 1.00E-84 |
| --- | --- | --- | --- | --- | --- | --- | --- | --- |
| 40 | Ghi.9152.2.A1_at | 14.70235 | down | Ghi.17824 | DT463871 |  |  |  |
| 41 | GhiAffx.44018.1.S1_at | 12.24216 | down | Ghi.15557 | DW502867.1 | AT1G30260.1 | BEST Arabidopsis thaliana protein match is: Galactosyltransferase family protein (TAIR:AT4G21060.1) | 0.000000001 |
| 42 | Ghi.9193.2.A1_at | 12.23719 | down | Ghi.9193 | DT469110 | AT3G56400.1 | WRKY DNA-binding protein 70 | 4.00E-26 |
| 43 | Ghi.8448.1.S1_x_at | 11.60727 | down | Ghi.8448 | AF521240.1 | AT5G12250.1 | beta-6 tubulin | 0 |
| 44 | Ghi.6693.1.A1_at | 11.06929 | down | Ghi.6693 | CA993655 |  |  |  |
| 45 | GraAffx.1410.1.S1_at | 11.0522 | down |  | CO125821 | AT5G65980.1 | Auxin efflux carrier family protein | 9.00E-102 |
| 46 | Ghi.699.1.S1_s_at | 10.82811 | down | Ghi.699 | DR456003 |  |  |  |
| 47 | Ghi.6088.2.A1_s_at | 10.75481 | down | Ghi.6088 | DV849489 | AT3G45640.1 | mitogen-activated protein kinase 3 | 3.00E-27 |
| 48 | Ghi.10655.1.S1_s_at | 10.4581 | down | Ghi.10655 | DN780602 | AT3G47340.1 | glutamine-dependent asparagine synthase 1 | 0 |
| 49 | Ghi.3578.1.S1_s_at | 10.34841 | down | Ghi.3578 | DT567472 | AT4G36740.1 | homeobox protein 40 | 2.00E-44 |
| 50 | Ghi.9152.1.S1_at | 10.33359 | down | Ghi.9152 | DT462541 | AT3G18950.1 | Transducin/WD40 repeat-like superfamily protein | 2.00E-99 |
| 51 | Ghi.7874.1.S1_s_at | 10.27751 | down | Ghi.16277 /// Ghi.7874 | AY962572.1 | AT3G16770.1 | ethylene-responsive element binding protein | 1.00E-34 |
| 52 | Ghi.8389.1.S1_a_at | 10.24861 | down | Ghi.8389 | CO498953 | AT4G15610.1 | Uncharacterised protein family (UPF0497) | 1.00E-22 |
| 53 | Ghi.6362.1.S1_at | 10.04844 | down | Ghi.6362 | CO491551 |  |  |  |
| 54 | Ghi.7907.2.A1_at | 9.67557 | down | Ghi.7907 | DT463334 |  |  |  |
| 55 | Ghi.7942.2.S1_a_at | 9.429709 | down | Ghi.7942 | DT467978 | AT2G21620.1 | Adenine nucleotide alpha hydrolases-like superfamily protein | 2.00E-66 |
| 56 | Ghi.8123.1.S1_at | 8.612653 | down | Ghi.8123 | X52305.1 | AT5G03860.2 | malate synthase | 0 |
| 57 | Ghi.6543.1.S1_s_at | 8.538899 | down | Ghi.6543 | DN780646 | AT4G14550.1 | indole-3-acetic acid inducible 14 | 4.00E-77 |
| 58 | GhiAffx.31391.1.S1_s_at | 8.354315 | down |  | DW481920.1 | AT3G18830.1 | polyol/monosaccharide transporter 5 | 9.00E-122 |
| 59 | Ghi.5521.1.A1_s_at | 8.330377 | down | Ghi.5521 | DT047436 | AT5G01210.1 | HXXXD-type acyl-transferase family protein | 2.00E-17 |
| 60 | Ghi.8033.1.S1_s_at | 8.29359 | down | Ghi.16611 | DQ122187.1 | AT2G20340.1 | Pyridoxal phosphate (PLP)-dependent transferases superfamily protein | 2.00E-155 |
| 61 | Ghi.1043.4.S1_at | 8.142763 | down | Ghi.17859 | DT463348 | AT5G05340.1 | Peroxidase superfamily protein | 2.00E-66 |

| 62 | Ghi.5887.1.S1_at | 7.941222 | down | Ghi.5887 | CO499594 | AT5G54940.2 | Translation initiation factor SUI1 family protein | 3.00E-46 |
| --- | --- | --- | --- | --- | --- | --- | --- | --- |
| 63 | Ghi.1016.4.S1_s_at | 7.797561 | down | Ghi.10821 | DT468576 | AT3G09270.1 | glutathione S-transferase TAU 8 | 2.00E-28 |
| 64 | Ghi.8381.1.S1_s_at | 7.79501 | down | Ghi.8381 | DT047422 |  |  |  |
| 65 | Ghi.3335.1.A1_at | 7.793686 | down | Ghi.19561 | DT463623 |  |  |  |
| 66 | GhiAffx.53241.1.S1_at | 7.749757 | down | Ghi.12870 | DW518118.1 | AT1G58170.1 | Disease resistance-responsive (dirigent-like protein) family protein | 1.00E-54 |
| 67 | Ghi.7171.1.A1_at | 7.735075 | down | Ghi.7171 | DT461933 |  |  |  |
| 68 | Ghi.8400.1.S1_s_at | 7.734738 | down | Ghi.9142 | DN827419 | AT2G15130.1 | Plant basic secretory protein (BSP) family protein | 8.00E-18 |
| 69 | Gra.1614.2.S1_x_at | 7.722575 | down | Gra.3247 | CO127533 | AT3G47340.1 | glutamine-dependent 4sparagines synthase 1 | 3.00E-179 |
| 70 | GhiAffx.22562.1.A1_at | 7.689648 | down | Ghi.13380 | DW238476.1 | AT2G04420.1 | Polynucleotidyl transferase, ribonuclease H-like superfamily protein | 0.000000002 |
| 71 | Ghi.6632.1.A1_s_at | 7.671162 | down | Ghi.2362 | DT461730 | AT1G07150.2 | mitogen-activated protein kinase kinase kinase 13 | 2E-14 |
| 72 | GbaAffx.196.1.A1_s_at | 7.633128 | down |  | AY572462.1 | AT3G16770.1 | ethylene-responsive element binding protein | 5.00E-29 |
| 73 | Ghi.4.1.A1_at | 7.606558 | down | Ghi.4 | CK987701 | AT1G52340.1 | NAD(P)-binding Rossmann-fold superfamily protein | 7.00E-64 |
| 74 | Ghi.1935.1.S1_at | 7.599018 | down | Ghi.1935 | DV848993 | AT3G29590.1 | HXXXD-type acyl-transferase family protein | 9.00E-26 |
| 75 | Ghi.3264.2.A1_s_at | 7.568856 | down | Ghi.3264 | DT463678 |  |  |  |
| 76 | Ghi.1436.1.S1_at | 7.506804 | down | Ghi.10655 | DN799898 | AT3G47340.1 | glutamine-dependent 4sparagines synthase 1 | 2.00E-24 |
| 77 | GhiAffx.33994.1.A1_s_at | 7.453239 | down |  | DW515886.1 | AT3G04070.2 | NAC domain containing protein 47 | 0.00000005 |
| 78 | Ghi.9243.2.A1_s_at | 7.434102 | down | Ghi.9243 | DR452409 | AT4G17900.1 | PLATZ transcription factor family protein | 3.00E-90 |
| 79 | Ghi.6822.1.A1_s_at | 7.313375 | down | Ghi.6822 | CA993006 | AT2G36750.1 | UDP-glucosyl transferase 73C1 | 1.00E-53 |
| 80 | Ghi.1847.1.S1_at | 7.26273 | down | Ghi.1847 | DV850045 | AT5G46050.1 | peptide transporter 3 | 2.00E-63 |
| 81 | GhiAffx.1859.1.S1_at | 7.239213 | down |  | DT468306 | AT5G13080.1 | WRKY DNA-binding protein 75 | 1.00E-40 |
| 82 | GhiAffx.5935.2.S1_s_at | 7.227638 | down | Ghi.9213 | DW235907.1 | AT3G55840.1 | Hs1pro-1 protein | 1.00E-50 |
| 83 | Ghi.8105.1.A1_s_at | 7.123931 | down | Ghi.8105 | AF488305.1 | AT5G06720.1 | peroxidase 2 | 9.00E-103 |

| 84 | Ghi.3135.1.S1_at | 7.021998 | down | Ghi.3135 | DT469074 | AT1G32450.1 | nitrate transporter 1.5 | 3.00E-127 |
| --- | --- | --- | --- | --- | --- | --- | --- | --- |
| 85 | Ghi.6449.1.A1_at | 7.005597 | down | Ghi.6449 | CK640602 |  |  |  |
| 86 | Ghi.9146.2.S1_s_at | 6.696793 | down | Ghi.24492 | DT463838 | AT5G13740.1 | zinc induced facilitator 1 | 5.00E-62 |
| 87 | Ghi.5307.1.A1_at | 6.609644 | down | Ghi.5307 | DT048232 | AT3G48140.1 | B12D protein | 2.00E-32 |
| 88 | Ghi.4737.1.A1_at | 6.521595 | down | Ghi.4737 | DT051082 | AT1G26820.1 | ribonuclease 3 | 4.00E-68 |
| 89 | Gra.2314.1.S1_at | 6.476485 | down | Gra.2314 | CO126415 | AT3G13750.1 | beta galactosidase 1 | 4.00E-124 |
| 90 | Ghi.6088.1.S1_s_at | 6.336911 | down | Ghi.6088 | DT466983 | AT3G45640.1 | mitogen-activated protein kinase 3 | 2.00E-136 |
| 91 | Ghi.7279.1.S1_at | 6.333603 | down | Ghi.7279 | DR455241 | AT2G26910.1 | pleiotropic drug resistance 4 | 1.00E-23 |
| 92 | Ghi.7891.1.S1_s_at | 6.325849 | down | Ghi.7891 | DT462224 | AT1G03220.1 | Eukaryotic aspartyl protease family protein | 2.00E-86 |
| 93 | GhiAffx.18573.1.S1_s_at | 6.32572 | down | Ghi.1313 | DW500069.1 | AT1G15400.3 | unknown protein | 4.00E-23 |
| 94 | GhiAffx.22064.1.S1_at | 6.317846 | down | Ghi.12850 | DW498676.1 | AT3G14680.1 | cytochrome P450, family 72, subfamily A, polypeptide 14 | 6.00E-94 |
| 95 | GhiAffx.9239.1.S1_s_at | 6.295898 | down | Ghi.12614 | DW244026.1 | AT2G35940.3 | BEL1-like homeodomain 1 | 4E-13 |
| 96 | Ghi.5997.1.A1_at | 6.278993 | down | Ghi.5997 | CO492935 | AT1G29050.1 | TRICHOME BIREFRINGENCE-LIKE 38 | 4.00E-29 |
| 97 | Ghi.7907.1.S1_s_at | 6.206703 | down | Ghi.7907 | AI055500 | AT4G27410.2 | NAC (No Apical Meristem) domain transcriptional regulator superfamily protein | 2.00E-84 |
| 98 | Gra.2459.1.A1_s_at | 6.184939 | down | Gra.3587 | CO128080 | AT3G52840.1 | beta-galactosidase 2 | 2.00E-19 |
| 99 | Ghi.341.1.S1_x_at | 6.176137 | down | Ghi.341 | DR460630 |  |  |  |
| 100 | GhiAffx.48583.1.S1_at | 6.144695 | down |  | AI055122 | AT3G06490.1 | myb domain protein 108 | 3.00E-68 |
| 101 | Ghi.6539.1.S1_s_at | 6.073285 | down | Ghi.9213 | CD485942 | AT2G40000.1 | ortholog of sugar beet HS1 PRO-1 2 | 8.00E-33 |
| 102 | Ghi.1621.1.S1_x_at | 5.960155 | down | Ghi.1621 | DN760810 | AT3G27880.1 | Protein of unknown function (DUF1645) | 4E-13 |
| 103 | Ghi.6417.3.S1_s_at | 5.899771 | down | Ghi.6417 | DN826313 |  |  |  |
| 104 | Ghi.5022.4.A1_s_at | 5.890732 | down | Ghi.17602 | DT049392 | AT1G73500.1 | MAP kinase kinase 9 | 2.00E-38 |
| 105 | Ghi.2608.2.A1_at | 5.85847 | down | Ghi.2608 | DT463212 | AT3G55840.1 | Hs1pro-1 protein | 3.00E-28 |
| 106 | Ghi.3264.1.S1_s_at | 5.772931 | down | Ghi.3264 | DT466083 | AT1G01720.1 | NAC (No Apical Meristem) domain transcriptional regulator superfamily protein | 2.00E-125 |
| 107 | Ghi.4532.1.A1_at | 5.760909 | down | Ghi.4532 | DR457588 | AT4G18990.1 | xyloglucan endotransglucosylase/hydrolase 29 | 2.00E-16 |
| 108 | Ghi.6901.1.A1_s_at | 5.749808 | down | Ghi.6901 | CA992707 | AT1G27730.1 | salt tolerance zinc finger | 9E-11 |

| 109 | Ghi.4960.2.A1_s_at | 5.676947 | down | Ghi.4960 | DT046397 | AT4G27740.1 | Yippee family putative zinc-binding protein | 4.00E-17 |
| --- | --- | --- | --- | --- | --- | --- | --- | --- |
| 110 | Ghi.4960.1.A1_s_at | 5.639016 | down | Ghi.4960 | DT051751 | AT4G27740.1 | Yippee family putative zinc-binding protein | 5.00E-26 |
| 111 | Ghi.10753.1.S1_at | 5.614182 | down | Ghi.10753 | DN760125 | AT4G27450.1 | Aluminium induced protein with YGL and LRDR motifs | 9.00E-84 |
| 112 | GhiAffx.25472.1.A1_s_at | 5.611794 | down |  | DW516477.1 | AT3G04720.1 | pathogenesis-related 4 | 4.00E-75 |
| 113 | Gra.1544.1.A1_s_at | 5.587249 | down |  | CO091149 | AT3G63010.1 | alpha/beta-Hydrolases superfamily protein | 7.00E-144 |
| 114 | Ghi.5775.1.S1_s_at | 5.514077 | down | Ghi.5775 | DT455881 | AT3G23150.1 | Signal transduction histidine kinase, hybrid-type, ethylene sensor | 4.00E-44 |
| 115 | Ghi.6417.4.S1_s_at | 5.504288 | down | Ghi.6417 | CO490783 |  |  |  |
| 116 | Ghi.1326.1.S1_s_at | 5.472375 | down | Ghi.1326 | DR463721 | AT3G25710.1 | basic helix-loop-helix 32 | 9.00E-26 |
| 117 | Ghi.3446.1.A1_at | 5.419879 | down | Ghi.3446 | DT462755 | AT4G28530.1 | NAC domain containing protein 74 | 2.00E-16 |
| 118 | Ghi.1955.1.S1_at | 5.386776 | down | Ghi.1955 | DV848730 | AT4G31240.2 | protein kinase C-like zinc finger protein | 7.00E-47 |
| 119 | Ghi.6722.1.S1_s_at | 5.302019 | down | Ghi.16396 | M88322.1 | AT1G01470.1 | Late embryogenesis abundant protein | 4.00E-59 |
| 120 | Ghi.9328.1.S1_s_at | 5.254816 | down | Ghi.4821 | DT048550 | AT5G13180.1 | NAC domain containing protein 83 | 2.00E-79 |
| 121 | GhiAffx.19570.1.S1_at | 5.245414 | down | Ghi.14271 | DW489734.1 | AT2G26560.1 | phospholipase A 2A | 7.00E-65 |
| 122 | Ghi.967.2.S1_s_at | 5.179194 | down | Ghi.967 | DR452676 | AT4G00230.1 | xylem serine peptidase 1 | 4E-13 |
| 123 | GhiAffx.25661.1.S1_at | 5.122869 | down | Ghi.11202 | DW517516.1 | AT3G27880.1 | Protein of unknown function (DUF1645) | 1.00E-24 |
| 124 | Gra.2056.1.A1_s_at | 5.045487 | down |  | CO121156 | AT2G16730.1 | glycosyl hydrolase family 35 protein | 2.00E-61 |
| 125 | Ghi.4349.1.A1_at | 5.01652 | down | Ghi.4349 | DT054063 | AT1G67980.2 | caffeoyl-CoA 3-O-methyltransferase | 1E-15 |
| 126 | Ghi.8110.1.S1_at | 4.90623 | down | Ghi.8110 | AY311597.1 | AT5G05340.1 | Peroxidase superfamily protein | 2.00E-132 |
| 127 | Ghi.3480.1.S1_at | 4.864676 | down | Ghi.3480 | DN760204 |  |  |  |
| 128 | GbaAffx.201.1.S1_s_at | 4.838012 | down |  | AY560553.1 |  |  |  |
| 129 | Ghi.3370.1.S1_s_at | 4.830588 | down | Ghi.3370 | DT466783 | AT1G33590.1 | Leucine-rich repeat (LRR) family protein | 2.00E-78 |
| 130 | Ghi.8424.1.A1_at | 4.822119 | down | Ghi.8424 | CO490790 |  |  |  |
| 131 | Ghi.10747.1.S1_at | 4.822069 | down | Ghi.10747 | DV850132 | AT4G17500.1 | ethylene responsive element binding factor 1 | 1.00E-52 |
| 132 | GhiAffx.23896.1.S1_at | 4.721586 | down | Ghi.11550 | DW497980.1 | AT4G21510.1 | F-box family protein | 1.00E-32 |
| 133 | GhiAffx.59002.1.S1_at | 4.708658 | down | Ghi.14714 | DW497255.1 | AT1G19530.1 | unknown protein | 7.00E-18 |

| 134 | Ghi.912.3.A1_at | 4.706751 | down | Ghi.912 | DT468062 | AT5G65140.1 | Haloacid dehalogenase-like hydrolase (HAD) superfamily protein | 8.00E-83 |
| --- | --- | --- | --- | --- | --- | --- | --- | --- |
| 135 | GhiAffx.2060.1.S1_at | 4.663229 | down |  | DW477541.1 | AT1G15210.1 | pleiotropic drug resistance 7 | 8.00E-125 |
| 136 | Ghi.6523.1.S1_s_at | 4.638748 | down | Ghi.6523 | AF305064.1 |  |  |  |
| 137 | Ghi.1037.2.S1_x_at | 4.610213 | down | Ghi.8296 | CA993067 | AT3G15353.2 | metallothionein 3 | 0.0000007 |
| 138 | GhiAffx.7865.1.S1_at | 4.594069 | down |  | DW503266.1 | AT5G61590.1 | Integrase-type DNA-binding superfamily protein | 6.00E-34 |
| 139 | GhiAffx.16191.1.S1_at | 4.588662 | down | Ghi.11788 | DW507100.1 | AT2G29500.1 | HSP20-like chaperones superfamily protein | 2.00E-32 |
| 140 | Ghi.5587.1.S1_at | 4.540395 | down | Ghi.5587 | CA993040 |  |  |  |
| 141 | Ghi.5512.1.A1_s_at | 4.50005 | down | Ghi.5512 | DT047390 |  |  |  |
| 142 | Ghi.5626.1.S1_s_at | 4.467655 | down | Ghi.5626 | DT466989 | AT4G01500.1 | AP2/B3-like transcriptional factor family protein | 0.0000001 |
| 143 | Ghi.6410.1.S1_at | 4.438521 | down | Ghi.6410 | CD486551 | AT1G70830.3 | MLP-like protein 28 | 4.00E-49 |
| 144 | GhiAffx.21558.1.S1_s_at | 4.398123 | down |  | DW495821.1 | AT1G11190.1 | bifunctional nuclease I | 4.00E-117 |
| 145 | Ghi.967.1.S1_s_at | 4.380848 | down | Ghi.967 | AI728289 | AT5G67090.1 | Subtilisin-like serine endopeptidase family protein | 3.00E-83 |
| 146 | GhiAffx.42158.1.S1_at | 4.374403 | down | Ghi.18276 | DW233814.1 |  |  |  |
| 147 | Ghi.10676.1.S1_s_at | 4.287772 | down | Ghi.10676 | DT567365 | AT1G01360.1 | regulatory component of ABA receptor 1 | 1.00E-76 |
| 148 | Ghi.3184.1.S1_s_at | 4.250644 | down | Ghi.1202 | DT468147 | AT3G16910.1 | acyl-activating enzyme 7 | 6.00E-142 |
| 149 | Gra.2150.1.S1_s_at | 4.206167 | down | Gra.2150 | CO085918 | AT5G06570.2 | alpha/beta-Hydrolases superfamily protein | 1.00E-40 |
| 150 | GbaAffx.197.1.S1_s_at | 4.198405 | down |  | AY279356.1 | AT5G06860.1 | polygalacturonase inhibiting protein 1 | 1.00E-122 |
| 151 | Ghi.592.1.S1_at | 4.195285 | down | Ghi.592 | DR457234 |  |  |  |
| 152 | Ghi.1586.1.S1_at | 4.195183 | down | Ghi.17925 | DN760004 | AT2G36950.1 | Heavy metal transport/detoxification superfamily protein | 1.00E-43 |
| 153 | Ghi.798.1.S1_s_at | 4.159749 | down | Ghi.16386 | DQ116444.1 | AT1G05010.1 | ethylene-forming enzyme | 2.00E-130 |
| 154 | GbaAffx.207.1.S1_s_at | 4.109701 | down |  | AY560551.1 |  |  |  |
| 155 | Ghi.812.1.S1_at | 4.103185 | down | Ghi.812 | DT462925 | AT5G60680.1 | Protein of unknown function, DUF584 | 5.00E-33 |
| 156 | GhiAffx.28739.1.S1_s_at | 4.040947 | down | Ghi.15180 | DW502086.1 | AT5G47220.1 | ethylene responsive element binding factor 2 | 2.00E-29 |
| 157 | GhiAffx.15571.1.S1_a_at | 4.016547 | down |  | DW505599.1 | AT1G67920.1 | unknown protein | 0.00000001 |
| 158 | GarAffx.19282.1.S1_s_at | 3.983656 | down |  | AF416652.1 | AT1G24020.2 | MLP-like protein 423 | 0.0000006 |

| 159 | Ghi.6500.1.S1_at | 3.980963 | down | Ghi.6500 | AI728753 | AT3G03910.1 | glutamate dehydrogenase 3 | 2.00E-167 |
| --- | --- | --- | --- | --- | --- | --- | --- | --- |
| 160 | GhiAffx.38714.1.S1_at | 3.948362 | down | Ghi.11105 | DW492836.1 | AT3G19990.1 | unknown protein | 5.00E-110 |
| 161 | GhiAffx.31355.1.S1_s_at | 3.936843 | down | Ghi.13637 | DT462536 | AT4G34138.1 | UDP-glucosyl transferase 73B1 | 1.00E-84 |
| 162 | Ghi.8342.1.S1_at | 3.905866 | down | Ghi.8342 | DV849242 |  |  |  |
| 163 | Gra.911.1.A1_s_at | 3.903166 | down | Gra.911 | CO121286 | AT3G61440.1 | BSAS3 | 3.00E-156 |
| 164 | GhiAffx.10920.2.S1_at | 3.875228 | down | Ghi.16091 | DW237583.1 | AT2G37170.1 | 2 | 9.00E-80 |
| 165 | Ghi.468.1.A1_s_at | 3.868022 | down | Ghi.16435 | AY189970.1 | AT2G19770.1 | profilin 5 | 7.00E-59 |
| 166 | Ghi.807.1.S1_s_at | 3.851732 | down | Ghi.17797 | DT465871 | AT1G27730.1 | salt tolerance zinc finger | 5.00E-30 |
| 167 | Ghi.6485.1.S1_s_at | 3.829199 | down |  | CD485617 |  |  |  |
| 168 | Ghi.5401.1.S1_s_at | 3.822087 | down | Ghi.5401 | DT047881 | AT3G61440.1 | BSAS3 | 5.00E-134 |
| 169 | Ghi.3451.2.A1_at | 3.805538 | down | Ghi.3451 | DT465672 | AT1G23870.1 | trehalose-phosphatase/synthase 9 | 4.00E-35 |
| 170 | Ghi.1533.1.S1_s_at | 3.79672 | down | Ghi.1533 | DR463368 |  |  |  |
| 171 | GhiAffx.9522.1.A1_s_at | 3.781433 | down | Ghi.19273 | DT049461 | AT3G25030.2 | RING/U-box superfamily protein | 4.00E-23 |
| 172 | GhiAffx.7814.1.S1_s_at | 3.70053 | down | Ghi.21981 | DW516033.1 | AT1G49320.1 | unknown seed protein like 1 | 1.00E-51 |
| 173 | GhiAffx.41174.1.A1_at | 3.689941 | down | Ghi.1905 | DW501616.1 |  |  |  |
| 174 | Ghi.4725.1.S1_s_at | 3.686573 | down | Ghi.4725 | AI726805 | AT5G13870.1 | xyloglucan endotransglucosylase/hydrolase 5 | 2.00E-98 |
| 175 | GhiAffx.17151.1.S1_at | 3.686039 | down | Ghi.19974 | DW243562.1 | AT5G52570.1 | beta-carotene hydroxylase 2 | 3.00E-86 |
| 176 | GraAffx.14609.1.A1_s_at | 3.66268 | down |  | CO127185 | AT4G33150.3 | lysine-ketoglutarate reductase/saccharopine dehydrogenase bifunctional enzyme | 3.00E-48 |
| 177 | GhiAffx.30199.1.S1_at | 3.661612 | down | Ghi.15715 | DW506814.1 | AT2G47260.1 | WRKY DNA-binding protein 23 | 7.00E-45 |
| 178 | GhiAffx.15490.1.S1_at | 3.632752 | down | Ghi.18655 | DW237402.1 | AT2G22570.1 | nicotinamidase 1 | 7.00E-88 |
| 179 | GhiAffx.61299.1.S1_at | 3.61332 | down | Ghi.18695 | DW508705.1 | AT1G17860.1 | Kunitz family trypsin and protease inhibitor protein | 1.00E-33 |
| 180 | Ghi.6102.1.S1_at | 3.575107 | down | Ghi.6102 | DR458734 |  |  |  |
| 181 | Ghi.6548.1.S1_s_at | 3.550099 | down | Ghi.16406 | AY207316.1 | AT5G55560.1 | Protein kinase superfamily protein | 5.00E-123 |
| 182 | Ghi.6547.1.S1_s_at | 3.532587 | down |  | DR458804 | AT4G12320.1 | cytochrome P450, family 706, subfamily A, polypeptide 6 | 6.00E-103 |
| 183 | GarAffx.37202.1.S1_x_at | 3.502486 | down |  | U23205.1 | AT5G23960.2 | terpene synthase 21 | 3.00E-98 |
| 184 | GhiAffx.1082.1.S1_at | 3.497402 | down | Ghi.12989 | DW511203.1 | AT4G33580.1 | beta carbonic anhydrase 5 | 3.00E-74 |
| 185 | Ghi.1037.4.A1_x_at | 3.494166 | down | Ghi.8296 | CA993008 |  |  |  |
| 186 | GhiAffx.1402.1.S1_at | 3.436156 | down | Ghi.14499 | DW231451.1 | AT1G15550.1 | gibberellin 3-oxidase 1 | 1.00E-73 |

| 187 | GraAffx.17697.1.A1_s_at | 3.434198 | down |  | CO086224 | AT5G47560.1 | tonoplast dicarboxylate transporter | 6.00E-70 |
| --- | --- | --- | --- | --- | --- | --- | --- | --- |
| 188 | Ghi.9176.3.A1_at | 3.419338 | down | Ghi.9176 | DT464586 | AT3G51680.1 | NAD(P)-binding Rossmann-fold superfamily protein | 1.00E-57 |
| 189 | Ghi.3049.4.S1_at | 3.416076 | down | Ghi.3049 | DT463685 | AT5G02230.2 | Haloacid dehalogenase-like hydrolase (HAD) superfamily protein | 8.00E-54 |
| 190 | Gra.377.1.A1_s_at | 3.413284 | down | Gra.2265 | CO085937 | AT2G06850.1 | xyloglucan endotransglucosylase/hydrolase 4 | 1.00E-58 |
| 191 | Ghi.7293.1.S1_s_at | 3.39789 | down | Ghi.7293 | AI731856 | AT2G39420.1 | alpha/beta-Hydrolases superfamily protein | 4.00E-92 |
| 192 | GarAffx.19282.1.S1_x_at | 3.390929 | down |  | AF416652.1 | AT1G24020.2 | MLP-like protein 423 | 0.0000006 |
| 193 | Ghi.6262.1.S1_x_at | 3.36667 | down |  | DT464008 | AT4G17900.1 | PLATZ transcription factor family protein | 2.00E-48 |
| 194 | GhiAffx.24021.1.S1_at | 3.3666 | down |  | DW509098.1 | AT2G44970.2 | alpha/beta-Hydrolases superfamily protein | 4.00E-83 |
| 195 | Gra.998.2.S1_s_at | 3.354782 | down | Gra.998 | CO089323 | AT2G27830.1 | unknown protein | 2.00E-34 |
| 196 | Ghi.44.1.A1_at | 3.345396 | down | Ghi.44 | DR463380 | AT5G10290.1 | leucine-rich repeat transmembrane protein kinase family protein | 6.00E-44 |
| 197 | GhiAffx.36084.1.A1_at | 3.337532 | down | Ghi.21982 | DW516195.1 | AT2G04240.2 | RING/U-box superfamily protein | 2.00E-45 |
| 198 | GhiAffx.61605.1.S1_s_at | 3.336749 | down | Ghi.16587 | DN800946 | AT3G62550.1 | Adenine nucleotide alpha hydrolases-like superfamily protein | 9.00E-46 |
| 199 | Ghi.7536.1.S1_s_at | 3.306124 | down | Ghi.7536 | AI729592 | AT2G02010.1 | glutamate decarboxylase 4 | 0 |
| 200 | Gra.2847.1.A1_s_at | 3.293587 | down | Gra.2847 | CO071816 | AT3G17810.1 | pyrimidine 1 | 0 |
| 201 | Ghi.6557.1.S1_at | 3.270496 | down | Ghi.6557 | CD485783 | AT2G01300.1 | unknown protein | 4.00E-21 |
| 202 | Ghi.5305.1.A1_at | 3.252828 | down | Ghi.5305 | DT048241 | AT5G25890.1 | indole-3-acetic acid inducible 28 | 0.000003 |
| 203 | GhiAffx.60835.1.S1_at | 3.234412 | down | Ghi.12975 | DW506319.1 | AT5G58380.1 | SOS3-interacting protein 1 | 4.00E-61 |
| 204 | Gra.2050.1.S1_s_at | 3.231735 | down | Gra.2050 | CO126131 | AT1G12780.1 | UDP-D-glucose/UDP-D-galactose 4-epimerase 1 | 2.00E-170 |
| 205 | Gra.275.1.S1_s_at | 3.212169 | down | Gra.275 | CO090219 | AT1G60710.1 | NAD(P)-linked oxidoreductase superfamily protein | 1.00E-143 |
| 206 | GhiAffx.30941.1.S1_s_at | 3.207843 | down | Ghi.13511 | DW482613.1 | AT1G13260.1 | related to ABI3/VP1 1 | 4.00E-68 |
| 207 | GhiAffx.429.1.A1_at | 3.186557 | down |  | DW233845.1 | AT4G37890.2 | Zinc finger (C3HC4-type RING finger) family protein | 1.00E-56 |
| 208 | Ghi.7466.1.S1_at | 3.141613 | down | Ghi.7466 | AI730393 | AT3G51840.1 | acyl-CoA oxidase 4 | 3.00E-81 |

| 209 | Gra.378.1.A1_s_at | 3.13665 | down | Gra.378 | CO085861 | AT3G53980.2 | Bifunctional inhibitor/lipid-transfer protein/seed storage 2S albumin superfamily protein | 4.00E-40 |
| --- | --- | --- | --- | --- | --- | --- | --- | --- |
| 210 | GhiAffx.6395.1.S1_s_at | 3.122005 | down | Ghi.13987 | DW484802.1 | AT4G14550.1 | indole-3-acetic acid inducible 14 | 2.00E-82 |
| 211 | Ghi.9281.1.A1_s_at | 3.119268 | down | Ghi.17961 | DT047152 | AT4G36740.1 | homeobox protein 40 | 8.00E-50 |
| 212 | Ghi.6692.1.S1_s_at | 3.078492 | down | Ghi.6692 | CA993159 | AT1G67920.1 | unknown protein | 2.00E-16 |
| 213 | GhiAffx.28716.1.S1_at | 3.064089 | down | Ghi.15689 | DW512426.1 | AT4G00880.1 | SAUR-like auxin-responsive protein family | 8.00E-29 |
| 214 | Gra.2844.1.S1_s_at | 3.043563 | down | Gra.17 | CO087535 | AT5G50790.1 | Nodulin MtN3 family protein | 4.00E-78 |
| 215 | GhiAffx.26021.1.S1_at | 3.036362 | down | Ghi.16198 | DW232275.1 | AT1G15380.2 | Lactoylglutathione lyase / glyoxalase I family protein | 1.00E-43 |
| 216 | GhiAffx.60253.1.S1_at | 3.027403 | down | Ghi.14666 | DW503386.1 | AT5G62150.1 | peptidoglycan-binding LysM domain-containing protein | 1.00E-31 |
| 217 | Ghi.8524.1.S1_at | 3.021253 | down | Ghi.18592 | DT048651 | AT5G49360.1 | beta-xylosidase 1 | 0 |
| 218 | Ghi.779.1.S1_at | 3.019226 | down | Ghi.779 | DN800052 | AT1G10370.1 | Glutathione S-transferase family protein | 5.00E-61 |
| 219 | GhiAffx.33535.1.S1_at | 3.014881 | down |  | AI727184 | AT2G16700.2 | actin depolymerizing factor 5 | 6.00E-58 |
| 220 | Ghi.3049.1.A1_at | 3.001209 | down | Ghi.3049 | DR456375 | AT5G02230.2 | Haloacid dehalogenase-like hydrolase (HAD) superfamily protein | 3.00E-33 |
