# Supplementary material for: Transcript profiling of genes expressed during fibre development in diploid cotton (Gossypium arboreum L.)
Source: BMC Genomics. 2017 Aug 31;18:675. doi: 10.1186/s12864-017-4066-y (PMC5580217; doi:10.1186/s12864-017-4066-y)
Supplement: Supplementary file 3 — Differentially expressed transcripts encoding transcription factors (TFs) in Gossypium arboreum fuzzy-lintless line (Fl) as compared to fuzzy-linted (FL) at fibre itiation stage (0 dpa) and fibre elongation stage (10 dpa). (DOC 75 kb) [file 12864_2017_4066_MOESM3_ESM.doc]

**Table S3: Differentially expressed transcripts encoding transcription factors (TFs) in *Gossypium arboreum* fuzzy-lintless line (*Fl*) as compared to fuzzy-linted (*FL*) at fibre itiation stage (0 dpa) and fibre elongation stage (10).**

| **TF – Family** | **Gene_ID** | **Fold change** | **Regulation** | **UniGene ID** | **GenBank ID** | **Closest Arabidopsis homolog** | **Description(TAIR database)** | ***E* value** |
| --- | --- | --- | --- | --- | --- | --- | --- | --- |
| **Fibre initiation stage (0 dpa)** | | | | | | | | |
| **AP2-EREBP** | Ghi.10443.1.S1_at | 15.732822 | up | Ghi.10443 | DT049130 | AT1G19210.1 | Integrase-type DNA-binding superfamily protein | 2.00E-40 |
|  | Ghi.3673.1.S1_at | 10.481772 | up | Ghi.3673 | DT462887 | AT1G19210.1 | Integrase-type DNA-binding superfamily protein | 4.00E-45 |
|  | Ghi.9175.1.S1_at | 7.629576 | up | Ghi.9175 | DR462212 | AT5G51190.1 | Integrase-type DNA-binding superfamily protein | 1.00E-34 |
|  | Ghi.8749.1.S1_at | 4.591602 | up | Ghi.8749 | DT463517 | AT5G47230.1 | ethylene responsive element binding factor 5 | 2.00E-46 |
|  | GhiAffx.59715.1.S1_at | 4.221705 | up | Ghi.16596 | DW505344.1 | AT1G19210.1 | Integrase-type DNA-binding superfamily protein | 3.00E-31 |
|  | Ghi.8126.1.S1_x_at | 3.5254004 | up | Ghi.6690 | AY779339.1 | AT3G15210.1 | ethylene responsive element binding factor 4 | 1.00E-17 |
|  | Ghi.9880.2.A1_x_at | 3.1105497 | up | Ghi.9880 | DT047349 | AT5G47230.1 | ethylene responsive element binding factor 5 | 4.00E-17 |
| **C2H2** | Ghi.4983.1.A1_at | 6.4690266 | up | Ghi.4983 | DV849718 | AT5G04340.1 | zinc finger of Arabidopsis thaliana 6 | 3.00E-33 |
|  | GhiAffx.3411.1.A1_at | 3.6744685 | up | Ghi.11868 | DW497356.1 | AT2G28710.1 | C2H2-type zinc finger family protein | 9.00E-28 |
|  | Ghi.807.1.S1_s_at | 3.046491 | up | Ghi.17797 | DT465871 | AT1G27730.1 | salt tolerance zinc finger | 4.00E-31 |
| **WRKY** | Ghi.9192.1.S1_s_at | 4.986691 | up | Ghi.9192 | DT468825 | AT1G80840.1 | WRKY DNA-binding protein 40 | 4.00E-51 |
| **Fibre elongation stage (10 dpa)** | | | | | | | | |
| **AP2-EREBP** | Ghi.7874.1.S1_s_at | 10.277513 | down | Ghi.16277 /// Ghi.7874 | AY962572.1 | AT3G16770.1 | ethylene-responsive element binding protein | 1.00E-35 |
|  | GbaAffx.196.1.A1_s_at | 7.6331277 | down |  | AY572462.1 | AT3G16770.1 | ethylene-responsive element binding protein | 5.00E-30 |
|  | Ghi.10747.1.S1_at | 4.822069 | down | Ghi.10747 | DV850132 | AT5G47220.1 | ethylene responsive element binding factor 2 | 1.00E-44 |
|  | GhiAffx.7865.1.S1_at | 4.5940685 | down |  | DW503266.1 | AT5G61590.1 | Integrase-type DNA-binding superfamily protein | 5.00E-35 |
|  | GhiAffx.28739.1.S1_s_at | 4.0409465 | down | Ghi.15180 | DW502086.1 | AT5G47220.1 | ethylene responsive element binding factor 2 | 2.00E-30 |
|  | GhiAffx.30941.1.S1_s_at | 3.2078433 | down | Ghi.13511 | DW482613.1 | AT1G13260.1 | related to ABI3/VP1 1 | 3.00E-69 |
| **AUX/IAA** | Ghi.6543.1.S1_s_at | 8.538899 | down | Ghi.6543 | DN780646 | AT4G14550.1 | indole-3-acetic acid inducible 14 | 3.00E-78 |
|  | GhiAffx.6395.1.S1_s_at | 3.1220052 | down | Ghi.13987 | DW484802.1 | AT4G14550.1 | indole-3-acetic acid inducible 14 | 2.00E-83 |
| **bHLH** | Ghi.1326.1.S1_s_at | 5.472375 | down | Ghi.1326 | DR463721 | AT3G25710.1 | basic helix-loop-helix 32 | 8.00E-27 |
| **C2H2** | Ghi.6901.1.A1_s_at | 5.749808 | down | Ghi.6901 | CA992707 | AT1G27730.1 | salt tolerance zinc finger | 9E-12 |
|  | Ghi.807.1.S1_s_at | 3.851732 | down | Ghi.17797 | DT465871 | AT1G27730.1 | salt tolerance zinc finger | 4.00E-31 |

| **C3H** | Ghi.9152.1.S1_at | 10.333591 | down | Ghi.9152 | DT462541 | AT5G49200.1 | WD-40 repeat family protein / zfwd4 protein (ZFWD4) | 3.00E-16 |
| --- | --- | --- | --- | --- | --- | --- | --- | --- |
| **HB** | Ghi.3578.1.S1_s_at | 10.348414 | down | Ghi.3578 | DT567472 | AT4G36740.1 | homeobox protein 40 | 2.00E-45 |
|  | GhiAffx.9239.1.S1_s_at | 6.2958975 | down | Ghi.12614 | DW244026.1 | AT2G35940.3 | BEL1-like homeodomain 1 | 3E-14 |
|  | Ghi.9281.1.A1_s_at | 3.1192684 | down | Ghi.17961 | DT047152 | AT4G36740.1 | homeobox protein 40 | 7.00E-51 |
| **MYB** | GhiAffx.48583.1.S1_at | 6.144695 | down |  | AI055122 | AT3G06490.1 | myb domain protein 108 | 3.00E-69 |
| **NAC** | Ghi.6538.1.S1_at | 14.719816 | down | Ghi.6538 | CD485949 | AT3G04070.1 | NAC domain containing protein 47 | 1.00E-85 |
|  | Ghi.7907.1.S1_s_at | 6.206703 | down | Ghi.7907 | AI055500 | AT4G27410.2 | NAC (No Apical Meristem) domain transcriptional regulator superfamily protein | 2.00E-85 |
|  | Ghi.3264.1.S1_s_at | 5.772931 | down | Ghi.3264 | DT466083 | AT1G01720.1 | NAC (No Apical Meristem) domain transcriptional regulator superfamily protein | 2.00E-126 |
|  | Ghi.3446.1.A1_at | 5.4198785 | down | Ghi.3446 | DT462755 | AT4G28530.1 | NAC domain containing protein 74 | 2.00E-17 |
|  | Ghi.9328.1.S1_s_at | 5.254816 | down | Ghi.4821 | DT048550 | AT5G13180.1 | NAC domain containing protein 83 | 2.00E-80 |
| **Orphans** | Ghi.5775.1.S1_s_at | 5.514077 | down | Ghi.5775 | DT455881 | AT3G23150.1 | Signal transduction histidine kinase, hybrid-type, ethylene sensor | 4.00E-45 |
| **PLATZ** | Ghi.9243.2.A1_s_at | 7.4341016 | down | Ghi.9243 | DR452409 | AT4G17900.1 | PLATZ transcription factor family protein | 3.00E-91 |
|  | Ghi.6262.1.S1_x_at | 3.3666704 | down |  | DT464008 | AT4G17900.1 | PLATZ transcription factor family protein | 2.00E-49 |
| **WRKY** | Ghi.9193.2.A1_at | 12.23719 | down | Ghi.9193 | DT469110 | AT3G56400.1 | WRKY DNA-binding protein 70 | 4.00E-27 |
|  | GhiAffx.1859.1.S1_at | 7.2392125 | down |  | DT468306 | AT5G13080.1 | WRKY DNA-binding protein 75 | 1.00E-41 |
|  | Ghi.6088.1.S1_s_at | 6.3369107 | down | Ghi.6088 | DT466983 | AT4G12020.2 | protein kinase family protein | 5.00E-18 |
|  | Ghi.5022.4.A1_s_at | 5.890732 | down | Ghi.17602 | DT049392 | AT4G12020.2 | protein kinase family protein | 3E-10 |
|  | GhiAffx.30199.1.S1_at | 3.6616116 | down | Ghi.15715 | DW506814.1 | AT2G47260.1 | WRKY DNA-binding protein 23 | 6.00E-46 |
|  | Ghi.6548.1.S1_s_at | 3.5500994 | down | Ghi.16406 | AY207316.1 | AT4G12020.2 | protein kinase family protein | 5.00E-21 |
