# Supplementary material for: Transcript profiling of genes expressed during fibre development in diploid cotton (Gossypium arboreum L.)
Source: BMC Genomics. 2017 Aug 31;18:675. doi: 10.1186/s12864-017-4066-y (PMC5580217; doi:10.1186/s12864-017-4066-y)
Supplement: Supplementary file 4 — Differentially expressed transcripts related to phytohormone signalling in Gossypium arboreum fuzzy-lintless line (Fl) as compared to fuzzy-linted (FL) at fibre at 0 dpa and 10 dpa. (DOC 156 kb) [file 12864_2017_4066_MOESM4_ESM.doc]

**Table S4: Differentially expressed transcripts related to phytohormone signalling in *Gossypium arboreum* fuzzy-lintless line (*Fl*) as compared to fuzzy-linted (*FL*) at fibre at 0 dpa and 10 dpa**.

| **Phytohormone family** | **Gene_ID** | **Fold change** | **Regulation** | **UniGene ID** | **GenBank ID** | **Closest Arabidopsis homolog** | **Description(TAIR database)** | ***E* value** |
| --- | --- | --- | --- | --- | --- | --- | --- | --- |
| **Fibre initiation stage (10 dpa)** | | | | | | | | |
| **Abscisic acid** | GhiAffx.7054.1.S1_at | 16.50139 | up | Ghi.9926 | DW509967.1 | AT5G20910.1 | RING/U-box superfamily protein | 4E-13 |
|  | Ghi.4983.1.A1_at | 6.4690266 | up | Ghi.4983 | DV849718 | AT3G19580.2 | zinc-finger protein 2 | 4.00E-31 |
|  | Ghi.1085.2.S1_at | 3.879253 | up | Ghi.4 | CD485897 | AT1G52340.1 | NAD(P)-binding Rossmann-fold superfamily protein | 3.00E-44 |
|  | GhiAffx.3411.1.A1_at | 3.6744685 | up | Ghi.11868 | DW497356.1 | AT1G27730.1 | salt tolerance zinc finger | 5E-15 |
|  | Ghi.8126.1.S1_x_at | 3.5254004 | up | Ghi.6690 | AY779339.1 | AT3G15210.1 | ethylene responsive element binding factor 4 | 1.00E-17 |
|  | GhiAffx.5954.1.S1_s_at | 3.5167634 | up | Ghi.9346 | DW225147.1 | AT5G27420.1 | carbon/nitrogen insensitive 1 | 5.00E-18 |
|  | Ghi.807.1.S1_s_at | 3.046491 | up | Ghi.17797 | DT465871 | AT1G27730.1 | salt tolerance zinc finger | 3.00E-31 |
| **Auxin** | GhiAffx.24550.1.S1_at | 5.7237525 | up | Ghi.16133 | DN818231 | AT5G54490.1 | pinoid-binding protein 1 | 5.00E-32 |
|  | Ghi.10493.1.S1_s_at | 4.426241 | up | Ghi.17551 | DT466412 | AT5G57560.1 | Xyloglucan endotransglucosylase/hydrolase family protein | 1.00E-63 |
|  | Ghi.5451.1.S1_at | 3.8172166 | up | Ghi.16253 | DQ122174.1 | AT4G11280.1 | 1-aminocyclopropane-1-carboxylic acid (acc) synthase 6 | 1.00E-177 |
|  | Ghi.10778.2.S1_at | 3.2575119 | up | Ghi.10778 | CA993737 | AT2G06850.1 | xyloglucan endotransglucosylase/hydrolase 4 | 8E-11 |
| **Brassinosteroid** | Ghi.664.1.S1_at | 5.093521 | up | Ghi.664 | CA992783 | AT3G61460.1 | brassinosteroid-responsive RING-H2 | 6.00E-56 |
| **Ethylene** | Ghi.10443.1.S1_at | 15.732822 | up | Ghi.10443 | DT049130 | AT4G39780.1 | Integrase-type DNA-binding superfamily protein | 4.00E-18 |
|  | Ghi.3673.1.S1_at | 10.481772 | up | Ghi.3673 | DT462887 | AT1G22190.1 | Integrase-type DNA-binding superfamily protein | 6.00E-18 |
|  | Ghi.9175.1.S1_at | 7.629576 | up | Ghi.9175 | DR462212 | AT5G61600.1 | ethylene response factor 104 | 1.00E-30 |
|  | Ghi.8749.1.S1_at | 4.591602 | up | Ghi.8749 | DT463517 | AT5G47230.1 | ethylene responsive element binding factor 5 | 1.00E-46 |
|  | GhiAffx.59715.1.S1_at | 4.221705 | up | Ghi.16596 | DW505344.1 | AT1G22190.1 | Integrase-type DNA-binding superfamily protein | 1E-10 |
|  | GhiAffx.61657.1.S1_at | 3.1391716 | up | Ghi.12559 | DW510592.1 | AT2G30860.1 | glutathione S-transferase PHI 9 | 1.00E-52 |
|  | Ghi.9880.2.A1_x_at | 3.1105497 | up | Ghi.9880 | DT047349 | AT5G47230.1 | ethylene responsive element binding factor 5 | 3.00E-17 |

<

| **Gibberellin** | GhiAffx.21219.1.A1_s_at | 4.078241 | up | Ghi.9948 | DW236303.1 | AT1G69530.2 | expansin A1 | 5.00E-45 |
| --- | --- | --- | --- | --- | --- | --- | --- | --- |
|  | Ghi.760.1.A1_x_at | 3.0644503 | up | Ghi.760 | DN827346 | AT1G80330.1 | gibberellin 3-oxidase 4 | 1.00E-17 |
| **Jasmonic acid** | Ghi.5304.2.A1_x_at | 3.652858 | down | Ghi.5304 | DT048257 | AT5G42650.1 | allene oxide synthase | 5E-11 |
| **Salicylic acid** | Ghi.9192.1.S1_s_at | 4.986691 | up | Ghi.9192 | DT468825 | AT1G80840.1 | WRKY DNA-binding protein 40 | 3.00E-51 |
|  | GhiAffx.5925.1.S1_at | 3.7370589 | up | Ghi.12812 | DW502097.1 | AT3G61190.2 | BON association protein 1 | 6.00E-23 |
|  | Ghi.2608.2.A1_at | 3.6166418 | up | Ghi.2608 | DT463212 | AT2G40000.1 | ortholog of sugar beet HS1 PRO-1 2 | 2.00E-28 |
|  | Ghi.1016.4.S1_s_at | 3.5463805 | up | Ghi.10821 | DT468576 | AT2G29420.1 | glutathione S-transferase tau 7 | 4.00E-23 |
| **Fibre elongation stage (10 dpa)** | | | | | | | | |
| **Abscisic acid** | Ghi.9152.1.S1_at | 10.333591 | down | Ghi.9152 | DT462541 | AT3G18130.1 | receptor for activated C kinase 1C | 4E-10 |
|  | Ghi.4.1.A1_at | 7.606558 | down | Ghi.4 | CK987701 | AT1G52340.1 | NAD(P)-binding Rossmann-fold superfamily protein | 4.00E-65 |
|  | Ghi.7907.1.S1_s_at | 6.206703 | down | Ghi.7907 | AI055500 | AT4G27410.2 | NAC (No Apical Meristem) domain transcriptional regulator superfamily protein | 2.00E-85 |
|  | Ghi.6901.1.A1_s_at | 5.749808 | down | Ghi.6901 | CA992707 | AT1G27730.1 | salt tolerance zinc finger | 6E-12 |
|  | Ghi.10676.1.S1_s_at | 4.287772 | down | Ghi.10676 | DT567365 | AT1G01360.1 | regulatory component of ABA receptor 1 | 8.00E-78 |
|  | GhiAffx.31355.1.S1_s_at | 3.9368427 | down | Ghi.13637 | DT462536 | AT4G34138.1 | UDP-glucosyl transferase 73B1 | 9.00E-86 |
|  | Ghi.807.1.S1_s_at | 3.851732 | down | Ghi.17797 | DT465871 | AT1G27730.1 | salt tolerance zinc finger | 3.00E-31 |
|  | GhiAffx.7814.1.S1_s_at | 3.7005303 | down | Ghi.21981 | DW516033.1 | AT5G25610.1 | BURP domain-containing protein | 1.00E-41 |
|  | GhiAffx.15490.1.S1_at | 3.6327517 | down | Ghi.18655 | DW237402.1 | AT2G22570.1 | nicotinamidase 1 | 4.00E-89 |
|  | Ghi.9176.3.A1_at | 3.419338 | down | Ghi.9176 | DT464586 | AT1G52340.1 | NAD(P)-binding Rossmann-fold superfamily protein | 5.00E-32 |
|  | GhiAffx.60835.1.S1_at | 3.2344124 | down | Ghi.12975 | DW506319.1 | AT5G45820.1 | CBL-interacting protein kinase 20 | 6.00E-60 |
|  | Ghi.8524.1.S1_at | 3.0212526 | down | Ghi.18592 | DT048651 | AT5G10560.1 | Glycosyl hydrolase family protein | 5.00E-110 |
| **Auxin** | Ghi.6236.2.S1_s_at | 7.3609204 | up | Ghi.6236 | D88413.1 | AT2G06850.1 | xyloglucan endotransglucosylase/hydrolase 4 | 2.00E-83 |
|  | GraAffx.8958.1.S1_s_at | 6.7851157 | up |  | CO087973 | AT2G06850.1 | xyloglucan endotransglucosylase/hydrolase 4 | 2.00E-65 |
|  | Ghi.6236.1.S1_s_at | 5.846538 | up | Ghi.6236 | AY189971.1 | AT2G06850.1 | xyloglucan endotransglucosylase/hydrolase 4 | 2.00E-75 |
|  | GhiAffx.6062.1.S1_at | 3.1669397 | up | Ghi.15960 | DW495992.1 | AT5G20820.1 | SAUR-like auxin-responsive protein family | 1.00E-22 |
|  | GraAffx.27319.1.S1_s_at | 3.1551235 | up |  | CO089724 | AT5G57560.1 | Xyloglucan endotransglucosylase/hydrolase family protein | 3.00E-50 |

|  | GraAffx.1410.1.S1_at | 11.052195 | down |  | CO125821 | AT5G65980.1 | Auxin efflux carrier family protein | 5.00E-103 |
| --- | --- | --- | --- | --- | --- | --- | --- | --- |
|  | Ghi.3578.1.S1_s_at | 10.348414 | down | Ghi.3578 | DT567472 | AT4G36740.1 | homeobox protein 40 | 1.00E-45 |
|  | Ghi.6543.1.S1_s_at | 8.538899 | down | Ghi.6543 | DN780646 | AT4G14550.1 | indole-3-acetic acid inducible 14 | 2.00E-78 |
|  | Ghi.3446.1.A1_at | 5.4198785 | down | Ghi.3446 | DT462755 | AT1G56010.2 | NAC domain containing protein 1 | 3E-11 |
|  | GhiAffx.19570.1.S1_at | 5.245414 | down | Ghi.14271 | DW489734.1 | AT4G37070.3 | Acyl transferase/acyl hydrolase/lysophospholipase superfamily protein | 2.00E-56 |
|  | GhiAffx.2060.1.S1_at | 4.663229 | down |  | DW477541.1 | AT1G59870.1 | ABC-2 and Plant PDR ABC-type transporter family protein | 4.00E-124 |
|  | Ghi.967.1.S1_s_at | 4.3808484 | down | Ghi.967 | AI728289 | AT2G04160.1 | Subtilisin-like serine endopeptidase family protein | 1.00E-66 |
|  | Ghi.4725.1.S1_s_at | 3.6865726 | down | Ghi.4725 | AI726805 | AT2G06850.1 | xyloglucan endotransglucosylase/hydrolase 4 | 7.00E-97 |
|  | Ghi.6547.1.S1_s_at | 3.5325873 | down |  | DR458804 | AT4G31500.1 | cytochrome P450, family 83, subfamily B, polypeptide 1 | 2.00E-52 |
|  | Gra.377.1.A1_s_at | 3.4132838 | down | Gra.2265 | CO085937 | AT2G06850.1 | xyloglucan endotransglucosylase/hydrolase 4 | 8.00E-60 |
|  | GhiAffx.6395.1.S1_s_at | 3.1220052 | down | Ghi.13987 | DW484802.1 | AT4G14550.1 | indole-3-acetic acid inducible 14 | 1.00E-83 |
|  | Ghi.9281.1.A1_s_at | 3.1192684 | down | Ghi.17961 | DT047152 | AT4G36740.1 | homeobox protein 40 | 5.00E-51 |
|  | GhiAffx.28716.1.S1_at | 3.0640888 | down | Ghi.15689 | DW512426.1 | AT4G00880.1 | SAUR-like auxin-responsive protein family | 5.00E-30 |
| **Brassinosteroid** | GhiAffx.49772.1.A1_at | 9.510919 | up |  | DT464025 | AT4G39400.1 | Leucine-rich receptor-like protein kinase family protein | 6E-12 |
|  | Ghi.6538.1.S1_at | 14.719816 | down | Ghi.6538 | CD485949 | AT1G61110.1 | NAC domain containing protein 25 | 7.00E-69 |
|  | GhiAffx.31391.1.S1_s_at | 8.354315 | down |  | DW481920.1 | AT1G07340.1 | sugar transporter 2 | 2.00E-29 |
|  | Ghi.6822.1.A1_s_at | 7.3133745 | down | Ghi.6822 | CA993006 | AT2G36800.1 | don-glucosyltransferase 1 | 2.00E-53 |
|  | GhiAffx.22064.1.S1_at | 6.3178463 | down | Ghi.12850 | DW498676.1 | AT1G17060.1 | cytochrome p450 72c1 | 1.00E-63 |
|  | Ghi.9328.1.S1_s_at | 5.254816 | down | Ghi.4821 | DT048550 | AT1G61110.1 | NAC domain containing protein 25 | 2.00E-49 |
|  | GbaAffx.197.1.S1_s_at | 4.1984053 | down |  | AY279356.1 | AT3G13380.1 | BRI1-like 3 | 2.00E-21 |
|  | Ghi.44.1.A1_at | 3.345396 | down | Ghi.44 | DR463380 | AT1G71830.1 | somatic embryogenesis receptor-like kinase 1 | 8.00E-31 |
|  | GhiAffx.30941.1.S1_s_at | 3.2078433 | down | Ghi.13511 | DW482613.1 | AT1G13260.1 | related to ABI3/VP1 1 | 2.00E-69 |
|  | Gra.2844.1.S1_s_at | 3.043563 | down | Gra.17 | CO087535 | AT5G13170.1 | senescence-associated gene 29 | 2.00E-68 |

| **Cytokinin** | GhiAffx.44018.1.S1_at | 12.242164 | down | Ghi.15557 | DW502867.1 | AT1G30260.1 | Galactosyltransferase family protein | 7E-11 |
| --- | --- | --- | --- | --- | --- | --- | --- | --- |
|  | Ghi.7874.1.S1_s_at | 10.277513 | down | Ghi.16277 /// Ghi.7874 | AY962572.1 | AT3G16770.1 | ethylene-responsive element binding protein | 7.00E-36 |
|  | GbaAffx.196.1.A1_s_at | 7.6331277 | down |  | AY572462.1 | AT3G16770.1 | ethylene-responsive element binding protein | 3.00E-30 |
| **Ethylene** | Ghi.7950.1.S1_at | 81.1664 | down | Ghi.16267 | AY366083.1 | AT5G19880.1 | Peroxidase superfamily protein | 2.00E-85 |
|  | Gra.2141.1.S1_s_at | 39.88526 | down |  | CO123471 | AT1G05010.1 | ethylene-forming enzyme | 2.00E-119 |
|  | Ghi.8023.1.S1_at | 39.720764 | down | Ghi.16693 | DQ116443.1 | AT1G62380.1 | ACC oxidase 2 | 5.00E-123 |
|  | Ghi.6953.1.S1_s_at | 15.015603 | down | Ghi.16374 | DQ116442.1 | AT1G05010.1 | ethylene-forming enzyme | 4.00E-122 |
|  | Ghi.8448.1.S1_x_at | 11.607274 | down | Ghi.8448 | AF521240.1 | AT5G19780.1 | tubulin alpha-5 | 2.00E-92 |
|  | Ghi.1043.4.S1_at | 8.142763 | down | Ghi.17859 | DT463348 | AT5G19880.1 | Peroxidase superfamily protein | 4.00E-45 |
|  | Ghi.8105.1.A1_s_at | 7.123931 | down | Ghi.8105 | AF488305.1 | AT5G19880.1 | Peroxidase superfamily protein | 4.00E-89 |
|  | Ghi.5022.4.A1_s_at | 5.890732 | down | Ghi.17602 | DT049392 | AT1G73500.1 | MAP kinase kinase 9 | 1.00E-39 |
|  | Ghi.10753.1.S1_at | 5.6141815 | down | Ghi.10753 | DN760125 | AT4G27450.1 | Aluminium induced protein with YGL and LRDR motifs | 5.00E-85 |
|  | GhiAffx.25472.1.A1_s_at | 5.6117935 | down |  | DW516477.1 | AT3G04720.1 | pathogenesis-related 4 | 2.00E-76 |
|  | Ghi.5775.1.S1_s_at | 5.514077 | down | Ghi.5775 | DT455881 | AT3G23150.1 | Signal transduction histidine kinase, hybrid-type, ethylene sensor | 3.00E-45 |
|  | Ghi.8110.1.S1_at | 4.90623 | down | Ghi.8110 | AY311597.1 | AT5G19880.1 | Peroxidase superfamily protein | 7.00E-71 |
|  | Ghi.10747.1.S1_at | 4.822069 | down | Ghi.10747 | DV850132 | AT5G47220.1 | ethylene responsive element binding factor 2 | 7.00E-45 |
|  | GhiAffx.7865.1.S1_at | 4.5940685 | down |  | DW503266.1 | AT5G07580.1 | Integrase-type DNA-binding superfamily protein | 1.00E-34 |
|  | Ghi.798.1.S1_s_at | 4.1597486 | down | Ghi.16386 | DQ116444.1 | AT1G05010.1 | ethylene-forming enzyme | 1.00E-131 |
|  | GhiAffx.28739.1.S1_s_at | 4.0409465 | down | Ghi.15180 | DW502086.1 | AT5G47220.1 | ethylene responsive element binding factor 2 | 1.00E-30 |
|  | Ghi.6548.1.S1_s_at | 3.5500994 | down | Ghi.16406 | AY207316.1 | AT5G58350.1 | with no lysine (K) kinase 4 | 2.00E-80 |
| **Gibberellin** | Gra.1544.1.A1_s_at | 5.5872493 | down |  | CO091149 | AT3G63010.1 | alpha/beta-Hydrolases superfamily protein | 4.00E-145 |
|  | Gra.2150.1.S1_s_at | 4.206167 | down | Gra.2150 | CO085918 | AT5G27320.1 | alpha/beta-Hydrolases superfamily protein | 7.00E-33 |
|  | GhiAffx.10920.2.S1_at | 3.8752277 | down | Ghi.16091 | DW237583.1 | AT2G36830.1 | 1, GAMMA-TIP1 | 9.00E-18 |
|  | GhiAffx.1402.1.S1_at | 3.4361563 | down | Ghi.14499 | DW231451.1 | AT1G15550.1 | gibberellin 3-oxidase 1 | 9.00E-75 |
|  | GhiAffx.36084.1.A1_at | 3.337532 | down | Ghi.21982 | DW516195.1 | AT2G04240.2 | RING/U-box superfamily protein | 2.00E-46 |

| **Jasmonic acid** | Ghi.9193.2.A1_at | 12.23719 | down | Ghi.9193 | DT469110 | AT3G56400.1 | WRKY DNA-binding protein 70 | 2.00E-27 |
| --- | --- | --- | --- | --- | --- | --- | --- | --- |
|  | Ghi.1847.1.S1_at | 7.26273 | down | Ghi.1847 | DV850045 | AT2G26690.1 | Major facilitator superfamily protein | 1.00E-28 |
|  | Ghi.3135.1.S1_at | 7.021998 | down | Ghi.3135 | DT469074 | AT2G26690.1 | Major facilitator superfamily protein | 3.00E-55 |
|  | GhiAffx.48583.1.S1_at | 6.144695 | down |  | AI055122 | AT3G06490.1 | myb domain protein 108 | 2.00E-69 |
|  | Ghi.3264.1.S1_s_at | 5.772931 | down | Ghi.3264 | DT466083 | AT5G08790.1 | NAC (No Apical Meristem) domain transcriptional regulator superfamily protein | 3.00E-86 |
|  | GarAffx.37202.1.S1_x_at | 3.5024858 | down |  | U23205.1 | AT2G24210.1 | terpene synthase 10 | 3.00E-66 |
| **Salicylic acid** | Ghi.10795.1.S1_s_at | 3.265911 | up | Ghi.6472 | CD486563 | AT4G12470.1 | azelaic acid induced 1 | 1.00E-16 |
|  | Ghi.6472.1.A1_s_at | 3.0664685 | up | Ghi.6472 | CD485893 | AT4G12470.1 | azelaic acid induced 1 | 3E-12 |
|  | Ghi.6088.2.A1_s_at | 10.754808 | down | Ghi.6088 | DV849489 | AT3G45640.1 | mitogen-activated protein kinase 3 | 2.00E-28 |
|  | Ghi.1016.4.S1_s_at | 7.797561 | down | Ghi.10821 | DT468576 | AT2G29420.1 | glutathione S-transferase tau 7 | 4.00E-23 |
|  | GhiAffx.1859.1.S1_at | 7.2392125 | down |  | DT468306 | AT5G46350.1 | WRKY DNA-binding protein 8 | 5.00E-25 |
|  | GhiAffx.5935.2.S1_s_at | 7.227638 | down | Ghi.9213 | DW235907.1 | AT2G40000.1 | ortholog of sugar beet HS1 PRO-1 2 | 4.00E-51 |
|  | Ghi.6088.1.S1_s_at | 6.3369107 | down | Ghi.6088 | DT466983 | AT3G45640.1 | mitogen-activated protein kinase 3 | 1.00E-137 |
|  | Ghi.6539.1.S1_s_at | 6.073285 | down | Ghi.9213 | CD485942 | AT2G40000.1 | ortholog of sugar beet HS1 PRO-1 2 | 5.00E-34 |
|  | Ghi.2608.2.A1_at | 5.8584695 | down | Ghi.2608 | DT463212 | AT2G40000.1 | ortholog of sugar beet HS1 PRO-1 2 | 2.00E-28 |
|  | GhiAffx.30199.1.S1_at | 3.6616116 | down | Ghi.15715 | DW506814.1 | AT5G46350.1 | WRKY DNA-binding protein 8 | 7.00E-32 |
|  | Ghi.779.1.S1_at | 3.0192256 | down | Ghi.779 | DN800052 | AT2G29420.1 | glutathione S-transferase tau 7 | 3.00E-31 |
