# Supplementary material for: Transcript profiling of genes expressed during fibre development in diploid cotton (Gossypium arboreum L.)
Source: BMC Genomics. 2017 Aug 31;18:675. doi: 10.1186/s12864-017-4066-y (PMC5580217; doi:10.1186/s12864-017-4066-y)
Supplement: Supplementary file 5 — Overview of differentially expressed transcripts present in various metabolic processes based on MapMan (version 3.5) visualization software Gossypium arboreum fuzzy-lintless line (Fl) at 0 dpa (DOC 103 kb) [file 12864_2017_4066_MOESM5_ESM.doc]

**Table S5: Overview of differentially expressed transcripts present in various metabolic processes based on MapMan (version 3.5) visualization software *Gossypium arboreum* fuzzy-lintless line (*Fl*) at 0 dpa.**

| **Bincode** | **Name** | **Bin details** | **Bin description** | **Gene id** | **Regulation fold change** | **Unigene id** | **Genbank id** | **Closest arabidopsis homolog** | **Description** |
| --- | --- | --- | --- | --- | --- | --- | --- | --- | --- |
| **1** | PS | 1.1.1.1 | PS.lightreaction.photosystem II.LHC-II | Ghi.10635.1.S1_x_at | 3.0207536 | Ghi.6780 | DN760160 | AT2G34430.1 | light-harvesting chlorophyll-protein complex II subunit B1 |
|  |  | 1.1.3.3 | PS.lightreaction.cytochrome b6/f.cytochrome b6 (CYB6) | GhiAffx.1119.2.S1_at | 3.2936802 | Ghi.15519 | DW225844.1 | ATCG00720.1 | photosynthetic electron transfer B |
|  |  | 1.3.2 | PS.calvin cycle.rubisco small subunit | Ghi.1513.1.S1_x_at | 3.6074338 | Ghi.1513 | CA994331 | AT5G38430.1 | Ribulose bisphosphate carboxylase (small chain) family protein |
| **10** | Cell wall | 10.7 | cell wall.modification | Ghi.10493.1.S1_s_at | 4.426241 | Ghi.17551 | DT466412 | AT5G57560.1 | Xyloglucan endotransglucosylase/hydrolase family protein |
|  |  | 10.7 | cell wall.modification | GhiAffx.21219.1.A1_s_at | 4.078241 | Ghi.9948 | DW236303.1 | AT1G69530.2 | expansin A1 |
| **12** | N-metabolism | 12.1.1 | N-metabolism.nitrate metabolism.NR | Ghi.5852.3.S1_s_at | -5.147951 | Ghi.5852 | CO498687 | AT3G01910.3 | sulfite oxidase |
| **14** | S-assimilation | 14.5 | S-assimilation.sulfite oxidase | Ghi.5852.3.S1_s_at | -5.147951 | Ghi.5852 | CO498687 | AT3G01910.3 | sulfite oxidase |
| **17** | Hormone metabolism | 17.5.1.1 | hormone metabolism.ethylene.synthesis-degradation.1-aminocyclopropane-1-carboxylate synthase | Ghi.5451.1.S1_at | 3.8172166 | Ghi.16253 | DQ122174.1 | AT4G11280.1 | 1-aminocyclopropane-1-carboxylic acid (acc) synthase 6 |
|  |  | 17.5.2 | hormone metabolism.ethylene.signal transduction | Ghi.9880.2.A1_x_at | 3.1105497 | Ghi.9880 | DT047349 | AT5G47230.1 | ethylene responsive element binding factor 5 |
|  |  | 17.5.2 | hormone metabolism.ethylene.signal transduction | Ghi.9175.1.S1_at | 7.629576 | Ghi.9175 | DR462212 | AT5G51190.1 | Integrase-type DNA-binding superfamily protein |
|  |  | 17.5.2 | hormone metabolism.ethylene.signal transduction | Ghi.8126.1.S1_x_at | 3.5254004 | Ghi.6690 | AY779339.1 | AT3G15210.1 | ethylene responsive element binding factor 4 |
|  |  | 17.5.2 | hormone metabolism.ethylene.signal transduction | Ghi.8749.1.S1_at | 4.591602 | Ghi.8749 | DT463517 | AT5G47230.1 | ethylene responsive element binding factor 5 |
|  |  | 17.6.1 | hormone metabolism.gibberelin.synthesis-degradation | Ghi.760.1.A1_x_at | 3.0644503 | Ghi.760 | DN827346 | AT1G52820.1 | 2-oxoglutarate (2OG) and Fe(II)-dependent oxygenase superfamily protein |

| **20** | Stress | 20.2.1 | stress.abiotic.heat | Ghi.10778.2.S1_at | 3.2575119 | Ghi.10778 | CA993737 | AT2G17880.1 | Chaperone DnaJ-domain superfamily protein |
| --- | --- | --- | --- | --- | --- | --- | --- | --- | --- |
| **22** | Polyamine metabolism | 22.1.6 | polyamine metabolism.synthesis.spermidine synthase | GhiAffx.2527.1.S1_s_at | -151.1868 | Ghi.13939 | DW497370.1 | AT5G53120.6 | spermidine synthase 3 |
| **26** | Misc | 26.9 | misc.glutathione S transferases | Ghi.1016.4.S1_s_at | 3.5463805 | Ghi.10821 | DT468576 | AT3G09270.1 | glutathione S-transferase TAU 8 |
|  |  | 26.9 | misc.glutathione S transferases | GhiAffx.61657.1.S1_at | 3.1391716 | Ghi.12559 | DW510592.1 | AT5G17220.1 | glutathione S-transferase phi 12 |
|  |  | 26.22 | misc.short chain dehydrogenase/reductase (SDR) | Ghi.1085.2.S1_at | 3.879253 | Ghi.4 | CD485897 | AT3G26770.1 | NAD(P)-binding Rossmann-fold superfamily protein |
| **27** | RNA | 27.1.19 | RNA.processing.ribonucleases | Ghi.8610.1.S1_s_at | 5.069452 | Ghi.8610 | CA992956 | AT5G22250.1 | Polynucleotidyl transferase, ribonuclease H-like superfamily protein |
|  |  | 27.1.19 | RNA.processing.ribonucleases | GbaAffx.201.1.S1_s_at | 3.6479692 |  | AY560553.1 | |  |
|  |  | 27.3.3 | RNA.regulation of transcription.AP2/EREBP, APETALA2/Ethylene-responsive element binding protein family | Ghi.9880.2.A1_x_at | 3.1105497 | Ghi.9880 | DT047349 | AT5G47230.1 | ethylene responsive element binding factor 5 |
|  |  | 27.3.3 | RNA.regulation of transcription.AP2/EREBP, APETALA2/Ethylene-responsive element binding protein family | Ghi.10443.1.S1_at | 15.732822 | Ghi.10443 | DT049130 | AT1G19210.1 | Integrase-type DNA-binding superfamily protein |
|  |  | 27.3.3 | RNA.regulation of transcription.AP2/EREBP, APETALA2/Ethylene-responsive element binding protein family | Ghi.3673.1.S1_at | 10.481772 | Ghi.3673 | DT462887 | AT1G19210.1 | Integrase-type DNA-binding superfamily protein |
|  |  | 27.3.3 | RNA.regulation of transcription.AP2/EREBP, APETALA2/Ethylene-responsive element binding protein family | GhiAffx.59715.1.S1_at | 4.221705 | Ghi.16596 | DW505344.1 | AT1G19210.1 | Integrase-type DNA-binding superfamily protein |
|  |  | 27.3.3 | RNA.regulation of transcription.AP2/EREBP, APETALA2/Ethylene-responsive element binding protein family | Ghi.8126.1.S1_x_at | 3.5254004 | Ghi.6690 | AY779339.1 | AT3G15210.1 | ethylene responsive element binding factor 4 |
|  |  | 27.3.11 | RNA.regulation of transcription.C2H2 zinc finger family | Ghi.4983.1.A1_at | 6.4690266 | Ghi.4983 | DV849718 | AT5G04340.1 | zinc finger of Arabidopsis thaliana 6 |

|  |  | 27.3.11 | RNA.regulation of transcription.C2H2 zinc finger family | GhiAffx.3411.1.A1_at | 3.6744685 | Ghi.11868 | DW497356.1 | AT2G28710.1 | C2H2-type zinc finger family protein |
| --- | --- | --- | --- | --- | --- | --- | --- | --- | --- |
|  |  | 27.3.11 | RNA.regulation of transcription.C2H2 zinc finger family | Ghi.807.1.S1_s_at | 3.046491 | Ghi.17797 | DT465871 | AT1G27730.1 | salt tolerance zinc finger |
|  |  | 27.3.32 | RNA.regulation of transcription.WRKY domain transcription factor family | Ghi.9192.1.S1_s_at | 4.986691 | Ghi.9192 | DT468825 | AT1G80840.1 | WRKY DNA-binding protein 40 |
| **29** | Protein | 29.5.4 | protein.degradation.aspartate protease | Ghi.3784.2.S1_s_at | 3.000441 | Ghi.3784 | DT457597 | AT5G19120.1 | Eukaryotic aspartyl protease family protein |
|  |  | 29.5.11.4.2 | protein.degradation.ubiquitin.E3.RING | GhiAffx.5954.1.S1_s_at | 3.5167634 | Ghi.9346 | DW225147.1 | AT3G16720.1 | TOXICOS EN LEVADURA 2 |
|  |  | 29.5.11.4.2 | protein.degradation.ubiquitin.E3.RING | Ghi.664.1.S1_at | 5.093521 | Ghi.664 | CA992783 | AT3G61460.1 | brassinosteroid-responsive RING-H2 |
|  |  | 29.5.11.4.2 | protein.degradation.ubiquitin.E3.RING | GhiAffx.7054.1.S1_at | 16.50139 | Ghi.9926 | DW509967.1 | AT5G59550.1 | zinc finger (C3HC4-type RING finger) family protein |
| **30** | Signalling | 30.1 | signalling.in sugar and nutrient physiology | GhiAffx.63620.1.S1_at | 4.9628625 | Ghi.15977 | DW505500.1 | AT5G55850.3 | RPM1-interacting protein 4 (RIN4) family protein |
|  |  | 30.3 | signalling.calcium | GhiAffx.24550.1.S1_at | 5.7237525 | Ghi.16133 | DN818231 | AT4G27280.1 | Calcium-binding EF-hand family protein |
|  |  | 30.3 | signalling.calcium | Ghi.3763.1.A1_s_at | 7.1966577 | Ghi.3763 | DT461952 | AT3G63380.1 | ATPase E1-E2 type family protein / haloacid dehalogenase-like hydrolase family protein |
| **33** | Development | 33.99 | development.unspecified | GhiAffx.5925.1.S1_at | 3.7370589 | Ghi.12812 | DW502097.1 | AT2G45760.1 | BON association protein 2 |
| **34** | Transport | 34.13 | transport.peptides and oligopeptides | GhiAffx.10850.1.A1_s_at | -4.545873 | Ghi.15954 | DN799961 | AT5G62680.1 | Major facilitator superfamily protein |
|  |  | 34.99 | transport.misc | Ghi.7853.1.S1_at | -5.828306 | Ghi.16284 | AF443118.1 | AT1G01630.1 | Sec14p-like phosphatidylinositol transfer family protein |
| **35** | Not assigned | 35.2 | not assigned.unknown | Ghi.4920.1.A1_at | 14.513754 | Ghi.4920 | DN758194 | |  |
|  |  | 35.2 | not assigned.unknown | Ghi.5587.1.S1_at | -3.622697 | Ghi.5587 | CA993040 |  |  |
|  |  | 35.2 | not assigned.unknown | GhiAffx.19697.1.A1_s_at | 7.9164195 | Ghi.10366 | DW227913.1 | AT3G11760.1 | unknown protein |
|  |  | 35.2 | not assigned.unknown | Ghi.1319.1.S1_s_at | -3.094999 | Ghi.16399 | AF118230.1 | AT3G15353.1 | metallothionein 3 |
|  |  | 35.2 | not assigned.unknown | GhiAffx.4679.1.A1_a_at | -3.086619 | Ghi.12168 | DW510621.1 | AT4G01935.1 | unknown protein |
|  |  | 35.2 | not assigned.unknown | Ghi.3284.1.S1_s_at | 7.9350553 | Ghi.3284 | DT466688 | AT5G12010.1 | unknown protein |
|  |  | 35.2 | not assigned.unknown | Ghi.2608.2.A1_at | 3.6166418 | Ghi.2608 | DT463212 | AT3G55840.1 | Hs1pro-1 protein |
|  |  | 35.2 | not assigned.unknown | Ghi.106.1.S1_s_at | 7.706848 | Ghi.106 | DR462832 |  |  |

|  |  | 35.2 | not assigned.unknown | Ghi.10366.2.S1_at | 4.9414372 | Ghi.10366 | AW561926 | AT2G25460.1 | CONTAINS InterPro DOMAIN/s: C2 calcium-dependent membrane targeting (InterPro:IPR000008) |
| --- | --- | --- | --- | --- | --- | --- | --- | --- | --- |
|  |  | 35.2 | not assigned.unknown | GhiAffx.7649.1.S1_s_at | 10.767832 | Ghi.18614 | DW478800.1 | AT5G12010.1 | unknown protein |
|  |  | 35.2 | not assigned.unknown | Ghi.5304.2.A1_x_at | -3.652858 | Ghi.5304 | DT048257 | AT5G42650.1 | allene oxide synthase |
|  |  | 35.2 | not assigned.unknown | Ghi.1092.3.S1_s_at | 12.998623 | Ghi.18610 | DT467839 | AT4G29780.1 | unknown protein |
|  |  | 35.2 | not assigned.unknown | GhiAffx.1119.1.S1_at | 3.3052182 | Ghi.13018 | DW242880.1 | |  |
|  |  | 35.2 | not assigned.unknown | Ghi.6748.1.A1_at | 3.456143 | Ghi.6748 | CA993446 | AT3G57450.1 | unknown protein |
|  |  | 35.2 | not assigned.unknown | GhiAffx.22562.1.A1_at | -28.21745 | Ghi.13380 | DW238476.1 | AT2G04420.1 | Polynucleotidyl transferase, ribonuclease H-like superfamily protein |
|  |  | 35.2 | not assigned.unknown | Ghi.1092.1.A1_x_at | 15.018939 | Ghi.18632 | DT462950 | AT4G29780.1 | unknown protein |
|  |  | 35.2 | not assigned.unknown | GhiAffx.25239.1.S1_at | -3.786133 | Ghi.15924 | DW515297.1 | |  |
|  |  | 35.2 | not assigned.unknown | Ghi.884.1.S1_at | -3.023619 | Ghi.884 | DR453639 | AT5G66985.1 | unknown protein |
|  |  | 35.2 | not assigned.unknown | Ghi.8931.1.S1_a_at | -87.72372 | Ghi.8931 | DT457712 |  |  |
|  |  | 35.2 | not assigned.unknown | GhiAffx.11775.1.S1_at | 10.959008 | Ghi.16170 | DW224036.1 | AT5G17350.1 | unknown protein |
|  |  | 35.2 | not assigned.unknown | Ghi.9149.4.A1_s_at | 3.5930614 | Ghi.17231 | CA993608 | AT1G32920.1 | unknown protein |
|  |  | 35.2 | not assigned.unknown | GhiAffx.52646.1.S1_at | 8.782509 | Ghi.11801 | DW500254.1 | AT4G13395.1 | ROTUNDIFOLIA like 12 |
