# Supplementary material for: Transcript profiling of genes expressed during fibre development in diploid cotton (Gossypium arboreum L.)
Source: BMC Genomics. 2017 Aug 31;18:675. doi: 10.1186/s12864-017-4066-y (PMC5580217; doi:10.1186/s12864-017-4066-y)
Supplement: Supplementary file 6 — Overview of differentially expressed transcripts present in various metabolic processes based on MapMan (version 3.5) visualization software in Gossypium arboreum fuzzy-lintless line (Fl) at 10 dpa. (DOC 313 kb) [file 12864_2017_4066_MOESM6_ESM.doc]

**Table S6: Overview of differentially expressed transcripts present in various metabolic processes based on MapMan (version 3.5) visualization software in *Gossypium arboreum* fuzzy-lintless line (*Fl*) at 10 dpa.**

| **Bincode** | **Name** | **Bin details** | **Bin description** | **Gene id** | **Unigene id** | **Regulation** | **Genbank id** | **Closest arabidopsis homolog** | **Description** |
| --- | --- | --- | --- | --- | --- | --- | --- | --- | --- |
| **3** | Minor CHO metabolism | 3.2.2 | minor CHO metabolism.trehalose.TPP | Ghi.912.3.A1_at | Ghi.912 | -4.7067513 | DT468062 | AT5G65140.1 | Haloacid dehalogenase-like hydrolase (HAD) superfamily protein |
|  |  | 3.2.3 | minor CHO metabolism.trehalose.potential TPS/TPP | Ghi.3451.2.A1_at | Ghi.3451 | -3.8055377 | DT465672 | AT1G23870.1 | trehalose-phosphatase/synthase 9 |
| **6** | Gluconeogenese/ glyoxylate cycle | 6.2 | gluconeogenese/ glyoxylate cycle.malate synthase | Ghi.8123.1.S1_at | Ghi.8123 | -8.612653 | X52305.1 | AT5G03860.2 | malate synthase |
| **8** | TCA / org. transformation | 8.3 | TCA / org. transformation.carbonic anhydrases | GhiAffx.1082.1.S1_at | Ghi.12989 | -3.4974022 | DW511203.1 | AT4G33580.1 | beta carbonic anhydrase 5 |
| **9** | Mitochondrial electron transport / ATP synthesis | 9.9 | mitochondrial electron transport / ATP synthesis.F1-ATPase | GhiAffx.52777.1.S1_at | Ghi.15921 | 4.350262 | DW515277.1 | ATCG00480.1 | ATP synthase subunit beta |
| **10** | Cell wall | 10.1.2 | cell wall.precursor synthesis.UGE | GhiAffx.19125.1.A1_at | | -19.172623 | DW487672.1 | AT1G12780.1 | UDP-D-glucose/UDP-D-galactose 4-epimerase 1 |
|  |  | 10.1.2 | cell wall.precursor synthesis.UGE | Gra.2050.1.S1_s_at | Gra.2050 | -3.2317345 | CO126131 | AT1G12780.1 | UDP-D-glucose/UDP-D-galactose 4-epimerase 1 |
|  |  | 10.6 | cell wall.degradation | GhiAffx.60253.1.S1_at | Ghi.14666 | -3.0274034 | DW503386.1 | AT5G62150.1 | peptidoglycan-binding LysM domain-containing protein |
|  |  | 10.6.2 | cell wall.degradation.mannan-xylose-arabinose-fucose | Ghi.8524.1.S1_at | Ghi.18592 | -3.0212526 | DT048651 | AT5G49360.1 | beta-xylosidase 1 |
|  |  | 10.6.3 | cell wall.degradation.pectate lyases and polygalacturonases | GhiAffx.7814.1.S1_s_at | Ghi.21981 | -3.7005303 | DW516033.1 | AT1G49320.1 | unknown seed protein like 1 |
|  |  | 10.7 | cell wall.modification | Ghi.4532.1.A1_at | Ghi.4532 | -5.7609086 | DR457588 | AT4G18990.1 | xyloglucan endotransglucosylase/hydrolase 29 |
|  |  | 10.7 | cell wall.modification | Ghi.6465.2.S1_at | Ghi.6465 | -55.049065 | CD485906 | AT4G17030.1 | expansin-like B1 |
|  |  | 10.7 | cell wall.modification | Ghi.6236.2.S1_s_at | Ghi.6236 | 7.3609204 | D88413.1 | AT4G37800.1 | xyloglucan endotransglucosylase/hydrolase 7 |
|  |  | 10.7 | cell wall.modification | GraAffx.27319.1.S1_s_at | | 3.1551235 | CO089724 | AT2G36870.1 | xyloglucan endotransglucosylase/hydrolase 32 |

|  |  | 10.7 | cell wall.modification | Ghi.6236.1.S1_s_at | Ghi.6236 | 5.846538 | AY189971.1 | AT4G37800.1 | xyloglucan endotransglucosylase/hydrolase 7 |
| --- | --- | --- | --- | --- | --- | --- | --- | --- | --- |
|  |  | 10.7 | cell wall.modification | Ghi.4725.1.S1_s_at | Ghi.4725 | -3.6865726 | AI726805 | AT5G13870.1 | xyloglucan endotransglucosylase/hydrolase 5 |
|  |  | 10.7 | cell wall.modification | Gra.377.1.A1_s_at | Gra.2265 | -3.4132838 | CO085937 | AT2G06850.1 | xyloglucan endotransglucosylase/hydrolase 4 |
|  |  | 10.7 | cell wall.modification | GraAffx.8958.1.S1_s_at | | 6.7851157 | CO087973 | AT4G37800.1 | xyloglucan endotransglucosylase/hydrolase 7 |
|  |  | 10.8.2 | cell wall.pectin*esterases.acetyl esterase | Ghi.5860.1.S1_s_at | Ghi.16418 | 46.186455 | CO494172 | AT2G04780.2 | FASCICLIN-like arabinoogalactan 7 |
| **11** | Lipid metabolism | 11.1.8 | lipid metabolism.FA synthesis and FA elongation.acyl coa ligase | Ghi.3184.1.S1_s_at | Ghi.1202 | -4.2506437 | DT468147 | AT3G16910.1 | acyl-activating enzyme 7 |
|  |  | 11.9.2 | lipid metabolism.lipid degradation.lipases | GhiAffx.24021.1.S1_at | | -3.3665998 | DW509098.1 | AT2G44970.2 | alpha/beta-Hydrolases superfamily protein |
|  |  | 11.9.2 | lipid metabolism.lipid degradation.lipases | Ghi.7293.1.S1_s_at | Ghi.7293 | -3.39789 | AI731856 | AT2G39420.1 | alpha/beta-Hydrolases superfamily protein |
|  |  | 11.9.4.2 | lipid metabolism.lipid degradation.beta-oxidation.acyl CoA DH | Ghi.7466.1.S1_at | Ghi.7466 | -3.141613 | AI730393 | AT3G51840.1 | acyl-CoA oxidase 4 |
| **12** | N-metabolism | 12.3.1 | N-metabolism.N-degradation.glutamate dehydrogenase | Ghi.6500.1.S1_at | Ghi.6500 | -3.9809632 | AI728753 | AT3G03910.1 | glutamate dehydrogenase 3 |
| **13** | Amino acid metabolism | 13.1.1.1.1 | amino acid metabolism.synthesis.central amino acid metabolism.GABA.Glutamate decarboxylase | Ghi.7536.1.S1_s_at | Ghi.7536 | -3.3061235 | AI729592 | AT2G02010.1 | glutamate decarboxylase 4 |
|  |  | 13.1.3.1 | amino acid metabolism.synthesis.aspartate family.asparagine | Gra.1614.2.S1_x_at | Gra.3247 | -7.7225747 | CO127533 | AT3G47340.1 | glutamine-dependent asparagine synthase 1 |
|  |  | 13.1.3.1 | amino acid metabolism.synthesis.aspartate family.asparagine | Gra.2231.1.S1_s_at | Gra.2231 | -21.865326 | CO089843 | AT3G47340.1 | glutamine-dependent asparagine synthase 1 |
|  |  | 13.1.3.1.1 | amino acid metabolism.synthesis.aspartate family.asparagine.asparagine synthetase | Ghi.10655.1.S1_s_at | Ghi.10655 | -10.458097 | DN780602 | AT3G47340.1 | glutamine-dependent asparagine synthase 1 |
|  |  | 13.1.3.1.1 | amino acid metabolism.synthesis.aspartate family.asparagine.asparagine synthetase | Ghi.1436.1.S1_at | Ghi.10655 | -7.506804 | DN799898 | AT3G47340.1 | glutamine-dependent asparagine synthase 1 |
|  |  | 13.1.3.1.1 | amino acid metabolism.synthesis.aspartate family.asparagine.asparagine synthetase | Gra.1559.1.A1_at | Gra.2231 | -22.42489 | CO123242 | AT3G47340.1 | glutamine-dependent asparagine synthase 1 |
|  |  | 13.1.5.3.1 | amino acid metabolism.synthesis.serine-glycine-cysteine group.cysteine.OASTL | Gra.911.1.A1_s_at | Gra.911 | -3.9031658 | CO121286 | AT3G61440.1 | BSAS3 |
|  |  | 13.1.5.3.1 | amino acid metabolism.synthesis.serine-glycine-cysteine group.cysteine.OASTL | Ghi.5401.1.S1_s_at | Ghi.5401 | -3.8220873 | DT047881 | AT3G61440.1 | BSAS3 |

|  |  | 13.2.3.5 | amino acid metabolism.degradation.aspartate family.lysine | GraAffx.14609.1.A1_s_at | | -3.66268 | CO127185 | AT4G33150.3 | lysine-ketoglutarate reductase/saccharopine dehydrogenase bifunctional enzyme |
| --- | --- | --- | --- | --- | --- | --- | --- | --- | --- |
| **15** | Metal handling | 15.2 | metal handling.binding, chelation and storage | Ghi.1586.1.S1_at | Ghi.17925 | -4.195183 | DN760004 | AT2G36950.1 | Heavy metal transport/detoxification superfamily protein |
| **16** | Secondary metabolism | 16.1.4.6 | secondary metabolism.isoprenoids.carotenoids.carotenoid beta ring hydroxylase | GhiAffx.17151.1.S1_at | Ghi.19974 | -3.6860385 | DW243562.1 | AT5G52570.1 | beta-carotene hydroxylase 2 |
|  |  | 16.1.5 | secondary metabolism.isoprenoids.terpenoids | GarAffx.37202.1.S1_x_at | | -3.5024858 | U23205.1 | AT5G23960.2 | terpene synthase 21 |
|  |  | 16.2 | secondary metabolism.phenylpropanoids | GhiAffx.15490.1.S1_at | Ghi.18655 | -3.6327517 | DW237402.1 | AT2G22570.1 | nicotinamidase 1 |
|  |  | 16.2 | secondary metabolism.phenylpropanoids | Ghi.5521.1.A1_s_at | Ghi.5521 | -8.330377 | DT047436 | AT5G01210.1 | HXXXD-type acyl-transferase family protein |
|  |  | 16.2.1.6 | secondary metabolism.phenylpropanoids.lignin biosynthesis.CCoAOMT | Ghi.4349.1.A1_at | Ghi.4349 | -5.01652 | DT054063 | AT1G67980.2 | caffeoyl-CoA 3-O-methyltransferase |
|  |  | 16.4.1 | secondary metabolism.N misc.alkaloid-like | Ghi.8033.1.S1_s_at | Ghi.16611 | -8.29359 | DQ122187.1 | AT2G20340.1 | Pyridoxal phosphate (PLP)-dependent transferases superfamily protein |
|  |  | 16.8.1.21 | secondary metabolism.flavonoids.anthocyanins.anthocyanin 5-aromatic acyltransferase | Ghi.1935.1.S1_at | Ghi.1935 | -7.5990176 | DV848993 | AT3G29590.1 | HXXXD-type acyl-transferase family protein |
|  |  | 16.8.4 | secondary metabolism.flavonoids.flavonols | GhiAffx.1402.1.S1_at | Ghi.14499 | -3.4361563 | DW231451.1 | AT1G15550.1 | gibberellin 3-oxidase 1 |
| **17** | Hormone metabolism | 17.2.3 | hormone metabolism.auxin.induced-regulated-responsive-activated | Ghi.10753.1.S1_at | Ghi.10753 | -5.6141815 | DN760125 | AT4G27450.1 | Aluminium induced protein with YGL and LRDR motifs |
|  |  | 17.2.3 | hormone metabolism.auxin.induced-regulated-responsive-activated | GhiAffx.6062.1.S1_at | Ghi.15960 | 3.1669397 | DW495992.1 | AT5G20820.1 | SAUR-like auxin-responsive protein family |
|  |  | 17.2.3 | hormone metabolism.auxin.induced-regulated-responsive-activated | GhiAffx.28716.1.S1_at | Ghi.15689 | -3.0640888 | DW512426.1 | AT4G00880.1 | SAUR-like auxin-responsive protein family |
|  |  | 17.2.3 | hormone metabolism.auxin.induced-regulated-responsive-activated | Gra.275.1.S1_s_at | Gra.275 | -3.2121694 | CO090219 | AT1G60710.1 | NAD(P)-linked oxidoreductase superfamily protein |
|  |  | 17.2.3 | hormone metabolism.auxin.induced-regulated-responsive-activated | Ghi.5305.1.A1_at | Ghi.5305 | -3.2528284 | DT048241 | AT5G25890.1 | indole-3-acetic acid inducible 28 |
|  |  | 17.5.1 | hormone metabolism.ethylene.synthesis-degradation | Ghi.798.1.S1_s_at | Ghi.16386 | -4.1597486 | DQ116444.1 | AT1G05010.1 | ethylene-forming enzyme |
|  |  | 17.5.1 | hormone metabolism.ethylene.synthesis-degradation | Gra.2141.1.S1_s_at | | -39.88526 | CO123471 | AT1G05010.1 | ethylene-forming enzyme |
|  |  | 17.5.1 | hormone metabolism.ethylene.synthesis-degradation | GhiAffx.1402.1.S1_at | Ghi.14499 | -3.4361563 | DW231451.1 | AT1G15550.1 | gibberellin 3-oxidase 1 |

|  |  | 17.5.1 | hormone metabolism.ethylene.synthesis-degradation | Ghi.6953.1.S1_s_at | Ghi.16374 | -15.015603 | DQ116442.1 | AT1G05010.1 | ethylene-forming enzyme |
| --- | --- | --- | --- | --- | --- | --- | --- | --- | --- |
|  |  | 17.5.1 | hormone metabolism.ethylene.synthesis-degradation | Ghi.8023.1.S1_at | Ghi.16693 | -39.720764 | DQ116443.1 | AT1G12010.1 | 2-oxoglutarate (2OG) and Fe(II)-dependent oxygenase superfamily protein |
|  |  | 17.5.2 | hormone metabolism.ethylene.signal transduction | GhiAffx.28739.1.S1_s_at | Ghi.15180 | -4.0409465 | DW502086.1 | AT5G47220.1 | ethylene responsive element binding factor 2 |
|  |  | 17.5.2 | hormone metabolism.ethylene.signal transduction | Ghi.5775.1.S1_s_at | Ghi.5775 | -5.514077 | DT455881 | AT3G23150.1 | Signal transduction histidine kinase, hybrid-type, ethylene sensor |
|  |  | 17.5.2 | hormone metabolism.ethylene.signal transduction | Ghi.10747.1.S1_at | Ghi.10747 | -4.822069 | DV850132 | AT4G17500.1 | ethylene responsive element binding factor 1 |
|  |  | 17.5.2 | hormone metabolism.ethylene.signal transduction | GhiAffx.7865.1.S1_at | | -4.5940685 | DW503266.1 | AT5G61590.1 | Integrase-type DNA-binding superfamily protein |
|  |  | 17.5.3 | hormone metabolism.ethylene.induced-regulated-responsive-activated | GhiAffx.61605.1.S1_s_at | Ghi.16587 | -3.3367488 | DN800946 | AT3G62550.1 | Adenine nucleotide alpha hydrolases-like superfamily protein |
|  |  | 17.6.2 | hormone metabolism.gibberelin.signal transduction | Gra.1544.1.A1_s_at | | -5.5872493 | CO091149 | AT3G63010.1 | alpha/beta-Hydrolases superfamily protein |
| **20** | Stress | 20.1 | stress.biotic | GhiAffx.49772.1.A1_at | | 9.510919 | DT464025 | AT5G01890.1 | Leucine-rich receptor-like protein kinase family protein |
|  |  | 20.1 | stress.biotic | Ghi.8400.1.S1_s_at | Ghi.9142 | -7.734738 | DN827419 | AT2G15130.1 | Plant basic secretory protein (BSP) family protein |
|  |  | 20.1 | stress.biotic | GhiAffx.25472.1.A1_s_at | | -5.6117935 | DW516477.1 | AT3G04720.1 | pathogenesis-related 4 |
|  |  | 20.1.7 | stress.biotic.PR-proteins | Ghi.3370.1.A1_at | Ghi.3370 | -45.29446 | DT463939 | AT1G33590.1 | Leucine-rich repeat (LRR) family protein |
|  |  | 20.1.7 | stress.biotic.PR-proteins | Ghi.490.1.S1_s_at | Ghi.490 | -14.921879 | DT465033 | AT1G33590.1 | Leucine-rich repeat (LRR) family protein |
|  |  | 20.1.7 | stress.biotic.PR-proteins | GhiAffx.53241.1.S1_at | Ghi.12870 | -7.7497573 | DW518118.1 | AT1G58170.1 | Disease resistance-responsive (dirigent-like protein) family protein |
|  |  | 20.1.7 | stress.biotic.PR-proteins | Ghi.3370.1.S1_s_at | Ghi.3370 | -4.8305883 | DT466783 | AT1G33590.1 | Leucine-rich repeat (LRR) family protein |
|  |  | 20.1.7 | stress.biotic.PR-proteins | GhiAffx.8053.1.A1_at | | -21.132198 | DW518810.1 | AT1G64160.1 | Disease resistance-responsive (dirigent-like protein) family protein |
|  |  | 20.1.7.6.1 | stress.biotic.PR-proteins.proteinase inhibitors.trypsin inhibitor | GhiAffx.61299.1.S1_at | Ghi.18695 | -3.6133204 | DW508705.1 | AT1G17860.1 | Kunitz family trypsin and protease inhibitor protein |
|  |  | 20.2.1 | stress.abiotic.heat | GhiAffx.16191.1.S1_at | Ghi.11788 | -4.588662 | DW507100.1 | AT2G29500.1 | HSP20-like chaperones superfamily protein |

|  |  | 20.2.1 | stress.abiotic.heat | Ghi.6780.1.S1_s_at | Ghi.6780 | -15.165595 | CA993199 | AT2G17880.1 | Chaperone DnaJ-domain superfamily protein |
| --- | --- | --- | --- | --- | --- | --- | --- | --- | --- |
|  |  | 20.2.99 | stress.abiotic.unspecified | Ghi.6410.1.S1_at | Ghi.6410 | -4.438521 | CD486551 | AT1G70830.3 | MLP-like protein 28 |
|  |  | 20.2.99 | stress.abiotic.unspecified | GbaAffx.207.1.S1_s_at | | -4.109701 | AY560551.1 | |  |
|  |  | 20.2.99 | stress.abiotic.unspecified | Ghi.7942.2.S1_a_at | Ghi.7942 | -9.429709 | DT467978 | AT2G21620.1 | Adenine nucleotide alpha hydrolases-like superfamily protein |
|  |  | 20.2.99 | stress.abiotic.unspecified | Ghi.10676.1.S1_s_at | Ghi.10676 | -4.287772 | DT567365 | AT1G01360.1 | regulatory component of ABA receptor 1 |
| **22** | Polyamine metabolism | 22.1.6 | polyamine metabolism.synthesis.spermidine synthase | GhiAffx.2527.1.S1_s_at | Ghi.13939 | -168.97514 | DW497370.1 | AT5G53120.6 | spermidine synthase 3 |
| **23** | Nucleotide metabolism | 23.2.1.3 | nucleotide metabolism.degradation.pyrimidine.dihydrouracil dehydrogenase | Gra.2847.1.A1_s_at | Gra.2847 | -3.293587 | CO071816 | AT3G17810.1 | pyrimidine 1 |
| **24** | Biodegradation of Xenobiotics | 24 | Biodegradation of Xenobiotics | Gra.1544.1.A1_s_at | | -5.5872493 | CO091149 | AT3G63010.1 | alpha/beta-Hydrolases superfamily protein |
|  |  | 24 | Biodegradation of Xenobiotics | Gra.2150.1.S1_s_at | Gra.2150 | -4.206167 | CO085918 | AT5G06570.2 | alpha/beta-Hydrolases superfamily protein |
|  |  | 24.2 | Biodegradation of Xenobiotics.lactoylglutathione lyase | GhiAffx.26021.1.S1_at | Ghi.16198 | -3.0363617 | DW232275.1 | AT1G15380.2 | Lactoylglutathione lyase / glyoxalase I family protein |
| **26** | Misc | 26.2 | misc.UDP glucosyl and glucoronyl transferases | Ghi.6822.1.A1_s_at | Ghi.6822 | -7.3133745 | CA993006 | AT2G36750.1 | UDP-glucosyl transferase 73C1 |
|  |  | 26.2 | misc.UDP glucosyl and glucoronyl transferases | GhiAffx.31355.1.S1_s_at | Ghi.13637 | -3.9368427 | DT462536 | AT4G34138.1 | UDP-glucosyl transferase 73B1 |
|  |  | 26.3.2 | misc.gluco-, galacto- and mannosidases.beta-galactosidase | Gra.2314.1.S1_at | Gra.2314 | -6.476485 | CO126415 | AT3G13750.1 | beta galactosidase 1 |
|  |  | 26.3.2 | misc.gluco-, galacto- and mannosidases.beta-galactosidase | Gra.2459.1.A1_s_at | Gra.3587 | -6.184939 | CO128080 | AT3G52840.1 | beta-galactosidase 2 |
|  |  | 26.3.2 | misc.gluco-, galacto- and mannosidases.beta-galactosidase | Gra.2056.1.A1_s_at | | -5.0454865 | CO121156 | AT2G16730.1 | glycosyl hydrolase family 35 protein |
|  |  | 26.9 | misc.glutathione S transferases | Ghi.1016.4.S1_s_at | Ghi.10821 | -7.797561 | DT468576 | AT3G09270.1 | glutathione S-transferase TAU 8 |
|  |  | 26.9 | misc.glutathione S transferases | Ghi.779.1.S1_at | Ghi.779 | -3.0192256 | DN800052 | AT1G10370.1 | Glutathione S-transferase family protein |
|  |  | 26.1 | misc.cytochrome P450 | Ghi.6547.1.S1_s_at | | -3.5325873 | DR458804 | AT4G12320.1 | cytochrome P450, family 706, subfamily A, polypeptide 6 |
|  |  | 26.1 | misc.cytochrome P450 | GhiAffx.22064.1.S1_at | Ghi.12850 | -6.3178463 | DW498676.1 | AT3G14680.1 | cytochrome P450, family 72, subfamily A, polypeptide 14 |
|  |  | 26.12 | misc.peroxidases | Ghi.8105.1.A1_s_at | Ghi.8105 | -7.123931 | AF488305.1 | AT5G06720.1 | peroxidase 2 |

|  |  | 26.12 | misc.peroxidases | Ghi.7950.1.S1_at | Ghi.16267 | -81.1664 | AY366083.1 | AT5G06720.1 | peroxidase 2 |
| --- | --- | --- | --- | --- | --- | --- | --- | --- | --- |
|  |  | 26.12 | misc.peroxidases | Ghi.1043.4.S1_at | Ghi.17859 | -8.142763 | DT463348 | AT5G05340.1 | Peroxidase superfamily protein |
|  |  | 26.12 | misc.peroxidases | Ghi.8110.1.S1_at | Ghi.8110 | -4.90623 | AY311597.1 | AT5G05340.1 | Peroxidase superfamily protein |
|  |  | 26.18 | misc.invertase/pectin methylesterase inhibitor family protein | Ghi.1552.1.S1_s_at | Ghi.1552 | -18.200262 | DN779868 | AT1G47960.1 | cell wall / vacuolar inhibitor of fructosidase 1 |
|  |  | 26.21 | misc.protease inhibitor/seed storage/lipid transfer protein (LTP) family protein | Gra.378.1.A1_s_at | Gra.378 | -3.1366498 | CO085861 | AT3G53980.2 | Bifunctional inhibitor/lipid-transfer protein/seed storage 2S albumin superfamily protein |
|  |  | 26.21 | misc.protease inhibitor/seed storage/lipid transfer protein (LTP) family protein | Ghi.10795.1.S1_s_at | Ghi.6472 | 3.265911 | CD486563 | AT2G45180.1 | Bifunctional inhibitor/lipid-transfer protein/seed storage 2S albumin superfamily protein |
|  |  | 26.22 | misc.short chain dehydrogenase/reductase (SDR) | Ghi.9176.3.A1_at | Ghi.9176 | -3.419338 | DT464586 | AT3G51680.1 | NAD(P)-binding Rossmann-fold superfamily protein |
|  |  | 26.22 | misc.short chain dehydrogenase/reductase (SDR) | Ghi.4.1.A1_at | Ghi.4 | -7.606558 | CK987701 | AT1G52340.1 | NAD(P)-binding Rossmann-fold superfamily protein |
| **27** | RNA | 27.1.19 | RNA.processing.ribonucleases | GarAffx.19282.1.S1_s_at | | -3.9836557 | AF416652.1 | AT1G24020.2 | MLP-like protein 423 |
|  |  | 27.1.19 | RNA.processing.ribonucleases | Ghi.6485.1.S1_s_at | | -3.8291993 | CD485617 |  |  |
|  |  | 27.1.19 | RNA.processing.ribonucleases | GarAffx.19282.1.S1_x_at | | -3.390929 | AF416652.1 | AT1G24020.2 | MLP-like protein 423 |
|  |  | 27.1.19 | RNA.processing.ribonucleases | Ghi.6449.1.A1_at | Ghi.6449 | -7.0055966 | CK640602 |  |  |
|  |  | 27.1.19 | RNA.processing.ribonucleases | Ghi.6523.1.S1_s_at | Ghi.6523 | -4.638748 | AF305064.1 | |  |
|  |  | 27.1.19 | RNA.processing.ribonucleases | Ghi.4737.1.A1_at | Ghi.4737 | -6.5215945 | DT051082 | AT1G26820.1 | ribonuclease 3 |
|  |  | 27.1.19 | RNA.processing.ribonucleases | GbaAffx.201.1.S1_s_at | | -4.8380117 | AY560553.1 | |  |
|  |  | 27.3.3 | RNA.regulation of transcription.AP2/EREBP, APETALA2/Ethylene-responsive element binding protein family | GhiAffx.28739.1.S1_s_at | Ghi.15180 | -4.0409465 | DW502086.1 | AT5G47220.1 | ethylene responsive element binding factor 2 |
|  |  | 27.3.3 | RNA.regulation of transcription.AP2/EREBP, APETALA2/Ethylene-responsive element binding protein family | GhiAffx.30941.1.S1_s_at | Ghi.13511 | -3.2078433 | DW482613.1 | AT1G13260.1 | related to ABI3/VP1 1 |
|  |  | 27.3.3 | RNA.regulation of transcription.AP2/EREBP, APETALA2/Ethylene-responsive element binding protein family | GbaAffx.196.1.A1_s_at | | -7.6331277 | AY572462.1 | AT3G16770.1 | ethylene-responsive element binding protein |
|  |  | 27.3.3 | RNA.regulation of transcription.AP2/EREBP, APETALA2/Ethylene-responsive element binding protein family | Ghi.7874.1.S1_s_at | Ghi.16277 /// Ghi.7874 | -10.277513 | AY962572.1 | AT3G16770.1 | ethylene-responsive element binding protein |
|  |  | 27.3.6 | RNA.regulation of transcription.bHLH,Basic Helix-Loop-Helix family | Ghi.1326.1.S1_s_at | Ghi.1326 | -5.472375 | DR463721 | AT3G25710.1 | basic helix-loop-helix 32 |

|  |  | 27.3.11 | RNA.regulation of transcription.C2H2 zinc finger family | Ghi.807.1.S1_s_at | Ghi.17797 | -3.851732 | DT465871 | AT1G27730.1 | salt tolerance zinc finger |
| --- | --- | --- | --- | --- | --- | --- | --- | --- | --- |
|  |  | 27.3.22 | RNA.regulation of transcription.HB,Homeobox transcription factor family | Ghi.9281.1.A1_s_at | Ghi.17961 | -3.1192684 | DT047152 | AT4G36740.1 | homeobox protein 40 |
|  |  | 27.3.22 | RNA.regulation of transcription.HB,Homeobox transcription factor family | Ghi.3578.1.S1_s_at | Ghi.3578 | -10.348414 | DT567472 | AT4G36740.1 | homeobox protein 40 |
|  |  | 27.3.25 | RNA.regulation of transcription.MYB domain transcription factor family | GhiAffx.48583.1.S1_at | | -6.144695 | AI055122 | AT3G06490.1 | myb domain protein 108 |
|  |  | 27.3.27 | RNA.regulation of transcription.NAC domain transcription factor family | Ghi.3446.1.A1_at | Ghi.3446 | -5.4198785 | DT462755 | AT4G28530.1 | NAC domain containing protein 74 |
|  |  | 27.3.32 | RNA.regulation of transcription.WRKY domain transcription factor family | GhiAffx.1859.1.S1_at | | -7.2392125 | DT468306 | AT5G13080.1 | WRKY DNA-binding protein 75 |
|  |  | 27.3.32 | RNA.regulation of transcription.WRKY domain transcription factor family | Ghi.9193.2.A1_at | Ghi.9193 | -12.23719 | DT469110 | AT3G56400.1 | WRKY DNA-binding protein 70 |
|  |  | 27.3.32 | RNA.regulation of transcription.WRKY domain transcription factor family | GhiAffx.30199.1.S1_at | Ghi.15715 | -3.6616116 | DW506814.1 | AT2G47260.1 | WRKY DNA-binding protein 23 |
|  |  | 27.3.40 | RNA.regulation of transcription.Aux/IAA family | Ghi.6543.1.S1_s_at | Ghi.6543 | -8.538899 | DN780646 | AT4G14550.1 | indole-3-acetic acid inducible 14 |
|  |  | 27.3.40 | RNA.regulation of transcription.Aux/IAA family | GhiAffx.6395.1.S1_s_at | Ghi.13987 | -3.1220052 | DW484802.1 | AT4G14550.1 | indole-3-acetic acid inducible 14 |
|  |  | 27.3.40 | RNA.regulation of transcription.Aux/IAA family | Ghi.5305.1.A1_at | Ghi.5305 | -3.2528284 | DT048241 | AT5G25890.1 | indole-3-acetic acid inducible 28 |
|  |  | 27.3.99 | RNA.regulation of transcription.unclassified | Ghi.9243.2.A1_s_at | Ghi.9243 | -7.4341016 | DR452409 | AT4G17900.1 | PLATZ transcription factor family protein |
|  |  | 27.3.99 | RNA.regulation of transcription.unclassified | Ghi.6262.1.S1_x_at | | -3.3666704 | DT464008 | AT4G17900.1 | PLATZ transcription factor family protein |
| **28** | DNA | 28.1 | DNA.synthesis/chromatin structure | GhiAffx.21558.1.S1_s_at | | -4.3981233 | DW495821.1 | AT1G11190.1 | bifunctional nuclease I |
|  |  | 28.1 | DNA.synthesis/chromatin structure | Ghi.9152.1.S1_at | Ghi.9152 | -10.333591 | DT462541 | AT3G18950.1 | Transducin/WD40 repeat-like superfamily protein |
| **29** | Protein | 29.2.3 | protein.synthesis.initiation | Ghi.5887.1.S1_at | Ghi.5887 | -7.941222 | CO499594 | AT5G54940.2 | Translation initiation factor SUI1 family protein |
|  |  | 29.4 | protein.postranslational modification | GhiAffx.60835.1.S1_at | Ghi.12975 | -3.2344124 | DW506319.1 | AT5G58380.1 | SOS3-interacting protein 1 |
|  |  | 29.4 | protein.postranslational modification | Ghi.6548.1.S1_s_at | Ghi.16406 | -3.5500994 | AY207316.1 | AT5G55560.1 | Protein kinase superfamily protein |
|  |  | 29.4 | protein.postranslational modification | Ghi.6088.1.S1_s_at | Ghi.6088 | -6.3369107 | DT466983 | AT3G45640.1 | mitogen-activated protein kinase 3 |
|  |  | 29.5.1 | protein.degradation.subtilases | Ghi.967.1.S1_s_at | Ghi.967 | -4.3808484 | AI728289 | AT5G67090.1 | Subtilisin-like serine endopeptidase family protein |

|  |  | 29.5.4 | protein.degradation.aspartate protease | Ghi.7891.1.S1_s_at | Ghi.7891 | -6.325849 | DT462224 | AT1G03220.1 | Eukaryotic aspartyl protease family protein |
| --- | --- | --- | --- | --- | --- | --- | --- | --- | --- |
|  |  | 29.5.11.4.2 | protein.degradation.ubiquitin.E3.RING | GhiAffx.429.1.A1_at | | -3.1865566 | DW233845.1 | AT4G37890.2 | Zinc finger (C3HC4-type RING finger) family protein |
|  |  | 29.5.11.4.2 | protein.degradation.ubiquitin.E3.RING | GhiAffx.36084.1.A1_at | Ghi.21982 | -3.337532 | DW516195.1 | AT2G04240.2 | RING/U-box superfamily protein |
|  |  | 29.5.11.4.2 | protein.degradation.ubiquitin.E3.RING | GhiAffx.9522.1.A1_s_at | Ghi.19273 | -3.7814333 | DT049461 | AT3G25030.2 | RING/U-box superfamily protein |
|  |  | 29.5.11.4.3.2 | protein.degradation.ubiquitin.E3.SCF.FBOX | GhiAffx.23896.1.S1_at | Ghi.11550 | -4.7215858 | DW497980.1 | AT4G21510.1 | F-box family protein |
| **30** | Signalling | 30.2.2 | signalling.receptor kinases.leucine rich repeat II | Ghi.44.1.A1_at | Ghi.44 | -3.345396 | DR463380 | AT5G10290.1 | leucine-rich repeat transmembrane protein kinase family protein |
|  |  | 30.2.11 | signalling.receptor kinases.leucine rich repeat XI | GbaAffx.197.1.S1_s_at | | -4.1984053 | AY279356.1 | AT5G06860.1 | polygalacturonase inhibiting protein 1 |
|  |  | 30.2.17 | signalling.receptor kinases.DUF 26 | GhiAffx.3185.1.S1_at | Ghi.17263 | -76.70687 | DW514553.1 | AT1G78860.1 | D-mannose binding lectin protein with Apple-like carbohydrate-binding domain |
|  |  | 30.6 | signalling.MAP kinases | Ghi.6088.2.A1_s_at | Ghi.6088 | -10.754808 | DV849489 | AT3G45640.1 | mitogen-activated protein kinase 3 |
|  |  | 30.6 | signalling.MAP kinases | Ghi.5022.4.A1_s_at | Ghi.17602 | -5.890732 | DT049392 | AT1G73500.1 | MAP kinase kinase 9 |
| **31** | Cell | 31.1 | cell.organisation | Ghi.468.1.A1_s_at | Ghi.16435 | -3.8680217 | AY189970.1 | AT2G19770.1 | profilin 5 |
|  |  | 31.1 | cell.organisation | GhiAffx.33535.1.S1_at | | -3.0148811 | AI727184 | AT2G16700.2 | actin depolymerizing factor 5 |
|  |  | 31.1 | cell.organisation | Ghi.8448.1.S1_x_at | Ghi.8448 | -11.607274 | AF521240.1 | AT5G12250.1 | beta-6 tubulin |
| **33** | Development | 33.1 | development.storage proteins | GhiAffx.19570.1.S1_at | Ghi.14271 | -5.245414 | DW489734.1 | AT2G26560.1 | phospholipase A 2A |
|  |  | 33.2 | development.late embryogenesis abundant | Ghi.6722.1.S1_s_at | Ghi.16396 | -5.302019 | M88322.1 | AT1G01470.1 | Late embryogenesis abundant protein |
|  |  | 33.99 | development.unspecified | Ghi.7907.1.S1_s_at | Ghi.7907 | -6.206703 | AI055500 | AT4G27410.2 | NAC (No Apical Meristem) domain transcriptional regulator superfamily protein |
|  |  | 33.99 | development.unspecified | Ghi.9328.1.S1_s_at | Ghi.4821 | -5.254816 | DT048550 | AT5G13180.1 | NAC domain containing protein 83 |
|  |  | 33.99 | development.unspecified | Gra.2844.1.S1_s_at | Gra.17 | -3.043563 | CO087535 | AT5G50790.1 | Nodulin MtN3 family protein |
|  |  | 33.99 | development.unspecified | Ghi.5307.1.A1_at | Ghi.5307 | -6.6096444 | DT048232 | AT3G48140.1 | B12D protein |
|  |  | 33.99 | development.unspecified | Ghi.3446.1.A1_at | Ghi.3446 | -5.4198785 | DT462755 | AT4G28530.1 | NAC domain containing protein 74 |
|  |  | 33.99 | development.unspecified | Gra.2669.1.A1_at | Gra.2669 | 3.0801332 | CO085938 | AT1G70260.1 | nodulin MtN21 /EamA-like transporter family protein |

|  |  | 33.99 | development.unspecified | Ghi.6538.1.S1_at | Ghi.6538 | -14.719816 | CD485949 | AT3G04070.1 | NAC domain containing protein 47 |
| --- | --- | --- | --- | --- | --- | --- | --- | --- | --- |
|  |  | 33.99 | development.unspecified | Ghi.3264.1.S1_s_at | Ghi.3264 | -5.772931 | DT466083 | AT1G01720.1 | NAC (No Apical Meristem) domain transcriptional regulator superfamily protein |
| **34** | Transport | 34.2 | transport.sugars | GhiAffx.31391.1.S1_s_at | | -8.354315 | DW481920.1 | AT3G18830.1 | polyol/monosaccharide transporter 5 |
|  |  | 34.8 | transport.metabolite transporters at the envelope membrane | GhiAffx.42790.1.S1_at | | 3.8775904 | DW509815.1 | AT3G01550.1 | phosphoenolpyruvate (pep)/phosphate translocator 2 |
|  |  | 34.13 | transport.peptides and oligopeptides | Ghi.3135.1.S1_at | Ghi.3135 | -7.021998 | DT469074 | AT1G32450.1 | nitrate transporter 1.5 |
|  |  | 34.13 | transport.peptides and oligopeptides | Ghi.1847.1.S1_at | Ghi.1847 | -7.26273 | DV850045 | AT5G46050.1 | peptide transporter 3 |
|  |  | 34.14 | transport.unspecified cations | GraAffx.17697.1.A1_s_at | | -3.4341981 | CO086224 | AT5G47560.1 | tonoplast dicarboxylate transporter |
|  |  | 34.16 | transport.ABC transporters and multidrug resistance systems | Ghi.7279.1.S1_at | Ghi.7279 | -6.3336034 | DR455241 | AT2G26910.1 | pleiotropic drug resistance 4 |
|  |  | 34.16 | transport.ABC transporters and multidrug resistance systems | GhiAffx.2060.1.S1_at | | -4.663229 | DW477541.1 | AT1G15210.1 | pleiotropic drug resistance 7 |
|  |  | 34.16 | transport.ABC transporters and multidrug resistance systems | Ghi.9146.2.S1_s_at | Ghi.24492 | -6.696793 | DT463838 | AT5G13740.1 | zinc induced facilitator 1 |
|  |  | 34.16 | transport.ABC transporters and multidrug resistance systems | Ghi.9146.1.S1_s_at | Ghi.9146 | -15.876558 | DT463593 | AT5G13740.1 | zinc induced facilitator 1 |
|  |  | 34.19.1 | transport.Major Intrinsic Proteins.PIP | GhiAffx.10920.2.S1_at | Ghi.16091 | -3.8752277 | DW237583.1 | AT2G37170.1 | 2 |
|  |  | 34.99 | transport.misc | GraAffx.1410.1.S1_at | | -11.052195 | CO125821 | AT5G65980.1 | Auxin efflux carrier family protein |
| **35** | Not assigned | 35.2 | not assigned.unknown | Ghi.4960.2.A1_s_at | Ghi.4960 | -5.676947 | DT046397 | AT4G27740.1 | Yippee family putative zinc-binding protein |
|  |  | 35.2 | not assigned.unknown | Ghi.3049.1.A1_at | Ghi.3049 | -3.0012093 | DR456375 | AT5G02230.2 | Haloacid dehalogenase-like hydrolase (HAD) superfamily protein |
|  |  | 35.2 | not assigned.unknown | Ghi.6067.1.A1_at | Ghi.6067 | -20.803682 | DT048703 | AT3G22600.1 | Bifunctional inhibitor/lipid-transfer protein/seed storage 2S albumin superfamily protein |
|  |  | 35.2 | not assigned.unknown | Ghi.8389.1.S1_a_at | Ghi.8389 | -10.248608 | CO498953 | AT4G15610.1 | Uncharacterised protein family (UPF0497) |
|  |  | 35.2 | not assigned.unknown | Ghi.5587.1.S1_at | Ghi.5587 | -4.5403953 | CA993040 |  |  |
|  |  | 35.2 | not assigned.unknown | Ghi.341.1.S1_x_at | Ghi.341 | -6.176137 | DR460630 |  |  |
|  |  | 35.2 | not assigned.unknown | GhiAffx.15571.1.S1_a_at | | -4.0165467 | DW505599.1 | AT1G67920.1 | unknown protein |
|  |  | 35.2 | not assigned.unknown | GhiAffx.18573.1.S1_s_at | Ghi.1313 | -6.32572 | DW500069.1 | AT1G15400.3 | unknown protein |

|  |  | 35.2 | not assigned.unknown | Ghi.592.1.S1_at | Ghi.592 | -4.1952853 | DR457234 |  |  |
| --- | --- | --- | --- | --- | --- | --- | --- | --- | --- |
|  |  | 35.2 | not assigned.unknown | GhiAffx.59002.1.S1_at | Ghi.14714 | -4.7086577 | DW497255.1 | AT1G19530.1 | unknown protein |
|  |  | 35.2 | not assigned.unknown | Ghi.3480.1.S1_at | Ghi.3480 | -4.8646755 | DN760204 | |  |
|  |  | 35.2 | not assigned.unknown | Ghi.8424.1.A1_at | Ghi.8424 | -4.822119 | CO490790 | |  |
|  |  | 35.2 | not assigned.unknown | Ghi.6632.1.A1_s_at | Ghi.2362 | -7.6711617 | DT461730 | AT1G07150.2 | mitogen-activated protein kinase kinase kinase 13 |
|  |  | 35.2 | not assigned.unknown | Ghi.5512.1.A1_s_at | Ghi.5512 | -4.5000496 | DT047390 |  |  |
|  |  | 35.2 | not assigned.unknown | Ghi.7171.1.A1_at | Ghi.7171 | -7.735075 | DT461933 |  |  |
|  |  | 35.2 | not assigned.unknown | Ghi.5997.1.A1_at | Ghi.5997 | -6.278993 | CO492935 | AT1G29050.1 | TRICHOME BIREFRINGENCE-LIKE 38 |
|  |  | 35.2 | not assigned.unknown | Ghi.812.1.S1_at | Ghi.812 | -4.1031847 | DT462925 | AT5G60680.1 | Protein of unknown function, DUF584 |
|  |  | 35.2 | not assigned.unknown | Ghi.7907.2.A1_at | Ghi.7907 | -9.67557 | DT463334 |  |  |
|  |  | 35.2 | not assigned.unknown | Ghi.2608.2.A1_at | Ghi.2608 | -5.8584695 | DT463212 | AT3G55840.1 | Hs1pro-1 protein |
|  |  | 35.2 | not assigned.unknown | GhiAffx.25661.1.S1_at | Ghi.11202 | -5.122869 | DW517516.1 | AT3G27880.1 | Protein of unknown function (DUF1645) |
|  |  | 35.2 | not assigned.unknown | Ghi.6417.3.S1_s_at | Ghi.6417 | -5.8997707 | DN826313 | |  |
|  |  | 35.2 | not assigned.unknown | Ghi.4960.1.A1_s_at | Ghi.4960 | -5.6390157 | DT051751 | AT4G27740.1 | Yippee family putative zinc-binding protein |
|  |  | 35.2 | not assigned.unknown | Ghi.9152.2.A1_at | Ghi.17824 | -14.702347 | DT463871 |  |  |
|  |  | 35.2 | not assigned.unknown | Ghi.6692.1.S1_s_at | Ghi.6692 | -3.0784924 | CA993159 | AT1G67920.1 | unknown protein |
|  |  | 35.2 | not assigned.unknown | Ghi.8381.1.S1_s_at | Ghi.8381 | -7.79501 | DT047422 |  |  |
|  |  | 35.2 | not assigned.unknown | GhiAffx.42158.1.S1_at | Ghi.18276 | -4.3744025 | DW233814.1 | |  |
|  |  | 35.2 | not assigned.unknown | Gra.998.2.S1_s_at | Gra.998 | -3.354782 | CO089323 | AT2G27830.1 | unknown protein |
|  |  | 35.2 | not assigned.unknown | Ghi.6472.1.A1_s_at | Ghi.6472 | 3.0664685 | CD485893 | AT2G45180.1 | Bifunctional inhibitor/lipid-transfer protein/seed storage 2S albumin superfamily protein |
|  |  | 35.2 | not assigned.unknown | GhiAffx.5935.2.S1_s_at | Ghi.9213 | -7.227638 | DW235907.1 | AT3G55840.1 | Hs1pro-1 protein |
|  |  | 35.2 | not assigned.unknown | GhiAffx.33994.1.A1_s_at | | -7.4532385 | DW515886.1 | AT3G04070.2 | NAC domain containing protein 47 |
|  |  | 35.2 | not assigned.unknown | Ghi.6102.1.S1_at | Ghi.6102 | -3.5751066 | DR458734 |  |  |
|  |  | 35.2 | not assigned.unknown | Ghi.1037.2.S1_x_at | Ghi.8296 | -4.6102133 | CA993067 | AT3G15353.2 | metallothionein 3 |
|  |  | 35.2 | not assigned.unknown | Ghi.1955.1.S1_at | Ghi.1955 | -5.3867755 | DV848730 | AT4G31240.2 | protein kinase C-like zinc finger protein |
|  |  | 35.2 | not assigned.unknown | Ghi.5626.1.S1_s_at | Ghi.5626 | -4.467655 | DT466989 | AT4G01500.1 | AP2/B3-like transcriptional factor family protein |
|  |  | 35.2 | not assigned.unknown | Ghi.8342.1.S1_at | Ghi.8342 | -3.905866 | DV849242 | |  |

|  |  | 35.2 | not assigned.unknown | GhiAffx.44018.1.S1_at | Ghi.15557 | -12.242164 | DW502867.1 | AT1G30260.1 | BEST Arabidopsis thaliana protein match is: Galactosyltransferase family protein (TAIR:AT4G21060.1) |
| --- | --- | --- | --- | --- | --- | --- | --- | --- | --- |
|  |  | 35.2 | not assigned.unknown | Ghi.272.1.S1_s_at | Ghi.272 | 3.8451662 | DT561048 | AT1G64080.1 | unknown protein |
|  |  | 35.2 | not assigned.unknown | Ghi.1533.1.S1_s_at | Ghi.1533 | -3.7967198 | DR463368 |  |  |
|  |  | 35.2 | not assigned.unknown | GhiAffx.22562.1.A1_at | Ghi.13380 | -7.689648 | DW238476.1 | AT2G04420.1 | Polynucleotidyl transferase, ribonuclease H-like superfamily protein |
|  |  | 35.2 | not assigned.unknown | GhiAffx.38714.1.S1_at | Ghi.11105 | -3.948362 | DW492836.1 | AT3G19990.1 | unknown protein |
|  |  | 35.2 | not assigned.unknown | Ghi.6417.4.S1_s_at | Ghi.6417 | -5.504288 | CO490783 | |  |
|  |  | 35.2 | not assigned.unknown | Ghi.1660.1.S1_s_at | Ghi.1660 | -19.491045 | DN760124 | |  |
|  |  | 35.2 | not assigned.unknown | Ghi.1812.1.S1_at | Ghi.1812 | 8.004698 | DV850410 | |  |
|  |  | 35.2 | not assigned.unknown | GhiAffx.41174.1.A1_at | Ghi.1905 | -3.6899407 | DW501616.1 | |  |
|  |  | 35.2 | not assigned.unknown | Ghi.6496.1.S1_a_at | Ghi.8364 | -18.636307 | CD486227 | AT4G02380.1 | senescence-associated gene 21 |
|  |  | 35.2 | not assigned.unknown | Ghi.6693.1.A1_at | Ghi.6693 | -11.06929 | CA993655 |  |  |
|  |  | 35.2 | not assigned.unknown | Ghi.6362.1.S1_at | Ghi.6362 | -10.048435 | CO491551 | |  |
|  |  | 35.2 | not assigned.unknown | Ghi.3264.2.A1_s_at | Ghi.3264 | -7.568856 | DT463678 |  |  |
|  |  | 35.2 | not assigned.unknown | GhiAffx.32614.1.A1_x_at | | 20.266785 | CO498615 | |  |
|  |  | 35.2 | not assigned.unknown | GhiAffx.9239.1.S1_s_at | Ghi.12614 | -6.2958975 | DW244026.1 | AT2G35940.3 | BEL1-like homeodomain 1 |
|  |  | 35.2 | not assigned.unknown | GhiAffx.46297.1.S1_s_at | Ghi.6465 | -58.77188 | AI054544 | AT4G17030.1 | expansin-like B1 |
|  |  | 35.2 | not assigned.unknown | Ghi.6901.1.A1_s_at | Ghi.6901 | -5.749808 | CA992707 | AT1G27730.1 | salt tolerance zinc finger |
|  |  | 35.2 | not assigned.unknown | Ghi.699.1.S1_s_at | Ghi.699 | -10.828105 | DR456003 |  |  |
|  |  | 35.2 | not assigned.unknown | Ghi.8931.1.S1_a_at | Ghi.8931 | -57.430996 | DT457712 |  |  |
|  |  | 35.2 | not assigned.unknown | Ghi.6557.1.S1_at | Ghi.6557 | -3.270496 | CD485783 | AT2G01300.1 | unknown protein |
|  |  | 35.2 | not assigned.unknown | Ghi.1037.4.A1_x_at | Ghi.8296 | -3.4941657 | CA993008 |  |  |
|  |  | 35.2 | not assigned.unknown | Ghi.8364.1.A1_at | Ghi.8364 | -15.024564 | CA993541 | AT4G02380.1 | senescence-associated gene 21 |
|  |  | 35.2 | not assigned.unknown | Ghi.6539.1.S1_s_at | Ghi.9213 | -6.073285 | CD485942 | AT2G40000.1 | ortholog of sugar beet HS1 PRO-1 2 |
|  |  | 35.2 | not assigned.unknown | Ghi.967.2.S1_s_at | Ghi.967 | -5.1791935 | DR452676 | AT4G00230.1 | xylem serine peptidase 1 |
|  |  | 35.2 | not assigned.unknown | Ghi.1621.1.S1_x_at | Ghi.1621 | -5.960155 | DN760810 | AT3G27880.1 | Protein of unknown function (DUF1645) |
|  |  | 35.2 | not assigned.unknown | Ghi.3049.4.S1_at | Ghi.3049 | -3.4160755 | DT463685 | AT5G02230.2 | Haloacid dehalogenase-like hydrolase (HAD) superfamily protein |
|  |  | 35.2 | not assigned.unknown | GhiAffx.7806.1.S1_at | Ghi.1230 | -17.082075 | DW515985.1 | AT1G80130.1 | Tetratricopeptide repeat (TPR)-like superfamily protein |
|  |  | 35.2 | not assigned.unknown | Ghi.3335.1.A1_at | Ghi.19561 | -7.793686 | DT463623 |  |  |
