# Supplementary material for: Transcript profiling of genes expressed during fibre development in diploid cotton (Gossypium arboreum L.)
Source: BMC Genomics. 2017 Aug 31;18:675. doi: 10.1186/s12864-017-4066-y (PMC5580217; doi:10.1186/s12864-017-4066-y)
Supplement: Supplementary file 7 — Short listed view of differentially expressed transcripts present in various metabolic processes based on MapMan (version 3.5) visualization software in Gossypium arboreum fuzzy-lintless line (Fl) at 0 dpa. (DOC 37 kb) [file 12864_2017_4066_MOESM7_ESM.doc]

**Table S7: Short listed view of differentially expressed transcripts present in various metabolic processes based on MapMan (version 3.5) visualization software in *Gossypium arboreum* fuzzy-lintless line (*Fl*) at 0 dpa**.

| **Bincode** | **Name** | **Bin Details** | **Bin Description** | **Gene ID** | **Regulation** | **UniGene ID** | **GenBank ID** | **Closest Arabidopsis homolog** | **Description** |
| --- | --- | --- | --- | --- | --- | --- | --- | --- | --- |
| **10** | Cell wall | 10.7 | cell wall.modification | Ghi.10493.1.S1_s_at | 4.426241 | Ghi.17551 | DT466412 | AT5G57560.1 | Xyloglucan endotransglucosylase/hydrolase family protein |
|  |  | 10.7 | cell wall.modification | GhiAffx.21219.1.A1_s_at | 4.078241 | Ghi.9948 | DW236303.1 | AT1G69530.2 | expansin A1 |
| **34** | Transport | 34.99 | transport.misc | Ghi.7853.1.S1_at | -5.82831 | Ghi.16284 | AF443118.1 | AT1G01630.1 | Sec14p-like phosphatidylinositol transfer family protein |
| **16** | Secondary metabolism | | |  |  |  |  |  |  |
| **30** | Signalling | 30.1 | signalling.in sugar and nutrient physiology | GhiAffx.63620.1.S1_at | 4.962863 | Ghi.15977 | DW505500.1 | AT5G55850.3 | RPM1-interacting protein 4 (RIN4) family protein |
|  |  | 30.3 | signalling.calcium | GhiAffx.24550.1.S1_at | 5.723753 | Ghi.16133 | DN818231 | AT4G27280.1 | Calcium-binding EF-hand family protein |
|  |  | 30.3 | signalling.calcium | Ghi.3763.1.A1_s_at | 7.196658 | Ghi.3763 | DT461952 | AT3G63380.1 | ATPase E1-E2 type family protein / haloacid dehalogenase-like hydrolase family protein |
| **35** | Not assigned | 35.2 | not assigned.unknown | Ghi.2608.2.A1_at | 3.616642 | Ghi.2608 | DT463212 | AT3G55840.1 | Hs1pro-1 protein |
| **20** | Stress | 20.2.1 | stress.abiotic.heat | Ghi.10778.2.S1_at | 3.257512 | Ghi.10778 | CA993737 | AT2G17880.1 | Chaperone DnaJ-domain superfamily protein |
| **22** | Polyamine metabolism | 22.1.6 | polyamine metabolism.synthesis.spermidine synthase | GhiAffx.2527.1.S1_s_at | -151.187 | Ghi.13939 | DW497370.1 | AT5G53120.6 | spermidine synthase 3 |
