# Supplementary material for: Transcript profiling of genes expressed during fibre development in diploid cotton (Gossypium arboreum L.)
Source: BMC Genomics. 2017 Aug 31;18:675. doi: 10.1186/s12864-017-4066-y (PMC5580217; doi:10.1186/s12864-017-4066-y)
Supplement: Supplementary file 8 — Short listed view of differentially expressed transcripts present in various metabolic processes based on MapMan (version 3.5) visualization software in Gossypium arboreum fuzzy-lintless line (Fl) at 10 dpa. (DOC 93 kb) [file 12864_2017_4066_MOESM8_ESM.doc]

**Table S8: Short listed view of differentially expressed transcripts present in various metabolic processes based on MapMan (version 3.5) visualization software in *Gossypium arboreum* fuzzy-lintless line (*Fl*) at 10 dpa.**

| **Bincode** | **Name** | **Bin Details** | **BIN Description** | **GENE ID** | **UniGene ID** | **REGULATION** | **GenBank ID** | **Closest Arabidopsis homolog** | **Description** |
| --- | --- | --- | --- | --- | --- | --- | --- | --- | --- |
| **3** | Minor CHO metabolism | 3.2.2 | minor CHO metabolism.trehalose.TPP | Ghi.912.3.A1_at | Ghi.912 | -4.70675 | DT468062 | AT5G65140.1 | Haloacid dehalogenase-like hydrolase (HAD) superfamily protein |
|  |  | 3.2.3 | minor CHO metabolism.trehalose.potential TPS/TPP | Ghi.3451.2.A1_at | Ghi.3451 | -3.80554 | DT465672 | AT1G23870.1 | trehalose-phosphatase/synthase 9 |
| **10** | Cell wall | 10.1.2 | cell wall.precursor synthesis.UGE | GhiAffx.19125.1.A1_at | | -19.1726 | DW487672.1 | AT1G12780.1 | UDP-D-glucose/UDP-D-galactose 4-epimerase 1 |
|  |  | 10.1.2 | cell wall.precursor synthesis.UGE | Gra.2050.1.S1_s_at | Gra.2050 | -3.23173 | CO126131 | AT1G12780.1 | UDP-D-glucose/UDP-D-galactose 4-epimerase 1 |
|  |  | 10.6 | cell wall.degradation | GhiAffx.60253.1.S1_at | Ghi.14666 | -3.0274 | DW503386.1 | AT5G62150.1 | peptidoglycan-binding LysM domain-containing protein |
|  |  | 10.6.2 | cell wall.degradation.mannan-xylose-arabinose-fucose | Ghi.8524.1.S1_at | Ghi.18592 | -3.02125 | DT048651 | AT5G49360.1 | beta-xylosidase 1 |
|  |  | 10.6.3 | cell wall.degradation.pectate lyases and polygalacturonases | GhiAffx.7814.1.S1_s_at | Ghi.21981 | -3.70053 | DW516033.1 | AT1G49320.1 | unknown seed protein like 1 |
|  |  | 10.7 | cell wall.modification | Ghi.4532.1.A1_at | Ghi.4532 | -5.76091 | DR457588 | AT4G18990.1 | xyloglucan endotransglucosylase/hydrolase 29 |
|  |  | 10.7 | cell wall.modification | Ghi.6465.2.S1_at | Ghi.6465 | -55.0491 | CD485906 | AT4G17030.1 | expansin-like B1 |
|  |  | 10.7 | cell wall.modification | Ghi.6236.2.S1_s_at | Ghi.6236 | 7.36092 | D88413.1 | AT4G37800.1 | xyloglucan endotransglucosylase/hydrolase 7 |
|  |  | 10.7 | cell wall.modification | GraAffx.27319.1.S1_s_at | | 3.155124 | CO089724 | AT2G36870.1 | xyloglucan endotransglucosylase/hydrolase 32 |
|  |  | 10.7 | cell wall.modification | Ghi.6236.1.S1_s_at | Ghi.6236 | 5.846538 | AY189971.1 | AT4G37800.1 | xyloglucan endotransglucosylase/hydrolase 7 |
|  |  | 10.7 | cell wall.modification | Ghi.4725.1.S1_s_at | Ghi.4725 | -3.68657 | AI726805 | AT5G13870.1 | xyloglucan endotransglucosylase/hydrolase 5 |
|  |  | 10.7 | cell wall.modification | Gra.377.1.A1_s_at | Gra.2265 | -3.41328 | CO085937 | AT2G06850.1 | xyloglucan endotransglucosylase/hydrolase 4 |
|  |  | 10.7 | cell wall.modification | GraAffx.8958.1.S1_s_at | | 6.785116 | CO087973 | AT4G37800.1 | xyloglucan endotransglucosylase/hydrolase 7 |
|  |  | 10.8.2 | cell wall.pectin*esterases.acetyl esterase | Ghi.5860.1.S1_s_at | Ghi.16418 | 46.18646 | CO494172 | AT2G04780.2 | FASCICLIN-like arabinoogalactan 7 |

| **31** | Cell | 31.1 | cell.organisation | Ghi.468.1.A1_s_at | Ghi.16435 | -3.86802 | AY189970.1 | AT2G19770.1 | profilin 5 |
| --- | --- | --- | --- | --- | --- | --- | --- | --- | --- |
|  |  | 31.1 | cell.organisation | GhiAffx.33535.1.S1_at | | -3.01488 | AI727184 | AT2G16700.2 | actin depolymerizing factor 5 |
|  |  | 31.1 | cell.organisation | Ghi.8448.1.S1_x_at | Ghi.8448 | -11.6073 | AF521240.1 | AT5G12250.1 | beta-6 tubulin |
| **11** | Lipid metabolism | 11.1.8 | lipid metabolism.FA synthesis and FA elongation.acyl coa ligase | Ghi.3184.1.S1_s_at | Ghi.1202 | -4.25064 | DT468147 | AT3G16910.1 | acyl-activating enzyme 7 |
|  |  | 11.9.2 | lipid metabolism.lipid degradation.lipases | GhiAffx.24021.1.S1_at | | -3.3666 | DW509098.1 | AT2G44970.2 | alpha/beta-Hydrolases superfamily protein |
|  |  | 11.9.2 | lipid metabolism.lipid degradation.lipases | Ghi.7293.1.S1_s_at | Ghi.7293 | -3.39789 | AI731856 | AT2G39420.1 | alpha/beta-Hydrolases superfamily protein |
|  |  | 11.9.4.2 | lipid metabolism.lipid degradation.beta-oxidation.acyl CoA DH | Ghi.7466.1.S1_at | Ghi.7466 | -3.14161 | AI730393 | AT3G51840.1 | acyl-CoA oxidase 4 |
| **26** | Misc | 26.18 | misc.invertase/pectin methylesterase inhibitor family protein | Ghi.1552.1.S1_s_at | Ghi.1552 | -18.2003 | DN779868 | AT1G47960.1 | cell wall / vacuolar inhibitor of fructosidase 1 |
|  |  | 26.21 | misc.protease inhibitor/seed storage/lipid transfer protein (LTP) family protein | Gra.378.1.A1_s_at | Gra.378 | -3.13665 | CO085861 | AT3G53980.2 | Bifunctional inhibitor/lipid-transfer protein/seed storage 2S albumin superfamily protein |
| **16** | Secondary metabolism | 16.1.4.6 | secondary metabolism.isoprenoids.carotenoids.carotenoid beta ring hydroxylase | GhiAffx.17151.1.S1_at | Ghi.19974 | -3.68604 | DW243562.1 | AT5G52570.1 | beta-carotene hydroxylase 2 |
|  |  | 16.1.5 | secondary metabolism.isoprenoids.terpenoids | GarAffx.37202.1.S1_x_at | | -3.50249 | U23205.1 | AT5G23960.2 | terpene synthase 21 |
|  |  | 16.2 | secondary metabolism.phenylpropanoids | GhiAffx.15490.1.S1_at | Ghi.18655 | -3.63275 | DW237402.1 | AT2G22570.1 | nicotinamidase 1 |
|  |  | 16.2 | secondary metabolism.phenylpropanoids | Ghi.5521.1.A1_s_at | Ghi.5521 | -8.33038 | DT047436 | AT5G01210.1 | HXXXD-type acyl-transferase family protein |
|  |  | 16.2.1.6 | secondary metabolism.phenylpropanoids.lignin biosynthesis.CCoAOMT | Ghi.4349.1.A1_at | Ghi.4349 | -5.01652 | DT054063 | AT1G67980.2 | caffeoyl-CoA 3-O-methyltransferase |
|  |  | 16.4.1 | secondary metabolism.N misc.alkaloid-like | Ghi.8033.1.S1_s_at | Ghi.16611 | -8.29359 | DQ122187.1 | AT2G20340.1 | Pyridoxal phosphate (PLP)-dependent transferases superfamily protein |
|  |  | 16.8.1.21 | secondary metabolism.flavonoids.anthocyanins.anthocyanin 5-aromatic acyltransferase | Ghi.1935.1.S1_at | Ghi.1935 | -7.59902 | DV848993 | AT3G29590.1 | HXXXD-type acyl-transferase family protein |
|  |  | 16.8.4 | secondary metabolism.flavonoids.flavonols | GhiAffx.1402.1.S1_at | Ghi.14499 | -3.43616 | DW231451.1 | AT1G15550.1 | gibberellin 3-oxidase 1 |
| **30** | Signalling | 30.2.2 | signalling.receptor kinases.leucine rich repeat II | Ghi.44.1.A1_at | Ghi.44 | -3.3454 | DR463380 | AT5G10290.1 | leucine-rich repeat transmembrane protein kinase family protein |
|  |  | 30.2.11 | signalling.receptor kinases.leucine rich repeat XI | GbaAffx.197.1.S1_s_at | | -4.19841 | AY279356.1 | AT5G06860.1 | polygalacturonase inhibiting protein 1 |

|  |  | 30.2.17 | signalling.receptor kinases.DUF 26 | GhiAffx.3185.1.S1_at | Ghi.17263 | -76.7069 | DW514553.1 | AT1G78860.1 | D-mannose binding lectin protein with Apple-like carbohydrate-binding domain |
| --- | --- | --- | --- | --- | --- | --- | --- | --- | --- |
|  |  | 30.6 | signalling.MAP kinases | Ghi.6088.2.A1_s_at | Ghi.6088 | -10.7548 | DV849489 | AT3G45640.1 | mitogen-activated protein kinase 3 |
|  |  | 30.6 | signalling.MAP kinases | Ghi.5022.4.A1_s_at | Ghi.17602 | -5.89073 | DT049392 | AT1G73500.1 | MAP kinase kinase 9 |
| **26** | Misc | 26.12 | misc.peroxidases | Ghi.8105.1.A1_s_at | Ghi.8105 | -7.12393 | AF488305.1 | AT5G06720.1 | peroxidase 2 |
|  |  | 26.12 | misc.peroxidases | Ghi.7950.1.S1_at | Ghi.16267 | -81.1664 | AY366083.1 | AT5G06720.1 | peroxidase 2 |
|  |  | 26.12 | misc.peroxidases | Ghi.1043.4.S1_at | Ghi.17859 | -8.14276 | DT463348 | AT5G05340.1 | Peroxidase superfamily protein |
|  |  | 26.12 | misc.peroxidases | Ghi.8110.1.S1_at | Ghi.8110 | -4.90623 | AY311597.1 | AT5G05340.1 | Peroxidase superfamily protein |
| **35** | Not assigned | 35.2 | not assigned.unknown | Ghi.2608.2.A1_at | Ghi.2608 | -5.85847 | DT463212 | AT3G55840.1 | Hs1pro-1 protein |
|  |  | 35.2 | not assigned.unknown | GhiAffx.5935.2.S1_s_at | Ghi.9213 | -7.22764 | DW235907.1 | AT3G55840.1 | Hs1pro-1 protein |
|  |  | 35.2 | not assigned.unknown | Ghi.6496.1.S1_a_at | Ghi.8364 | -18.6363 | CD486227 | AT4G02380.1 | senescence-associated gene 21 |
|  |  | 35.2 | not assigned.unknown | Ghi.8364.1.A1_at | Ghi.8364 | -15.0246 | CA993541 | AT4G02380.1 | senescence-associated gene 21 |
| **20** | Stress | 20.1.7 | stress.biotic.PR-proteins | Ghi.3370.1.A1_at | Ghi.3370 | -45.2945 | DT463939 | AT1G33590.1 | Leucine-rich repeat (LRR) family protein |
|  |  | 20.1.7 | stress.biotic.PR-proteins | Ghi.490.1.S1_s_at | Ghi.490 | -14.9219 | DT465033 | AT1G33590.1 | Leucine-rich repeat (LRR) family protein |
|  |  | 20.1.7 | stress.biotic.PR-proteins | Ghi.3370.1.S1_s_at | Ghi.3370 | -4.83059 | DT466783 | AT1G33590.1 | Leucine-rich repeat (LRR) family protein |
|  |  | 20.2.1 | stress.abiotic.heat | GhiAffx.16191.1.S1_at | Ghi.11788 | -4.58866 | DW507100.1 | AT2G29500.1 | HSP20-like chaperones superfamily protein |
|  |  | 20.2.1 | stress.abiotic.heat | Ghi.6780.1.S1_s_at | Ghi.6780 | -15.1656 | CA993199 | AT2G17880.1 | Chaperone DnaJ-domain superfamily protein |
| **22** | Polyamine metabolism | 22.1.6 | polyamine metabolism.synthesis.spermidine synthase | GhiAffx.2527.1.S1_s_at | Ghi.13939 | -168.975 | DW497370.1 | AT5G53120.6 | spermidine synthase 3 |
